# Percentage of Heavy Drinking Days Following Psilocybin-Assisted Psychotherapy vs Placebo in the Treatment of Adult Patients With Alcohol Use Disorder: A Randomized Clinical Trial

**DOI:** 10.1001/jamapsychiatry.2022.2096

**Published:** 2022-08-24

**Authors:** Michael P. Bogenschutz, Stephen Ross, Snehal Bhatt, Tara Baron, Alyssa A. Forcehimes, Eugene Laska, Sarah E. Mennenga, Kelley O’Donnell, Lindsey T. Owens, Samantha Podrebarac, John Rotrosen, J. Scott Tonigan, Lindsay Worth

**Affiliations:** 1 Department of Psychiatry, New York University Langone Center for Psychedelic Medicine, New York University Grossman School of Medicine, New York; 2 Department of Psychiatry and Behavioral Sciences, University of New Mexico School of Medicine, Albuquerque; 3 The Change Companies, Carson City, Nevada; 4 Department of Population Health, Division of Biostatistics, New York University Grossman School of Medicine, New York; 5 Department of Psychology, University of Alabama at Birmingham; 6 University of New Mexico Center on Alcohol, Substance Use and Addictions, Albuquerque

## Abstract

**Importance:**

Although classic psychedelic medications have shown promise in the treatment of alcohol use disorder (AUD), the efficacy of psilocybin remains unknown.

**Objective:**

To evaluate whether 2 administrations of high-dose psilocybin improve the percentage of heavy drinking days in patients with AUD undergoing psychotherapy relative to outcomes observed with active placebo medication and psychotherapy.

**Design, Setting, and Participants:**

In this double-blind randomized clinical trial, participants were offered 12 weeks of manualized psychotherapy and were randomly assigned to receive psilocybin vs diphenhydramine during 2 day-long medication sessions at weeks 4 and 8. Outcomes were assessed over the 32-week double-blind period following the first dose of study medication. The study was conducted at 2 academic centers in the US. Participants were recruited from the community between March 12, 2014, and March 19, 2020. Adults aged 25 to 65 years with a *DSM-IV* diagnosis of alcohol dependence and at least 4 heavy drinking days during the 30 days prior to screening were included. Exclusion criteria included major psychiatric and drug use disorders, hallucinogen use, medical conditions that contraindicated the study medications, use of exclusionary medications, and current treatment for AUD.

**Interventions:**

Study medications were psilocybin, 25 mg/70 kg, vs diphenhydramine, 50 mg (first session), and psilocybin, 25-40 mg/70 kg, vs diphenhydramine, 50-100 mg (second session). Psychotherapy included motivational enhancement therapy and cognitive behavioral therapy.

**Main Outcomes and Measures:**

The primary outcome was percentage of heavy drinking days, assessed using a timeline followback interview, contrasted between groups over the 32-week period following the first administration of study medication using multivariate repeated-measures analysis of variance.

**Results:**

A total of 95 participants (mean [SD] age, 46 [12] years; 42 [44.2%] female) were randomized (49 to psilocybin and 46 to diphenhydramine). One participant (1.1%) was American Indian/Alaska Native, 3 (3.2%) were Asian, 4 (4.2%) were Black, 14 (14.7%) were Hispanic, and 75 (78.9%) were non-Hispanic White. Of the 95 randomized participants, 93 received at least 1 dose of study medication and were included in the primary outcome analysis. Percentage of heavy drinking days during the 32-week double-blind period was 9.7% for the psilocybin group and 23.6% for the diphenhydramine group, a mean difference of 13.9%; (95% CI, 3.0–24.7; *F*_1,86_ = 6.43; *P* = .01). Mean daily alcohol consumption (number of standard drinks per day) was also lower in the psilocybin group. There were no serious adverse events among participants who received psilocybin.

**Conclusions and Relevance:**

Psilocybin administered in combination with psychotherapy produced robust decreases in percentage of heavy drinking days over and above those produced by active placebo and psychotherapy. These results provide support for further study of psilocybin-assisted treatment for AUD.

**Trial Registration:**

ClinicalTrials.gov Identifier: NCT02061293

## Key Points

**Question:**

Does psilocybin-assisted treatment improve drinking outcomes in patients with alcohol use disorder relative to outcomes observed with active placebo medication?

**Findings:**

In this double-blind randomized clinical trial with 93 participants, the percentage of heavy drinking days during 32 weeks of follow-up was significantly lower in the psilocybin group than in the diphenhydramine group.

**Meaning:**

The results in this trial showed that psilocybin administered in combination with psychotherapy produced robust decreases in the percentage of heavy drinking days compared with those produced by active placebo and psychotherapy.

## Teaser

This randomized clinical trial evaluates the combination of psychotherapy with psilocybin vs active placebo in patients with alcohol use disorder.

## Introduction

The past 2 decades have witnessed growing interest in the clinical potential of psilocybin and other classic psychedelics to treat neuropsychiatric conditions, including substance use disorders.^1,2,3,4,5,6,7,8^ Although the mechanisms of psychedelic-assisted treatments remain unclear, the action of these drugs at the serotonin 2A receptor and downstream effects on neurotransmission, intracellular signaling, epigenetics, and gene expression appear to enhance plasticity at multiple levels, including neuronal structure, neural networks, cognition, affect, and behavior.^9,10,11,12,13,14,15,16,17,18,19,20,21,22,23,24^ However, some clinically relevant effects may be independent of serotonin 2A receptor activation.^24,25^ Moreover, the direction and magnitude of change observed in a therapeutic context can be influenced by the subjective experience under the influence of the drug^26,27,28,29^ and by contextual factors, including concomitant psychotherapy.^30,31,32^

Alcohol use disorder (AUD) is a particularly promising target for treatment with psychedelics. A meta-analysis of results from 6 randomized clinical trials published between 1966 and 1971^33,34,35,36,37,38^ revealed that participants with alcohol dependence treated with lysergic acid diethylamide (LSD) demonstrated remission during follow-up nearly twice as often as those in comparator conditions to (odds ratio, 1.96, 95% CI, 1.36-2.84; *z*, 3.59; *P* < .001).^39^ Picking up on this line of research after a hiatus of more than 40 years, an open-label study published in 2015 demonstrated that moderately high doses of psilocybin (21 to 28 mg/70 kg) were well tolerated by participants with alcohol dependence, and large reductions in drinking were observed over a 32-week follow-up period.^3^

Building on the proof-of-concept study, this multisite randomized clinical trial evaluated the efficacy of psilocybin-assisted psychotherapy for the treatment of AUD. Here we report drinking outcomes for the double-blind phase of the trial.

## Methods

### Trial Oversight

The study was reviewed and approved by the Heffter Research Institute, the institutional review boards of each site (New York University Grossman School of Medicine and the University of New Mexico Health Sciences Center), the US Food and Drug Administration and Drug Enforcement Administration, the New Mexico Board of Pharmacy, and the New York State Bureau of Narcotics Enforcement. Psilocybin was provided by the Usona Institute, Madison, Wisconsin, Nicholas Cozzi, PhD, at the University of Wisconsin–Madison, and David Nichols, PhD, at Purdue University, West Lafayette, Indiana. The study was overseen by a data and safety monitoring board. One of the authors (M.P.B.) .was the investigational new drug application holder for the trial. This report followed the Consolidated Standards of Reporting Trials (CONSORT) reporting guideline for parallel-group randomized trials. All participants provided written informed consent. The trial protocol and statistical analysis plan can be found in Supplement 1.

### Participants

Participants were recruited from March 12, 2014, to May 13, 2015, at the University of New Mexico and from July 9, 2015, to March 19, 2020, at New York University, using advertisements in local media. Participants were aged 25 to 65 years, had a diagnosis of alcohol dependence ascertained using the Structured Clinical Interview for *DSM-IV*,^40^ and had at least 4 heavy drinking days during the 30 days prior to screening (defined as 5 or more drinks in a day for a man and 4 or more drinks in a day for a woman). Exclusion criteria included major psychiatric and drug use disorders, any hallucinogen use in the past year or more than 25 lifetime uses, medical conditions that contraindicated either of the study medications, use of exclusionary medications, and current treatment for AUD. Race and ethnicity were determined by participant self-report according to standard National Institutes of Health categories in order to assess the representativeness of the sample. The trial protocol in Supplement 1 describes full inclusion and exclusion criteria.

### Trial Design

#### Overview

Qualifying participants were assessed at screening, baseline (week 0), and weeks 4, 5, 8, 9, 12, 24, and 36. They were randomly assigned in a 1:1 ratio to receive either psilocybin or diphenhydramine, administered in two 8-hour sessions at weeks 4 and 8. All participants who completed the double-blind observation period (weeks 5 to 36) and still met safety criteria were offered an open-label psilocybin session at week 38, including 4 additional psychotherapy sessions and assessment for an additional 18 weeks. Participants received up to a total of $560 for completing assessments in the course of the trial but were not reimbursed for attending the therapy and medication sessions.

#### Psychotherapeutic Elements of Treatment

All participants were offered a total of 12 psychotherapy sessions from a team of 2 therapists, including a licensed psychiatrist: 4 before the first medication session, 4 between the first and second medication sessions, and 4 in the month following the second medication session. The psychotherapy, described in detail in a separate publication,^41^ included motivational interviewing and cognitive behavioral therapy for AUD as well as material designed to help the participants to manage and make use of the psychoactive effects of the study medication. Training, supervision, and fidelity monitoring procedures are described in the protocol in Supplement 1.

#### Randomization and Blinding

Randomization was stratified by site and consisted of balanced blocks of varying size. A study pharmacist at each site generated the randomization sequence and assigned treatment in order of randomization. All other study staff and investigators as well as participants were blinded to treatment assignment.

#### Dosage of Study Medication

Study medication was taken orally in a single opaque capsule of unvarying appearance and weight. Psilocybin doses were weight based to control for participant body weight, which ranged from 49.0 to 116.1 kg (mean [SD], 78.3 [15.6] kg). Doses for the first session were psilocybin, 25 mg/70 kg, or diphenhydramine, 50 mg. Participants received an increased dose in the second session if there were no dose-limiting adverse events and they agreed to the increase. The increased dose of psilocybin was 30 mg/70 kg if the participant’s total score on the Pahnke-Richards Mystical Experience Questionnaire (MEQ)^42^ was 0.6 or greater in the first session (indicating a robust subjective response to the 25 mg/70 kg dose) or 40 mg/70 kg if the MEQ total score in the first session was less than 0.6. The increased dose of diphenhydramine was 100 mg regardless of subjective response.

#### Administration of Study Medication

Study medication was administered at approximately 9 am, after which participants were required to stay in the session room with the therapists for at least 8 hours (except for bathroom breaks). During the session, participants were encouraged to lie on a couch wearing eyeshades and headphones providing a standardized playlist of music. Medications were available in the session room to treat hypertension, severe anxiety, or psychotic symptoms as specified in the protocol.

### Outcomes and Assessments

#### Subjective Effects of Study Medication

Subjective effects of psilocybin vs diphenhydramine were assessed using the States of Consciousness Questionnaire,^42^ containing the 43-item MEQ. This questionnaire was completed immediately after each medication session.

#### Drinking Outcomes

The prespecified primary drinking outcome was the percentage of heavy drinking days (PHDD) during weeks 5 to 32, assessed at weeks 8, 12, 24, and 36 using timeline followback, a reliable and valid calendar-based method, which is the criterion standard outcome for AUD clinical trials.^43,44,45,46,47^ One standard drink was defined as 14 g of ethanol. Secondary outcomes included percentage of drinking days (PDD), mean drinks per day (DPD), and dichotomous outcomes: abstinence, defined following a recent study^48^ evaluating the use of WHO risk levels as a treatment outcome; lack of heavy drinking days; and reduction in World Health Organization (WHO) risk level^49^ by 1, 2, or 3 levels. Hair or fingernail samples were collected at week 24 and assayed for ethylglucuronide (EtG) concentration to confirm self-reported abstinence. The Short Index of Problems (SIP-2R)^50^ was used to assess drinking-related problems at baseline and at weeks 12, 24, and 36.

#### Safety and Blinding Integrity

Blood pressure and heart rate were assessed at 30- to 60-minute intervals during the first 6 hours of each medication session. Adverse events were solicited at each postscreening assessment. After each session, participants and therapists were asked to guess which medication had been administered and rate their degree of certainty on a 100-point visual analog scale (0 = not at all confident; 100 = extremely confident).

### Statistical Analysis

The statistical analysis plan was developed in accordance with published guidelines^51^ and contains a full description of statistical methods. The statistical analysis plan can be found in Supplement 1.

#### Sample Size and Power

The study was originally designed to randomize up to 180 participants. An interim analysis was planned after recruitment of 100 participants to reestimate the necessary sample size to yield power of 0.8 to detect a small to moderate effect (*f*^2^ = 0.16) with no correction for multiple comparisons. However, following an indefinite mandatory suspension of recruitment beginning on March 19, 2020, due to the outbreak of COVID-19, enrollment for this trial was halted at 95 randomized participants.

#### Subjective Effects and Efficacy

MEQ scores for the first and second medication sessions were computed and contrasted by group (psilocybin vs diphenhydramine) using *t* tests for independent samples. To evaluate the effects of treatment on continuous drinking outcomes (PHDD, PDD, and DPD), 3-dimensional multivariate repeated-measures analysis of variance was used, including fixed categorical effects of treatment, assessment, and site; site-by-treatment and treatment-by-assessment interactions; fixed baseline covariates for each dependent measure (PHDD, PDD, and DPD during weeks 1 to 4); and monthly values of PHDD, PDD, and DPD (weeks 5 to 8, 9 to 12, 13 to 16, 17 to 20, 21 to 24, 25 to 28, 29 to 32, and 32 to 36) as a nested multivariate dependent measure. All missing monthly values of PHDD, PDD, and DPD were imputed simultaneously using Multivariate Imputation by Chained Equations in R (MICE) version 3.14.0 (R Foundation).^52^ Significant multivariate treatment effects were decomposed with univariate repeated-measures *F* tests within each drinking dimension (PHDD, PDD, and DPD).^53^

Treatment contrasts for dichotomous outcomes were obtained using χ^2^ statistics. Effects of treatment on problems related to drinking were compared using univariate mixed models for repeated measures and generalized linear models. Hedges *g* was computed as a measure of effect size for between- and within-group differences on continuous outcomes, and odds ratios were computed for dichotomous outcomes. No correction was made for multiple comparisons, so analyses of secondary outcomes should be considered exploratory.

#### Safety Outcomes

Blood pressure and heart rate treatment contrasts were based on mixed models for repeated measures with fixed categorical effects of treatment and assessment, a treatment-by-assessment interaction, and a fixed covariate (value of each outcome prior to drug administration). All adverse events occurring after informed consent were coded according to the Medical Dictionary for Regulatory Activities and tabulated, and prevalence within treatment groups (proportion of participants affected) was compared using Fisher exact tests. Two-sided *P* < .05 was considered statistically significant.

## Results

### Participants

**Figure 1.**
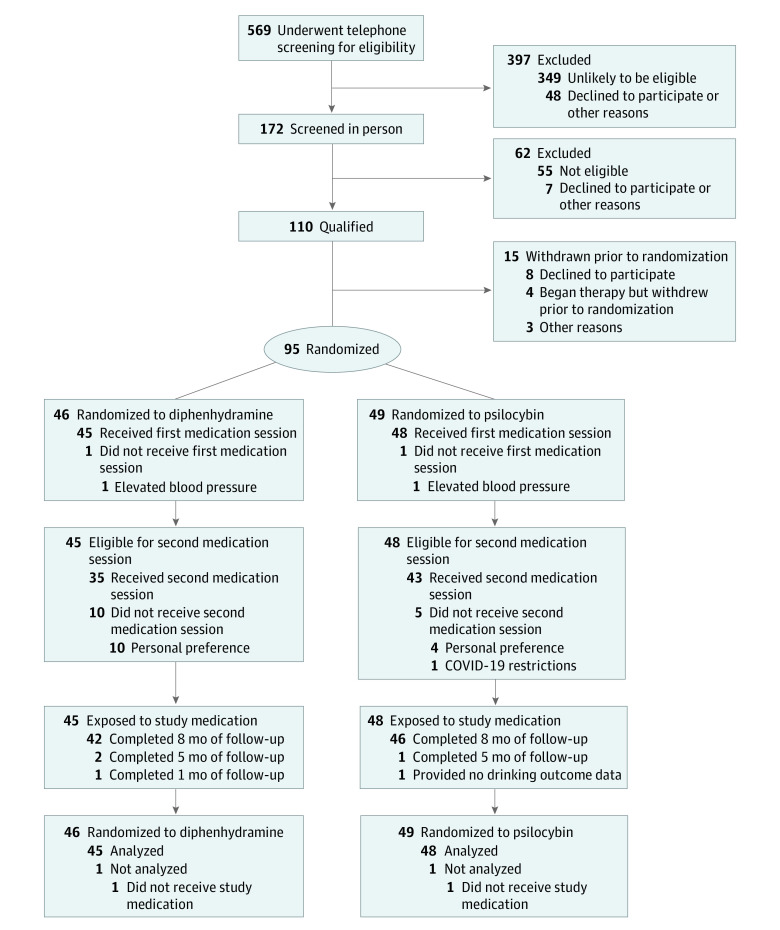
CONSORT Diagram

Figure 1 summarizes recruitment of participants, treatment exposure, and retention. A total of 95 participants were randomized: 49 to psilocybin and 46 to diphenhydramine. Table 1 describes baseline characteristics of the randomized sample. The mean (SD) age was 45.8 (11.6) years, and 42 participants (44.2%) were female. One participant (1.1%) was American Indian/Alaska Native, 3 (3.2%) were Asian, 4 (4.2%) were Black, 14 (14.7%) were Hispanic, and 75 (78.9%) were non-Hispanic White (sum is greater than 100% due to multiple categories selected by 2 participants). Participants met a mean (SD) 5.3 (1.2) of the 7 alcohol dependence criteria and had been alcohol dependent for a mean (SD) 14.2 (9.7) years. During the 12 weeks prior to screening, they consumed alcohol a mean (SD) 74.9% (28.1%) of days, including heavy consumption on a mean (SD) 52.7% (30.58) of days, and consuming a mean (SD) 7.1 (4.1) standard drinks per drinking day.

**Table 1.** Participant Characteristics

	Mean (SD)		
	Total	Diphenhydramine	Psilocybin
No.	95	46	49
Demographic characteristics			
Age, y	45.78 (11.56)	44.24 (12.15)	47.18 (10.93)
Household income, median (range), $	100 000 (3700-4 000 000)	110 000 (8000-800 000)	100 000 (3700-4 000 000)
Sex			
Female	42 (44.2)	21 (45.7)	21 (42.9)
Male	53 (55.8)	25 (54.3)	28 (57.1)
Race and ethnicity, No. (%)^a^			
American Indian/Alaska Native	1 (1.1)	1 (2.2)	0
Asian	3 (3.2)	0	3 (6.1)
Black	4 (4.2)	1 (2.2)	3 (6.1)
Hispanic	14 (14.7)	8 (17.4)	6 (12.2)
Non-Hispanic White	75 (78.9)	37 (80.4)	38 (77.6)
Drinking-related characteristics			
% Drinking days	74.85 (28.06)	71.00 (29.02)	78.47 (26.92)
% Heavy drinking days	52.71 (30.58)	47.93 (28.74)	57.20 (31.84)
Drinks per day	4.78 (2.62)	4.33 (2.39)	5.20 (2.78)
Drinks per drinking day	7.10 (4.05)	6.64 (3.37)	7.52 (4.58)
No. of dependence criteria^b^	5.25 (1.22)	5.41 (1.20)	5.10 (1.23)
Age at onset, y	31.42 (11.42)	30.96 (12.03)	31.86 (10.92)
Years dependent	14.20 (9.68)	13.00 (10.31)	15.33 (9.00)
Short Index of Problems (total score)	20.98 (9.15)	21.60 (9.61)	20.26 (8.89)
WHO risk category, No. (%)^c^			
Very high	30 (31.6)	12 (26.1)	18 (36.7)
High	32 (33.7)	15 (32.6)	17 (34.7)
Moderate	21 (22.1)	12 (26.1)	9 (18.4)
Low	12 (12.6)	7 (15.2)	5 (10.2)

Abbreviation: WHO, World Health Organization.

^a^ Race and ethnicity were determined by participant self-report according to standard National Institutes of Health categories in order to assess the representativeness of the sample. Sum is greater than 100% due to multiple categories selected by 2 participants.

^b^ Defined using the Structured Clinical Interview for DSM-IV axis I disorders.^40^

^c^ WHO risk categories are defined as follows. Abstinence was defined as no risk (level 0), following a recent study^48^ evaluating the use of WHO risk levels as a treatment outcome. For men, low risk (level 1) is defined as >0 g/d to ≤40 g/d; moderate risk (level 2) as >40 g/d to ≤60 g/d; high risk (level 3) as >60 g/d to ≤100 g/d; and very high risk (level 4) as >100 g/d. For women, low risk (level 1) is defined as >0 g/d to ≤20 g/d; moderate risk (level 2) as >20 g/d to ≤40 g/d; high risk (level 3) as >40 g/d to ≤60 g/d; and very high risk (level 4) as >60 g/d. Change in WHO risk level was calculated in relation to drinking during the 12 weeks prior to screening.

### Treatment Exposure and Retention

Participation in the nonmedication therapy sessions was high and did not substantially differ between treatment groups. Participants treated with psilocybin and diphenhydramine completed a mean (SD) 11.75 (0.76) and 11.47 (1.20) of the 12 sessions, respectively (*F*_1,91_ = 1.88; *P* = .17). Of 95 participants randomized, 93 received at least 1 dose of medication: 48 received psilocybin (25 mg/70 kg) and 45 received diphenhydramine (50 mg) in the first medication session. Forty-three of participants treated with psilocybin (89.6%) and 35 of those treated with diphenhydramine (77.8%) received a second double-blind medication session (*F*_1,91_ = 2.40; *P* = .13). In the second session, psilocybin doses were 25 mg/70 kg (n = 1), 30 mg/70 kg (n = 27), and 40 mg/70 kg (n = 15), and diphenhydramine doses were 50 mg (n = 11) and 100 mg (n = 24). Mean (SD; range) absolute dosages of psilocybin were 28.3 (5.4; 19.3-40.0) mg for psilocybin session 1 and 37.7 (8.6; 24.1-64.5) mg for psilocybin session 2.

Valid drinking outcome data were obtained for 717 of 744 months (96.4%) in the 8-month follow-up period for the 93 participants receiving treatment (366 of 384 [95.3%] in the psilocybin group and 351 of 360 [97.5%] in the diphenhydramine group). A total of 63 of 337 follow-up TLFB assessments (18.7%) were collected by phone due to inability to complete in-person visits. EtG results were available for 50 of 93 participants (53.8%), with missing data due to telephone visits (n = 24), insufficient hair samples (n = 12), missing visits (n = 5), or other reasons (n = 2). Participants missing EtG data did not differ from other participants on baseline drinking measures, age, race, ethnicity, or sex.

### Blinding Integrity

Participants correctly guessed their treatment assignment in 93.6% of the first sessions, reporting a mean (SD) certainty of 88.5% (23.2%). In the second session, 94.7% guessed correctly, and mean (SD) certainty was 90.6% (21.5%). Study therapists correctly guessed treatment 92.4% of the time for first sessions and 97.4% for second sessions, and their mean (SD) certainties were 92.8% (16.3%) and 95.4% (2.9%), respectively.

### Convergent Validity of Self-report and EtG

Among the 50 participants for whom valid EtG results were obtained at week 24, 14 (28%) reported total abstinence on the week 24 TLFB. EtG results were negative (less than 8 pg/ng) for all of these participants, providing some objective support for the veracity of self-report in this sample.

### Acute Effects

#### Cardiovascular Effects

Psilocybin administration was associated with increased systolic and diastolic blood pressure relative to diphenhydramine (eFigure in Supplement 2), but no participant reported symptoms or was treated for hypertension. By 360 minutes, blood pressure was no longer significantly elevated. Heart rate was also higher in the psilocybin group until approximately 300 minutes after drug administration.

#### Subjective Effects

Mean (SD) MEQ scores for session 1 were 0.59 (0.24) in participants treated with psilocybin vs 0.10 (0.13) in those receiving diphenhydramine (*t*_1,74.3_ = 12.41; *P* < .001). For session 2, mean (SD) scores were 0.64 (0.21) vs 0.11 (0.16), respectively (*t*_1,75.5_ = 13.01; *P* < .001). These scores indicate high average intensity of experiences in the psilocybin group and low average intensity in the diphenhydramine group.

### Changes in Drinking Prior to Randomization

Substantial decreases in PHDD, PDD, and DPD were observed in both treatment groups between screening and week 4, during which time participants received 4 psychotherapy sessions and attempted to stop drinking in preparation for the first medication session (Table 2). Among participants who subsequently received psilocybin, PHDD decreased by a mean of 32.37 (95% CI, 23.68-41.07; Hedges *g*, 1.08; 95% CI, 0.74-1.47). Similar changes in PHDD were observed among participants who subsequently received diphenhydramine (mean decrease, 27.26; 95% CI, 20.83-33.69; Hedges *g*, 1.02; 95% CI, 0.75-1.44).

**Table 2.** Between- and Within-Group Treatment Effects^a^

	Mean (SD)		Effect		Mean difference (95% CI)	Hedges *g* (95% CI)	*P* value^b^
	Diphenhydramine (n = 45)	Psilocybin (n = 48)					
**% of Heavy drinking days**							
Screening	48.57 (28.73)	56.48 (31.77)	Within-group screening, week 4	Diphenhydramine	27.26 (20.83-33.69)	1.02 (0.75-1.44)	<.001
Week 4^c^	21.31 (20.14)	24.11 (26.29)		Psilocybin	32.37 (23.68-41.07)	1.08 (0.74-1.47)	<.001
Follow-up^d^	23.57 (26.67)	9.71 (26.21)	Between-group follow-up	Diphenhydramine-psilocybin	13.86 (3.00-24.72)	0.52 (0.11-0.94)	.01
**% of Drinking days**							
Screening	71.68 (28.98)	78.03 (27.02)	Within-group screening, week 4	Diphenhydramine	25.68 (19.19-32.18)	0.85 (0.58-1.14)	<.001
Week 4^c^	45.99 (30.40)	52.98 (31.78)		Psilocybin	25.05 (16.92-33.18)	0.83 (0.53-1.16)	<.001
Follow-up^d^	42.83 (33.43)	29.39 (32.86)	Between-group follow-up	Diphenhydramine-psilocybin	13.44 (−0.18 to 27.05)	0.4 (−0.01 to 0.82)	.05
**Drinks per day**							
Screening	4.38 (2.39)	5.2 (2.81)	Within-group screening, week 4	Diphenhydramine	2.19 (1.65-2.73)	0.97 (0.68-1.31)	<.001
Week 4^c^	2.19 (1.98)	2.77 (2.30)		Psilocybin	2.43 (1.87-3.00)	0.91 (0.66-1.23)	<.001
Follow-up^d^	2.26 (2.02)	1.17 (1.99)	Between-group follow-up	Diphenhydramine-psilocybin	1.09 (0.27-1.92)	0.54 (0.13-0.96)	.01

^a^ Positive between-group effect sizes signify lower (more favorable) means in the psilocybin group. Positive within-group effect sizes signify improvement between screening and week 4.

^b^ *P* values for within-group comparisons are based on paired *t* tests with no correction for multiple comparisons. *P* values for between-group comparisons represent univariate marginal between-group contrasts from the primary outcome analysis (multivariate analysis of variance).

^c^ Represents the 4 weeks prior to administration of study medication.

^d^ Represents the 32-week double-blind follow-up period.

### Efficacy

#### Continuous Drinking Outcomes

**Figure 2.**
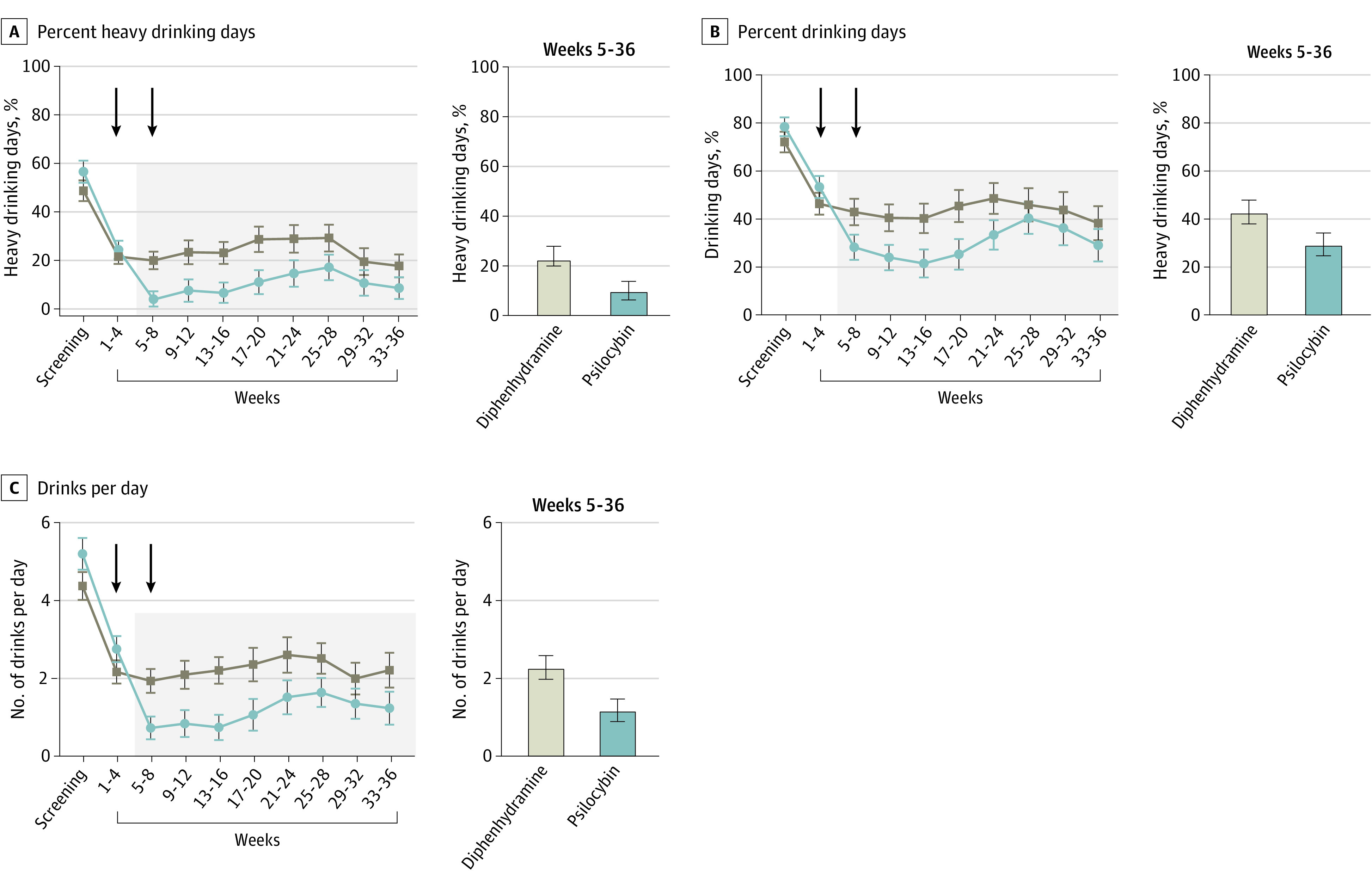
Effects of Treatment on Continuous Drinking Outcomes Mean (SE) estimates for screening (84 days prior to screening), weeks 1-4 (28 days prior to first double-blind medication session; covariate in the model), and eight 28-day bins following the first double-blind medication session (shaded area: weeks 5-8, 9-12, 13-16, 17-20, 21-24, 25-28, 29-32, and 33-36). Arrows represent double-blind medication sessions 1 and 2.

The primary outcome analysis demonstrated a main effect of treatment on the 3-dimensional drinking outcome vector (*F*_1,86_ = 6.18; *P* = .02). During weeks 5 to 36, participants who received psilocybin had lower PHDD than those who received diphenhydramine (mean [SD], 9.71 [26.21] vs 23.57 [26.21]; mean difference, 13.86; 95% CI, 3.00-24.72; Hedges *g*, 0.52; *P* = .01). Results for the secondary continuous drinking outcomes, PDD and DPD, are shown in Table 2. Figure 2 displays estimated monthly means for each of the 3 continuous outcome variables.

#### Dichotomous Drinking Outcomes and Problems Related To Drinking

Participants who were treated with psilocybin were more likely than those receiving diphenhydramine to have no heavy drinking days and to have a 2-level reduction in WHO risk level during weeks 5 to 36 (Table 3). During the final month of follow-up (weeks 33 to 36), these differences persisted, and the rates of abstinence as well as 1- and 3-level reductions in WHO risk levels were also higher in the psilocybin group than in the diphenhydramine group. Numbers needed to treat for these outcomes ranged from 4.0 to 8.2, and odds ratios ranged from 2.03 to 4.74. Participants treated with psilocybin also showed moderate to large reductions in several categories of drinking-related problems at week 24 and/or week 36 (eTable 1 in Supplement 2). Including all available data at the final double-blind time point (week 36), the mean (SD) total problems score was 6.59 (8.80) in those who received psilocybin vs 13.00 (10.48) in those who received diphenhydramine (mean difference, 6.4; 95% CI, 2.22-10.60; Hedges *g*, 0.67; *P* = .003).

**Table 3.** Treatment Effects on Dichotomous Drinking Outcomes

	Follow-up period	No. (%)^a^		NNT	OR (95% CI)^b^	*P* value^b^^,^^c^
		Diphenhydramine (n = 45)	Psilocybin (n = 48)			
Abstinence	Weeks 5-36	4 (8.9)	11 (22.9)	7.1	3.05 (0.89-10.40)	.06
	Weeks 33-36	11 (24.4)	23 (47.9)	4.3	2.84 (1.17-6.89)	.02
No heavy drinking	Weeks 5-36	5 (11.1)	16 (33.3)	4.5	4 (1.32-12.10)	.01
	Weeks 33-36	18 (40.0)	30 (62.5)	4.4	2.5 (1.08-5.76)	.03
**WHO risk level** ^d^						
Decrease 1	Weeks 5-36	32 (71.1)	40 (83.3)	8.2	2.03 (0.75-5.50)	.16
	Weeks 33-36	29 (64.4)	43 (89.6)	4	4.74 (1.57-14.39)	.004
Decrease 2	Weeks 5-36	18 (40.0)	29 (60.4)	4.9	2.29 (1.00-5.26)	.049
	Weeks 33-36	18 (40.0)	29 (60.4)	4.9	2.29 (1.00-5.26)	.049
Decrease 3	Weeks 5-36	6 (13.3)	14 (29.2)	6.3	2.68 (0.93-7.73)	.06
	Weeks 33-36	8 (17.8)	18 (37.5)	5.1	2.78 (1.06-7.26)	.03

Abbreviations: NNT, number needed to treat; OR, odds ratio; WHO, World Health Organization.

^a^ Number and proportion of participants within each treatment group that met dichotomous drinking outcomes for the 32-week double-blind follow-up period following the first medication administration session (weeks 5-36) and the final 4 weeks of double-blind observation (weeks 33-36).

^b^ Confidence intervals and *P* values have not been corrected for multiple comparisons.

^c^ Nominal *P* value, Pearson χ^2^.

^d^ WHO risk levels are defined as follows. Abstinence was defined as no risk (level 0), following a recent study evaluating the use of WHO risk levels as a treatment outcome.^48^ For men, low risk (level 1) is defined as >0 g/d to ≤40 g/d; moderate risk (level 2) as >40 g/d to ≤60 g/d; high risk (level 3) as >60 g/d to ≤100 g/d; and very high risk (level 4) as >100 g/d. For women, low risk (level 1) is defined as >0 g/d to ≤20 g/d; moderate risk (level 2) as >20 g/d to ≤40 g/d; high risk (level 3) as >40 g/d to ≤60 g/d; and very high risk (level 4) as >60 g/d. Change in WHO risk level was calculated in relation to drinking during the 12 weeks prior to screening.

### Safety

A total of 204 adverse events (119 in the psilocybin group and 85 in the diphenhydramine group) were reported during the 32 weeks following the first administration of study medication (eTable 2a in Supplement 2). Three serious adverse events were reported, all in the diphenhydramine group. One participant had 2 psychiatric admissions due to suicidal ideation reported during binge drinking episodes. A second participant was hospitalized for a Mallory-Weiss tear due to severe vomiting during a binge drinking episode.

eTable 2b in Supplement 2 summarizes treatment-emergent adverse events occurring within 48 hours of study drug administration. Headaches were common after psilocybin administration, occurring in 21 of 48 participants who received psilocybin (43.8%) vs 2 of 45 who received diphenhydramine (4.4%). Anxiety and nausea were also reported more frequently during psilocybin administration sessions. Two participants assigned to psilocybin received diazepam, 10 mg, by mouth for anxiety during their second medication session. The anxiety resolved within 45 minutes in one individual and 210 minutes in the other. One participant assigned to psilocybin reported passive suicidal ideation for 15 minutes during a medication session, which resolved without sequelae. There were no persistent disturbances suggestive of psychosis or hallucinogen persisting perception disorder.

## Discussion

In this randomized clinical trial of psilocybin-assisted psychotherapy treatment for AUD, psilocybin treatment was associated with improved drinking outcomes during 32 weeks of double-blind observation. PHDD among participants treated with psilocybin was 41% of that observed in the diphenhydramine-treated group. Exploratory analyses confirmed a between-group effect across a range of secondary drinking measures. Although this was, to our knowledge, the first controlled trial of psilocybin for AUD, these findings are consistent with a meta-analysis^39^ of trials conducted in the 1960s evaluating LSD as a treatment for AUD.

Adverse events associated with psilocybin administration were mostly mild and self-limiting, consistent with other recent trials evaluating the effects of psilocybin in various conditions.^1,2,3,4,5,6,7,8^ However, it must be emphasized that these safety findings cannot be generalized to other contexts. The study implemented measures to ensure safety, including careful medical and psychiatric screening, therapy and monitoring provided by 2 well-trained therapists including a licensed psychiatrist, and the availability of medications to treat acute psychiatric reactions.

### Strengths

This trial had methodological strengths that enhance confidence in these findings. The sample size, although smaller than planned, was the largest of any psilocybin trial yet published to our knowledge. Additional strengths include rigorous assessment and high retention rates over a 32-week period of double-blind follow-up. The psychotherapy used in this trial was manualized and included elements of empirically supported treatments that are commonly used in addiction treatment programs. The effects of psilocybin observed in this trial were over and above the substantial improvement observed in control participants who received the same psychotherapy and reduced their PHDD by more than 50% relative to screening.

### Limitations

Several limitations of the study warrant discussion. First, diphenhydramine was ineffective in maintaining the blind after drug administration, so biased expectancies could have influenced results. Control medications such as methylphenidate,^42^ niacin,^2^ and low-dose psilocybin^1^ likewise did not adequately maintain blinding in past psilocybin trials, so this issue remains a challenge for clinical research on psychedelics. Second, EtG samples, used to validate self-reported drinking outcomes, were available for only 53.8% of treated participants. Third, the study did not have adequate power to evaluate effects in subgroups, such as women, ethnic and racial minority groups, and individuals with psychiatric comorbidity, nor was it designed to identify causal mechanisms, optimal dosing, or predictors of treatment response. Fourth, the study population was lower in drinking intensity at screening than in most AUD medication trials, and results cannot be assumed to generalize to populations with more severe AUD. Fifth, the 2-group design does not permit evaluation of the effects of psychotherapy or the interaction between psychotherapy and medication. Sixth, the study does not provide information on the duration of the effects of psilocybin beyond the 32-week double-blind observation period, which is important given the often chronic, relapsing course of AUD. Further studies will be necessary to address these questions and many others concerning the use of psilocybin in the treatment of AUD.

## Conclusions

In this randomized clinical trial in participants with AUD, psilocybin administered in combination with psychotherapy was associated with robust and sustained decreases in drinking, which were greater than those observed following active placebo with psychotherapy. These results provide support for further study of psilocybin-assisted treatment for adults with AUD.

## Notes

Supplement 1. Trial protocol

Supplement 2. eTable 1. Treatment Effects on Problems Related to Drinking eTable 2. Adverse Events eFigure. Treatment Effects on Cardiovascular Outcomes

Supplement 3. Data sharing statement
