# Supplementary material for: Percentage of Heavy Drinking Days Following Psilocybin-Assisted Psychotherapy vs Placebo in the Treatment of Adult Patients With Alcohol Use Disorder: A Randomized Clinical Trial
Source: JAMA Psychiatry. 2022 Aug 24;79(10):953–62. doi: 10.1001/jamapsychiatry.2022.2096 (PMC9403854; doi:10.1001/jamapsychiatry.2022.2096)
Supplement: Supplement 1. — Trial protocol [file jamapsychiatry-e222096-s001.pdf]

CLINICAL TRIAL PROTOCOL

Protocol # 113080-02

NYU IRB # s14-00614

A DOUBLE-BLIND TRIAL OF PSILOCYBIN-ASSISTED TREATMENT OF ALCOHOL DEPENDENCE

Lead Investigator: Michael P. Bogenschutz, M.D.

Co-Lead Investigator: Stephen Ross, M.D.

Version 4.11

September 1, 2020

Lead Investigator (LI): **Michael P. Bogenschutz, M.D.**
New York University School of Medicine
Department of Psychiatry

Co-Lead Investigator (Co-LI): **Stephen Ross, M.D.**
New York University School of Medicine
Division of Alcoholism & Drug Abuse
Department of Psychiatry

Executive Committee: **Alyssa Forcehimes, Ph.D.**
George Greer, M.D.
John Rotrosen, M.D.
Carey Turnbull

Statistician: **Scott Tonigan, Ph.D.**

Table of Contents

1. PROTOCOL ABSTRACT 1

2. SPECIFIC AIMS 2

3. RESEARCH STRATEGY 3

3.1 SIGNIFICANCE 3

3.1.1 Public Health Impact of Drinking 3

3.1.2 Limitations of Currently Available Treatments for Addiction 3

3.1.3 The Use of Hallucinogens to Treat Addiction 3

3.1.4 Recent Clinical Trials of Psilocybin as Treatment for Various Conditions 4

3.1.5 Safety of Classic Hallucinogens 4

3.1.6 Relationship of Sacramental Hallucinogen Use to Use of Other Substances 4

3.1.7 How Might a Classic Hallucinogen Affect Drinking Behavior? 5

3.1.8 Neuroimaging Studies of Acute Effects of Classic Hallucinogens Human Brain Function 5

3.2 INNOVATION 7

3.3 APPROACH 7

3.3.1 Design Overview 7

3.3.2 Primary Outcome 9

3.3.3 Preliminary Studies 9

3.3.4 Recruitment, Pre-screening, and Informed Consent 11

3.3.5 Randomization and Blinding 11

3.3.6 Study Participants 12

3.3.7 Sites 13

3.3.8 Study Treatments 13

3.3.9 Concomitant Therapy 19

3.3.10 Measures 20

3.3.11 Data Management and Procedures 24

3.3.12 Statistical analysis and power 25

3.3.13 Study Milestones 27

3.3.14 Regulatory Considerations 28

3.3.15 Compensation to Participants 29

4. HUMAN SUBJECTS PROTECTIONS 29

4.1 RISKS TO HUMAN SUBJECTS 29

4.1.1 Human Subjects Involvement and Characteristics. 29

4.1.2 Sources of Materials 30

4.1.3 Potential Risks 30

4.2 ADEQUACY OF PROTECTION AGAINST RISKS 33

4.2.1 Recruitment and Informed Consent 33

4.2.2 Protection Against Risk 33

4.3 Potential Benefits of the Proposed Research to Human Subjects and Others 35
4.3.1 Potential Benefits to Participants *35*
4.3.2 Potential Benefits to Others. *35*
4.3.3 Risk Benefit Assessment *35*
4.4 Importance of the Knowledge to be Gained 35
4.5 Data and Safety Monitoring Plan 35
4.5.1 General Considerations *35*
4.5.2 Institutional Review Board (IRB) *35*
4.5.3 Data and Safety Monitoring Board *35*
4.5.4 Procedures for Monitoring and Reporting Adverse Events. *36*
5. REFERENCES 37

1. PROTOCOL ABSTRACT

Several lines of evidence suggest that classic hallucinogens such as psilocybin can facilitate behavior change in addictions such as alcohol dependence. The proposed investigation is a multi-site, double-blind active-controlled trial (n = 180, 90 per group) contrasting the acute and persisting effects of psilocybin to those of diphenhydramine in the context of outpatient alcoholism treatment. Two to four sites will participate. Aims of the study are 1) to characterize the acute effects of PO psilocybin 25 mg/70 kg, 30 mg/70 kg, and 40 mg/70 kg in alcohol dependent patients; 2) to evaluate the effect of psilocybin treatment on drinking outcomes for 32 weeks after the first administration, relative to diphenhydramine control; 3) to test whether or not characteristics of the drug administration session experiences mediate effects of psilocybin on short-term (1 week) persisting effects and post-session drinking behavior, 4) to evaluate the explanatory value of changes in alcohol craving, self-efficacy, motivation, and other psychological domains in accounting for the observed experimental effect of psilocybin relative to diphenhydramine control and 5) to evaluate pre-post changes in drinking in participants after they receive psilocybin in the third session.

The total duration of psychosocial treatment in the double-blind period will be 12 weeks, and double-blind drug administration sessions will occur after 4 and 8 weeks. In the first psilocybin session, a dose of 25 mg/70 kg will be administered. Depending on the response in the first session, the dose for the second session may be increased to 30 mg/70 kg or 40 mg/70 kg, or held at 25mg/70kg. The dose of diphenhydramine will start at 50 mg, and may be increased to 100 mg or held at 50 mg in the second session, depending on response in the first session. Following completion of the double-blind period (34 weeks after randomization) all participants who meet interim safety criteria will be offered an additional session in which psilocybin will be administered. The drug will be administered during 8-hour sessions in an outpatient setting under close medical and psychiatric monitoring. The drug administration sessions will occur in the context of an extended version of Motivational Enhancement Therapy (Motivational Enhancement and Taking Action, META) with the addition of standardized preparation before and debriefing and follow-up after the psilocybin administration sessions. Extensive screening and baseline assessment will be completed, including thorough safety screening and assessment of participant characteristics that could potentially moderate treatment response. Within-session and short-term persisting effects will be assessed. Drinking outcomes and changes in several potential mediators of treatment effect, including motivation, self-efficacy, craving, depression, anxiety, and spiritual dimensions of the experience, will be measured until 50 weeks after the first drug administration session, for a total of 54 weeks from the initiation of treatment.

2. SPECIFIC AIMS

Several lines of evidence suggest that classic (5HT2a agonist or partial agonist) hallucinogens such as psilocybin can promote behavior change in addictions such as alcohol dependence. In particular, a recent meta-analysis of the most rigorous randomized controlled trials of LSD for alcohol dependence (all conducted over 40 years ago) demonstrated a consistent medium-sized effect (in terms of reduced alcohol use/clinical improvement from alcoholism) of a single high-dose session [1]. The goal of the proposed line of research is to use modern clinical trial methodology and evidence-based behavioral treatment to address seriously an important question that was never answered satisfactorily: can administration of a classic hallucinogen, under carefully controlled conditions and integrated with a high-quality, evidence-based behavioral treatment, improve alcoholism treatment outcomes above those of the behavioral treatment without the hallucinogen?

Building on our open-label pilot study [2, 3], we propose a multi-site, double-blind, active-controlled trial (n = 180) designed to assess the effects of psilocybin when administered orally to alcohol dependent participants receiving evidence-based psychosocial treatment as well as preparation and debriefing sessions that provide a supportive context for the psilocybin experience. The duration of psychosocial treatment in the double blind period will be 12 weeks, and double-blind drug-administration sessions will occur after 4 and 8 weeks. Participants will be randomly assigned to psilocybin or diphenhydramine (n = 90 per group) in two double-blind sessions. The first psilocybin session will employ a dose of psilocybin 25 mg/70 kg. The dose may be increased in the second session to 30 mg/70 kg or 40 mg/70kg based on response in the first session. The dose of diphenhydramine will start at 50 mg, and may be increased to 100 mg in the second session. Following completion of the double-blind period (34 weeks after randomization) all participants who meet interim safety criteria will be offered an additional session in which psilocybin will be administered, and participants will be followed for an additional 16 weeks. Drinking outcomes and changes in several potential mediators of treatment effect, including motivation, self-efficacy, craving, depression, anxiety, and spiritual dimensions of the experience, will be measured until 50 weeks after the first psilocybin session, for a total of 54 weeks.

Aim 1: To characterize the acute effects of PO psilocybin 25 mg/70 kg, 30 mg/70 kg, and 40 mg/70 kg in alcohol dependent patients: Hypothesis 1: The drug will be well-tolerated, and acute effects will be similar to those observed in normal volunteers.

Aim 2: To evaluate the effect of psilocybin treatment on drinking outcomes for 32 weeks after the first administration, relative to diphenhydramine control. Hypothesis 2: A greater decrease in percent heavy drinking days will be observed among participants receiving psychoactive doses of psilocybin in combination with psychosocial treatment than in those receiving diphenhydramine.

Aim 3: To test whether or not characteristics of the drug administration session experiences mediate effects of psilocybin on short term (1 week) persisting effects and post-session drinking behavior. Hypothesis 3: Intensity of experience during the drug administration sessions will predict post-session changes in craving, self-efficacy, and motivation, and these changes will in turn predict changes in drinking behavior during the 32 weeks following initial drug administration.

Aim 4: To evaluate the explanatory value of changes in alcohol craving, self-efficacy, motivation, and other psychological domains in accounting for the observed experimental effect of psilocybin relative to diphenhydramine control. Hypothesis 4: Changes in craving, self-efficacy, and motivation will mediate the effects of psilocybin treatment on drinking behavior.

Aim 5: To evaluate pre-post changes in drinking in participants after they receive psilocybin in the third session. Hypothesis 5: participants in the control group will show decreases in drinking during the 16 weeks following the psilocybin session at week 38.

3. RESEARCH STRATEGY

3.1 Significance

3.1.1 Public Health Impact of Drinking

Of all drugs of abuse, alcohol is possibly the most damaging in the US and globally when factoring in preventable death, premature death, disability, healthcare/societal costs, adverse medical and neuropsychiatric complications, unintentional injuries, and its significant causal link to suicidal and violent behaviors. Alcohol use disorders (AUDs) are highly prevalent in the US, affecting up to 12% of the population at some point in their lifetimes [4]. AUDs are among the most disabling of all diseases worldwide, and alcohol use is responsible for approximately 4.6% of global disability-adjusted life-years and 36.4% of neuropsychiatric disability-adjusted life-years in 2004 [5]. In the US alcohol accounts for 12.1% of disability-adjusted life-years in men, and 4.6% in women [5]. In attempting to rank the relative harm of drugs of abuse, Nutt and colleagues created a relative ranking scale of harm from drugs of abuse in the UK, (looking at harmful effects both to the individual user and community), alcohol ranked first out of all drugs of abuse, ahead of other notoriously harmful drugs such as crack cocaine and heroin. With scores of 0–100 (no harm to maximum harm), alcohol scored 72 compared to heroin (55, second) and crack cocaine (54, third), with Psilocybe mushrooms coming in last (6), rated as the least harmful drug to society [6]. The economic cost of alcohol use is staggering, estimated at $185 billion per annum in the US alone, vs. $158 billion for nicotine and $280 billion for illicit drugs (higher because of costs related to criminalization) [7].

3.1.2 Limitations of Currently Available Treatments for Addiction

Addictions exemplify the resistance of maladaptive behaviors to interventions aimed at bringing about change. Substantial progress has been made in understanding the neurobiological and neuropsychological underpinnings of the loss of control that is the hallmark of addiction. A number of pharmacological and behavioral treatments have been developed that target specific aspects of addiction, including motivation, coping skills, social support, reward/punishment, and relapse due to stress, priming doses of the drug, or exposure to conditioned cues. However, the effects of currently available treatments remain disappointingly small, particularly for pharmacotherapies other than agonist medications such as methadone [8]. Although combining effective treatments (either multiple pharmacotherapies or pharmacotherapy plus behavioral therapy) is an obvious strategy to increase treatment effect sizes, attempts to improve outcomes by combination therapy have been largely disappointing [9, 10].

3.1.3 The Use of Hallucinogens to Treat Addiction

In the 1950s through early 1970s there was extensive research on the use of the prototypical classic hallucinogen LSD in the treatment of alcoholism (For reviews see [11-15]), with more limited research on LSD for the treatment of drug addiction [16, 17] and dipropyltryptamine (DPT) for alcohol dependence [18, 19]. At least a dozen trials included some form of control group [1, 20]. A recent meta-analysis [1] examined 6 randomized trials (4 of which were fully double-blind) of LSD for alcohol dependence [21-26]. A total of 325 participants received active treatment with LSD, and 211 received control treatment. These studies all employed a single high-dose LSD session, and the vast majority of participants were male inpatient alcoholics. The studies were otherwise quite heterogeneous, with sample sizes varying from 20 to 176, LSD doses ranging from about 210 to 800 mcg, control conditions including placebo, low dose (25-50 mcg) LSD, ephedrine, and amphetamine, and great variability in preparation and debriefing of subjects and in the conditions during the LSD sessions. At the first post-treatment follow-up (ranging from 1 month to 12 months) the odds ratio for improvement was 1.96, favoring LSD (95% confidence interval 1.36-2.84, $Z = 3.59$, $p = .0003$). Among the 5 studies reporting dichotomous outcomes, 59% of the LSD-treated participants were significantly improved, vs. 38% of the control participants (pooled benefit difference 16%, 95% confidence interval 8%-25%, $p = .0003$, number needed to treat = 6). Treatment effects decreased with the duration of follow-up, but remained significant at 6 months. These effects were highly consistent across the 6 studies. These robust effects provide a strong rationale for resuming clinical investigation of classic hallucinogens for the treatment of alcoholism and other addictions. With few exceptions, clinical research on hallucinogens was discontinued in the early 1970s, after enactment of the Controlled Substances Act placed all such compounds into the highly restrictive Schedule I class.

Ibogaine, a plant-derived hallucinogen with a complex pharmacological profile, later attracted interest when heroin addicts reported that they experienced a significantly attenuated withdrawal syndrome and subsequently lost the desire to use heroin after taking ibogaine. Although initial preclinical and clinical results in the treatment of opioid withdrawal were encouraging, this line of research was eventually discontinued due to toxicity concerns [27].

Finally, ketamine has been investigated in Russia as a potential treatment for both alcohol dependence and heroin dependence. Although ketamine is an NMDA receptor antagonist rather than a serotonergic hallucinogen, recent research reveals considerable overlap in the brain effects of these two classes of drugs [28]. In a controlled but non-randomized study, alcohol dependent subjects who volunteered to receive ketamine-assisted psychotherapy (ketamine 2.5 mg/kg IM, a dose producing prominent hallucinogen effects) showed significantly higher rates of abstinence at 1 year (73/111 = 65.8%) than those receiving usual care in the same facility (24/100 = 24%) [29]. In a randomized, double-blind trial (n = 70), heroin-dependent patients assigned to a single session of ketamine 2 mg/kg IM had significantly higher abstinence rates over 24 months of follow-up than those who received a lower, mildly psychoactive dose [30]. Both groups showed significant decreases in depression and anxiety which persisted for at least 6 months, but participants in the high-dose condition demonstrated greater and more enduring reductions in heroin craving. In a second study (n = 53), patients receiving 2 or 3 ketamine sessions had higher abstinence rates over 1 year of follow-up (50% continuous abstinence at 1 year) than those who received 1 session (22% continuous abstinence) [31]. Off-label use of ketamine for alcohol dependence has been reported in the US, but quantitative outcome data are not available [32]. Ketamine also has been shown to produce rapid and robust antidepressant effects in patients with treatment-resistant major depression and bipolar depression [33-39].

3.1.4 Recent Clinical Trials of Psilocybin as Treatment for Various Conditions

The past decade has seen a resurgence of interest in potential clinical applications of classic hallucinogens, particularly psilocybin. The effect of varying doses of psilocybin (doses up to 0.3 mg/kg PO) on symptoms of obsessive compulsive disorder was tested in 9 subjects in a within-subjects design [40]. All doses tested produced significant decreases in OCD symptomatology, but there was no effect of dose or dose-by-time interaction. Using a double-blind, cross-over design, Grob et al. administered psilocybin 0.2 mg/kg vs. placebo to 12 patients with anxiety related to advanced cancer [41]. Although significant treatment effects were not demonstrated in this pilot study, there were statistical trends suggesting a positive effect of psilocybin on mood. The low dose used may have limited efficacy in this study. Additional clinical trials are currently under way in cancer patients. Currently, psilocybin is being investigated as an adjunct in smoking cessation treatment in an open-label pilot study at Johns Hopkins University [42].

3.1.5 Safety of Classic Hallucinogens

Extensive clinical research with the classic hallucinogens (LSD, psilocybin, DMT, mescaline) has established their relative safety within a clinical research setting when subjects are carefully screened, supervised, and followed up [43]. Although classic hallucinogens present significant dangers if misused or abused, they do not cause physical dependence, and full-blown psychological dependence appears to be relatively infrequent among users [44, 45]. Psilocybin, like other classic hallucinogens, is not reliably reinforcing in humans or in animal models used to study addiction potential [46]. Hallucinogen persisting perception disorder, though poorly understood, appears to be uncommon, even less so in research than illicit use settings, and more common following LSD use than use of other hallucinogens [47] (See Section 4.1.3 for discussion of risks and Section 4.2.2 for protections against risks).

3.1.6 Relationship of Sacramental Hallucinogen Use to Use of Other Substances

Cross-sectional studies have consistently shown decreased rates of alcohol dependence among members of religions that use classic hallucinogens as a regular part of their practice, including the Native American Church, which uses the mescaline-containing peyote cactus as a sacrament [48], and both Brazilian [49, 50] and US [51] sects using ayahuasca (a botanical preparation which contains DMT and monoamine oxidase inhibitors that render DMT orally active). Although cultural norms within these religions likely contribute to such effects, the pattern suggests the possibility of a pharmacological effect as well.

3.1.7 How Might a Classic Hallucinogen Affect Drinking Behavior?

A number of articles and chapters have reviewed the literature on the use of hallucinogens in the treatment of addictions [11-15], with the recent addition of two reviews that incorporate current research on the effects of classic hallucinogens more generally and discuss possible mechanisms of action [52, 53]. Here we provide a brief summary of the most important lines of evidence.

3.1.7.1 Relevant Preclinical Research

Although classic hallucinogens bind to many serotonin receptor subtypes and other receptors including D1 and D3 receptors [54], the psychoactive effects of all classic hallucinogens appear to depend primarily on their actions at 5HT2A receptors [28, 55]. Ketanserin, a 5HT2A antagonist, blocks most of the subjective effects of psilocybin in humans [56]. Stimulation of 5HT2A receptors by 5HT2A agonists causes activation of sub-populations of pyramidal cells in cerebral cortex by enhancing glutamatergic neurotransmission within intracortical networks, particularly those involving cortical layer V [57-60]. In mouse models, effects of hallucinogenic 5HT2A agonists are mediated by different intracellular signaling cascades from those activated by serotonin and non-hallucinogenic 5HT2A agonists [61-63]. The effects of LSD are mediated in part by pathways involving pertussis toxin-sensitive Gi/o proteins and Src, while the effects of lisuride (a non-hallucinogenic 5HT2A agonist) do not depend on these pathways [61]. Moreno and colleagues demonstrated that the metabotropic glutamate mGlu2 receptor, which forms complexes with 5HT2A receptors, is necessary for the pharmacological and behavioral effects of hallucinogenic 5-HT2A agonists [64].

Very little is known about persisting brain changes related to use of classic hallucinogens in treatment of addiction, but there are animal data suggesting directions for future research. Administration of classic hallucinogens in rat models has been shown to induce down-regulation of 5HT2A receptors, particularly those in the anterior cingulate and frontomedial cortex, likely accounting for the rapid development and reversal of behavioral tolerance to most classic hallucinogens [65, 66]. This is interesting in relation to evidence that 5HT2A receptors are upregulated with chronic alcohol exposure [67]. DOI and serotonin increase expression of glial cell line-derived neurotrophic factor (GDNF) mRNA in glioiblastoma cells by a 5HT2A-dependent mechanism [68]. Through its action on 5HT2A receptors, DOI has also been shown to increase levels of mRNA for brain-derived neurotrophic factor (BDNF) in rat parietal cortex and other neocortical regions, with decreases in the hippocampus and no change in piriform cortex [69]. These findings are relevant because levels of BDNF and GDNF are inversely related to alcohol consumption and conditioned place preference in animal models [70]. DOI activates intracellular signaling cascades associated with dendritic spine remodeling on rat pyramidal cells, and transiently increases the size of dendritic spines on cortical neurons [71]. At present there is no direct evidence as to whether any of these mechanisms mediates antiaddictive effects of classic hallucinogens.

3.1.8 Neuroimaging Studies of Acute Effects of Classic Hallucinogens Human Brain Function

PET, SPECT, and fMRI technologies have been applied to studies of the acute effects of classic hallucinogens, specifically psilocybin and ayahuasca (an herbal decoction containing dimethyltryptamine (DMT) and beta-carbolines including harmine, harmaline, and tetrahydroharmine). The beta-carbolines are monoamine oxidase inhibitors that render DMT orally active, as well as having psychoactive effects themselves). In a PET study of effects of psilocybin (15-20 mg PO), Vollenweider and colleagues found that global cerebral glucose metabolism increased by 19.9% relative to baseline, with the largest increases in thalamus and frontomedial, frontolateral, anterior cingulate, and temporomedial cortices [72]. In a placebo-controlled study by the same group, psilocybin 0.2 mg/kg produced increased glucose metabolism in the right anterior cingulate and right frontal operculum, with decreased metabolism in the right thalamus and left precentral cortex [73]. A placebo-controlled SPECT study of ayahuasca administration by Riba and colleagues demonstrated increased blood flow in the anterior insula bilaterally, in right anterior cingulate/frontomedial cortex, and in the left amygdala/parahippocampal gyrus [74]. In contrast, a recent fMRI study from Carhart-Harris and colleagues using psilocybin 2mg administered intravenously, found that psilocybin administration was associated with decreases in regional cerebral blood flow and BOLD signal, with strongest effects in anterior cingulate cortex/medial prefrontal cortex, posterior cingulate cortex, and thalamus [75]. Psilocybin also decreased functional coupling between medial prefrontal cortex and posterior cingulate cortex. A secondary analysis of data from this study showed that during the acute effects of psilocybin there was increased

functional connectivity between two important brain networks: the default mode network, normally activated during internally oriented thinking, and the task-positive network, normally activated when attention is oriented toward external events or activities [76]. This could provide a biological explanation for the blurring of self-other boundaries that is often a prominent feature of hallucinogen effects. Another report from this group demonstrated that intravenous psilocybin increased activation in multiple brain regions in response to recollection of autobiographical events [77]. Clearly we are only beginning to understand the acute effects of classic hallucinogens on brain function. In addition, there have been no neuroimaging studies of the persisting effects of classic hallucinogens. Since effects of some sort must persist beyond the period of acute intoxication for the treatment to have any clinical value, persisting brain effects are more directly relevant to therapeutic benefit than are acute brain effects.

3.1.8.1 Can Administration of Classic Hallucinogens Precipitate or Facilitate Lasting Psychological Change?

Although alcoholism is a disorder which frequently has a chronic or relapsing course [78], rapid, profound, and lasting behavior change can and does occur. Over one hundred years ago William James described the phenomenon of sudden transformative change of a religious or spiritual nature [79], which was often accompanied by changes in behavior such as the abrupt onset of sobriety in alcoholics. More recently, William R. Miller and others have elucidated the nature of what has been termed “quantum change” [80, 81]. Sudden and lasting behavior change can be triggered by an acute transformative experience that is frequently but not always described as spiritual in nature. Such experiences figure prominently in the AA literature [82]. In a large (n = 587) alcoholism treatment sample, reporting a recent “spiritual awakening” was associated with markedly increased rates of 12-month continuous abstinence (odds ration = 3.9) [83]. These experiences are not uncommon in the general population or among alcoholics [84]. However, there has never been a reliable way to elicit and apply such experiences to mobilize predictable change in a particular behavioral disorder such as alcohol dependence.

Classic hallucinogens (primarily LSD) were studied extensively from the 1950s through the early 1970s in the treatment of alcohol and drug addiction as well as anxiety, depression, obsessive-compulsive disorder, and other conditions. Two contrasting models of treatment developed: the psycholytic and psychedelic models [15, 85]. In psycholytic therapy, low to moderate doses of hallucinogens were administered, usually on many occasions over a period of months to years, to facilitate therapy based on traditional psychoanalytic principles [86, 87]. The psychedelic method used higher doses of LSD, administered once or on a few occasions, with the goal of inducing a “peak-psychedelic” experience [88, 89]. The psychedelic model held that such experiences often produced lasting change in habitual patterns of thought, emotional response, and behavior. Many of the studies from the 1960s, particularly those employing the psychedelic model, reported changes on measures of personality following hallucinogen administration [90-94].

More recently, rigorous quantitative studies have demonstrated that psilocybin can occasion profoundly meaningful experiences that have significant lasting effects in normal volunteers [95-98]. In a double-blind study by Griffiths and colleagues, 22 out of 36 participants receiving a single high-dose psilocybin session met a priori criteria for a “complete mystical experience” (score of 0.6 or greater on 6 subscales of the Pahnke-Richards Mystical Experience Questionnaire [97]). Fourteen months after the psilocybin session, 67% of participants rated it as one of the 5 most significant spiritual experiences of their lives, and 61% reported that the experience was associated with “moderate to extreme positive behavioral change,” as well as positive changes in attitudes, mood, and altruism [96]. These self-reports correlated with ratings by community observers who reported similar positive changes in participants. In a second study in which participants received a range of doses of psilocybin, 72% of volunteers reported a “complete mystical experience” at one of the highest two doses [98]. Persisting positive effects one month after the psilocybin session were found to be dose-related.

Based on these data, we have proposed a number of possible causal pathways that could lead to therapeutic effects of classic hallucinogens in the treatment of addiction (See Figure 1) [99]. Briefly, the effects of classic hallucinogens are expected to be highly dependent on environmental and subject factors (the "set and setting hypothesis"). Acute brain effects may be measured at the level of the receptor, neuron, functional network, or patient-reported subjective experience. Persisting effects likewise may be measured in terms of structural and functional brain changes as well as in psychological terms. Ultimately, to affect drinking behavior, this causal chain would need to lead to effects that directly affect drinking, such as decreased craving or enhanced self-efficacy or motivation. Is must be stressed that the specific mediators listed here are hypothesized based on what is known about the effects of classic hallucinogens and change processes in addiction, but have not been directly investigated in addiction treatment trials. The proposed study will examine the effects of psilocybin administration on several of these possible mediating variables.

Figure 1

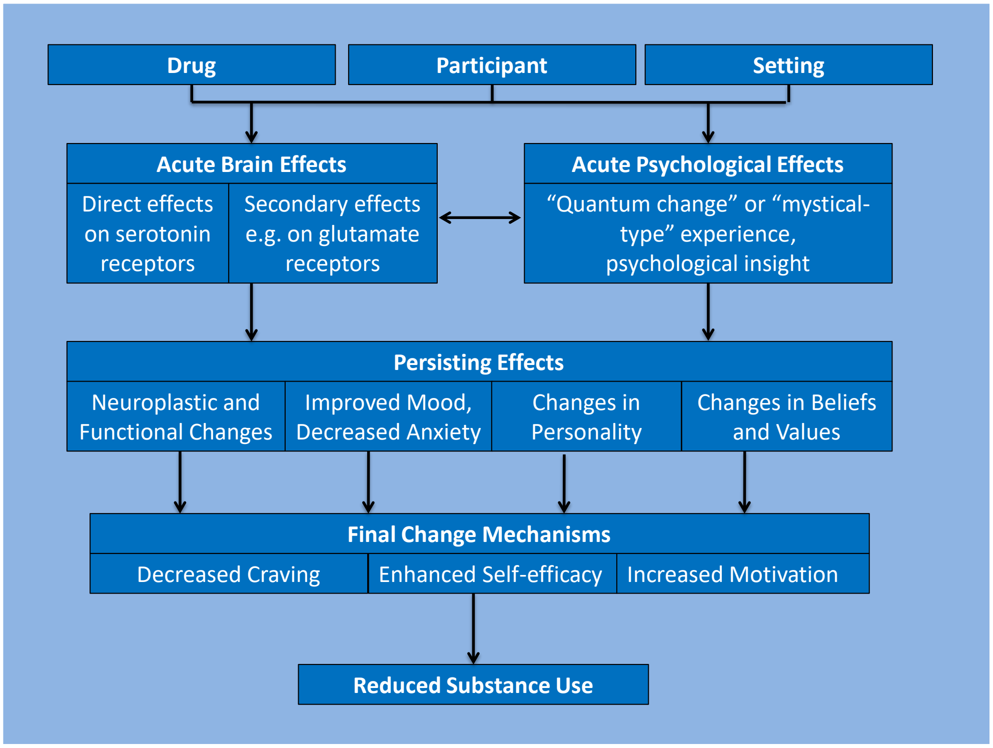

3.2 Innovation

The proposed study brings together experienced researchers at the nexus of their areas of expertise to conduct a study that is highly innovative in several respects. The evidence summarized above, coming from several sources, provides a convincing rationale for reopening the question of whether a classic hallucinogen can improve treatment response among patients with alcohol dependence. Although this is not a novel question, it has not been investigated for 4 decades. In spite of the accumulating data indicating that psilocybin has clinically relevant effects and is relatively safe under controlled conditions, our pilot study is the only trial that has ever has been conducted using psilocybin in the treatment of alcohol dependence. There have been no recent controlled trials using any drug in this class in the treatment of addiction. The proposed study is highly innovative because classic hallucinogens have effects and mechanisms of action that are entirely distinct from any medications that are currently used in treatment of addiction. This study will be the first to use modern clinical trials technology to study the effects of a classic hallucinogen in alcohol dependent subjects, and the first controlled trial to use an established evidence-based behavioral therapy in combination with hallucinogen administration in the treatment of addiction. This study will also be the first controlled trial to investigate whether putative change mechanisms are mobilized by administration of psilocybin to alcohol dependent patients. More broadly, the administration of hallucinogens provides an attractive experimental model to study the phenomenon of quantum change, in which a single transformative experience can produce lasting and meaningful behavior change.

3.3 Approach

3.3.1 Design Overview

The proposed investigation is a multi-site (2-4 sites), double-blind, active-controlled trial (n = 180, 90 per group) contrasting the acute and persisting effects of psilocybin to those of diphenhydramine in the context of outpatient alcoholism treatment. During the double-blind phase, participants will receive psilocybin or diphenhydramine in two monthly sessions, with dose for the second session titrated base on response in the first session. Following completion of the double-blind phase of the protocol (at week 38, 34 weeks after randomization) all participants who meet interim safety criteria will be offered an additional session in which psilocybin will be administered, and participants will be followed for an additional 16 weeks. The drug will be administered during 8 hour sessions in an outpatient setting under close medical and psychiatric monitoring. The drug administration sessions will occur in the context of a behavioral intervention consisting of an extended version of Motivational Enhancement Therapy, (Motivational Enhancement Therapy and Taking Action, META), with the addition of standardized preparation before and debriefing and follow-up after the psilocybin administration sessions. Extensive screening and baseline assessment will be completed, including thorough safety screening and assessment of participant characteristics that could potentially moderate treatment response. Within-session and short-term persisting effects will be assessed. Drinking outcomes and changes in several potential mediators of treatment effect, including motivation, self-efficacy, craving, depression, anxiety, and spiritual dimensions of the experience, will be measured for 50 weeks following initial administration of study medication (See Figure 2).

Figure 2: Overview of study design

Study period	Week	Treatment			Assessment
Period 1	-4 to -1				Screening
Pre-treatment	-1 to 0				Baseline
Period 2	0		1 (META)		
Pre-Randomization	1		2 (META)		
	2		3 (PSI)		
	3		4 (PSI)		
	4	Psilocybin 25mg/70kg	5 (PSI)	Diphenhydramine 50 mg	4 weeks
	4.1		6 (PSI)		
	5		7 (META)		5 weeks
Period 3	6		8 (META)		
Double-blind	7		9 (PSI)		
Phase	8	Psilocybin 25-40mg/70kg*	10 (PS1)	Diphenhydramine 50-100 mg*	8 weeks
	8.1		11 (PSI)		
	9		12 (META)		9 weeks
	10		13 (META)		
	12		14 (META/PSI)		12 weeks
	24				24 weeks

	**36**				**36 weeks**
	**37**		**15 (PSI)**		
**Period 4 Open-label**	**38**	**Psilocybin 25-40mg/70kg***	**16 (PSI)**	**Psilocybin 25mg/70kg**	**38 weeks**
**Extension phase**	**38.1**		**17 (PSI)**		
	**39**		**18 (META/PSI)**		**39 weeks**
	**42**		**19 (META/PSI)**		**42 weeks**
	**54**				**54 weeks**

***Dose titrated based on response in prior session, see section 3.3.8.1.3**

3.3.2 Primary Outcome

The primary outcome for the study, used to test hypothesis 2, is percent heavy drinking days as assessed by the Time-line Follow-back [100], during the 32 weeks following the first drug administration session. Secondary drinking outcomes will include percent days abstinent, drinks per drinking day, days to first drinking day, days to first heavy drinking day, and consequences of drinking (from the Short Inventory of Problems [101]).

3.3.3 Preliminary Studies

The UNM team has conducted an open-label pilot study of psilocybin for alcohol dependence, which is now nearing completion. In the pilot study, participants receive two psilocybin sessions at 4 weeks and 8 weeks in the context of 12 weeks of psychosocial treatment. The first dose of psilocybin is 0.3 mg/kg, and the second dose is 0.3 mg/kg or 0.4 mg/kg, depending on the effects of the first session. Participants are assessed at baseline, 4 weeks (prior to first drug administration) 5 weeks (1 week after drug administration), 8 weeks (prior to second drug administration), 12 weeks, 24 weeks, and 36 weeks.

3.3.3.1 Behavioral Interventions Used in UNM Pilot Study

Behavioral interventions for alcoholism treatment require adaptation for use in combination with classic hallucinogens with the following issues in mind. It is crucial to provide a supportive context for hallucinogen administration sessions, including preparation before and debriefing after psilocybin administration. Proper preparation for a hallucinogen session is critical to establish a therapeutic “set” and to minimize the likelihood of adverse psychological reactions. The debriefing process is intended to help the participant integrate and process the experience, and to assess for the presence of any adverse reactions that may require attention. The ideal behavioral treatment for use in a trial of a hallucinogen administration protocol would be broadly effective and have the potential to work synergistically with the hypothesized mechanisms of action of the hallucinogen. The length of the behavioral treatment should be appropriate to the duration of the pharmacotherapy.

Based on these considerations, we developed a manualized 12-session behavioral intervention for use together with the two psilocybin administration sessions in the pilot study. The behavioral intervention comprised 7 sessions of Motivational Enhancement Therapy (MET: a structured approach using the principles of Motivational Interviewing [102]), including 4 standard MET sessions and 3 booster sessions; 3 preparation sessions; and 2 debriefing sessions (The MET sessions also provided some opportunity for debriefing). The MET content was based on procedures used previously in multisite trials [103, 104]. The second MET session provided an opportunity to focus specifically on the participant’s values (including spiritual values) and the discrepancy between values and behavior as motivation for change. Subsequent sessions followed up on the participant’s goals and change plan in relation to these values. The preparation and debriefing procedures were based on those of the Johns Hopkins group [105] and those used in other prior hallucinogen administration studies [106-108]. Preparation and debriefing sessions were conducted by a study physician together with the MET therapist, while the MET sessions were normally conducted by the MET therapist alone.

Overall, this therapy model appeared to work well in the context of a 12-week, 2-session treatment model. The preparation and debriefing procedures appeared to be effective. The MET seemed to function well to engage patients in treatment, to enhance their motivation for change prior to the first psilocybin session, and to elicit reasons for change and initial change plans. However, therapists reported that most participants were ready to take active steps toward change after the first psilocybin session, and felt that they would have benefitted from more action-oriented treatment.

3.3.3.2 Pilot Study Results

Figure 3 illustrates available pilot results by individual through week 8. Participants were 6 men and 4 women, mean age 40.1 (S.D. 10.3), who were concerned about their drinking but not interested in available treatment. Participants had been alcohol dependent for a mean of 15.1 years (S.D. 11.5) and ranged in severity from mild to severe dependence with a mean of 5.0 dependence criteria (S.D. 1.2).

Participants have tolerated psilocybin well in the pilot study. Transient mild headaches have been common during the 24 hours following psilocybin administration, as reported by Johnson et al. [109]. There have been no persisting adverse events that were considered related to the study treatments. Figure 3a shows mean scores on the Mystical Experience Questionnaire (MEQ) total score for the 10 participants receiving 0.3 mg/kg in the first session and 5 participants receiving 0.4 mg/kg in the second session, as well as scores reported at similar doses in normal volunteers by Griffiths et al. [110]. On average, acute effects are lower in magnitude than those seen at comparable doses in normal volunteers. Intensity of effects varied markedly from patient to patient, with MEQ scores ranging from .02 to .77 in the first session, and Hallucinogen Rating Scale (HRS) intensity scores ranging from 0 to 3.5. The mean score for the 0.4 mg/kg dose appears to be lower than that at 0.3 mg/kg. This is probably because the four participants with the strongest response in the first session did not receive 0.4 mg/kg in the second session.

Results (n=9) for the month before and the two months after the first psilocybin session are shown in Figures 3b-3d. Participants showed modest change in drinking behavior during the first month of psychosocial treatment. However, drinking days and heavy drinking days decreased significantly during the month following the first psilocybin session. Changes in drinking were accompanied by modest significant increases in self-efficacy (AASE confidence scale) following the psilocybin session. Importantly, the magnitude of the effects reported during the first psilocybin session (at week 4) is strongly correlated with changes in craving and self-efficacy in the week following the session, and with changes in drinking in the month following the session. In addition, changes in craving and self-efficacy at week 5 are correlated with changes in drinking during weeks 5-8 relative to weeks 1-4.

Figure 3a. MEQ Total Score

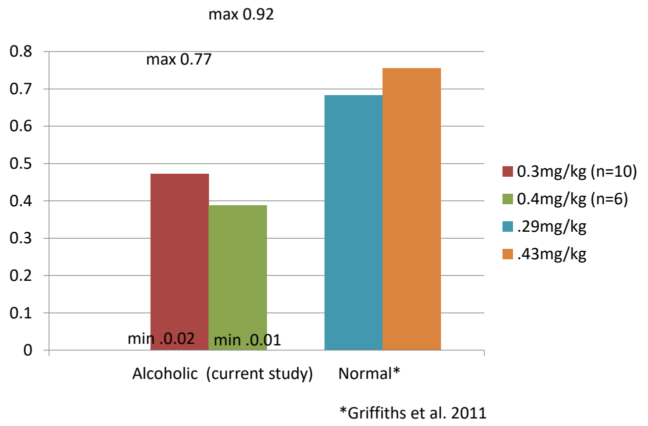

Figure 3b. Percent drinking days and Percent heavy drinking days

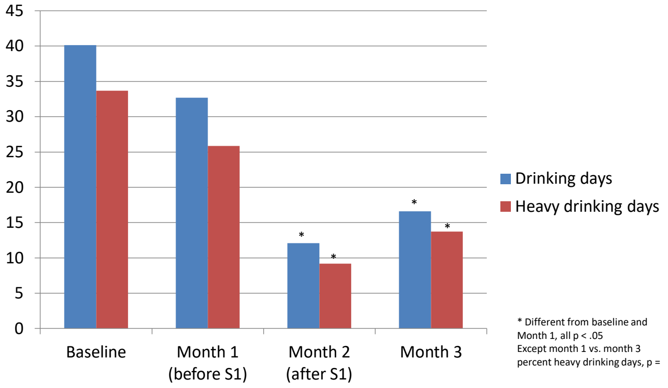

Figure 3c. Self-Efficacy (AASE Confidence)

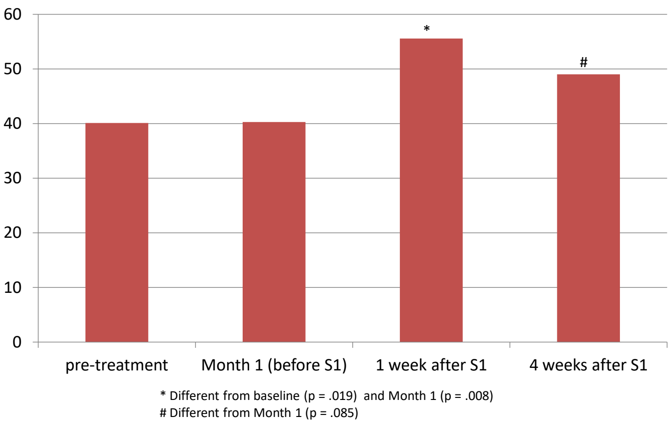

Figure 3d. Correlations with intensity of 1st session

Pearson's r (n = 9)	Change in PACS (wk 5-wk 4)	Change in AASE conf. (wk 5-wk 4)	Change in PDD (wk 8-wk 4)	Change in PHDD (wk 8-wk 4)
MEQ total (wk 4)	r = -.810 p = .008	r = .762 p = .017	r =-.884 p = .002	r = -.823 p = .006
HRS Intensity Scale (wk 4)	r = -.823 p = .006	r = .753 p = .019	r = -.834 p = .005	r = -.762 p = .017
HRS item 98 (wk 4)	r = -.862 p = .003	r = .803 p = .009	r = -.826 p = .006	r = -.711 p = .032
Change in PACS (wk 5-wk 4)			r = .762 p = .017	r = .726 p = .027
Change in AASE conf. (wk 5 – wk 4)			r = -.611 p = .081	r = -.494 p = .177

3.3.4 Recruitment, Pre-screening, and Informed Consent

Participants will be recruited from the community using advertisements in local media and flyers, a methodology we have used successfully in many prior studies. Recruitment will use a combination of the following methods: 1) Advertising in local print media; 2) Advertising in electronic media such as Craigslist; 3) Flyers placed in community locations where this is permitted. Those who are interested will be pre-screened using an IRB-approved pre-screening form including basic demographic data and questions related to inclusion and exclusion criteria. Those who pass pre-screening will be scheduled for a screening visit. Informed consent will be conducted by a trained investigator who is knowledgeable about the effects of hallucinogens as well as all aspects of the protocol. Details of the informed consent process are described in Section 4.

3.3.5 Randomization and Blinding

Randomization will be performed as close as possible to the first drug administration session (on the morning of the session if possible) in order to restrict the intent-to-treat sample to patients who actually receive study medication. The randomization will be stratified by site and will consist of balanced varied size blocks within strata to ensure relative equality of assignment across treatment groups. The block sizes will not be revealed to participating investigators and will be randomly selected from a small number of different block sizes to help reduce the likelihood of an investigator predicting the next treatment assignment. The study statistician will generate the randomization scheme for each site, and the study pharmacist will assign treatment sequentially according the sequence for each site.

3.3.6 Study Participants

Participants will be recruited from the community through advertising and referral, according to recruitment plans to be developed locally at each site.

3.3.6.1 Inclusion Criteria

Participants will be:

1) Males and females age 25-65 with SCID (DSM-IV) diagnosis of alcohol dependence who
2) Want to stop or decrease their drinking
3) Are not participating in any formal treatment for alcohol dependence (12-step meetings are not considered treatment)
4) Are able to provide voluntary informed consent
5) Have at least 4 heavy drinking days in the past 30 days
6) If female of childbearing potential, are willing to use approved form of contraception# from screening until after the psilocybin administration sessions
7) Have a family member or friend who can pick them up and stay with them overnight after the psilocybin administration sessions
8) Are able to provide adequate locator information.

3.3.6.2 Exclusion Criteria

Participants will be excluded if they have:

1) Medical conditions that would preclude safe participation in the trial (e.g., seizure disorder, significantly impaired liver function*, coronary artery disease, history of arrhythmia, heart failure, uncontrolled hypertension (above 165/95 mmHg at screening), history of cerebrovascular accident, severe asthma^, hyperthyroidism, narrow-angle glaucoma, stenosing peptic ulcer, pyloroduodenal obstruction, symptomatic prostatic hypertrophy, or bladder-neck obstruction)
2) Exclusionary psychiatric conditions (schizophrenia, schizoaffective disorder, bipolar disorder, current major depressive episode, current post-traumatic stress disorder, current suicidality or history of medically serious suicide attempt)
3) Cognitive impairment (Folstein Mini Mental State Exam [111] score < 26)
4) A family history of schizophrenia or schizoaffective disorder (first or second degree relatives), or bipolar disorder type 1 (first degree relatives)
5) History of hallucinogen use disorder, any use in the past 1 year, or >25 lifetime uses;
6) Cocaine, psychostimulant, opioid, or cannabis dependence (past 12 months)
7) Current non-medical use of cocaine, psychostimulants, or opioids (past 30 days)
8) Significant alcohol withdrawal (CIWA-Ar score greater than 7. Patients presenting at screening in withdrawal may be referred for detoxification and reassessed within 30 days)
9) Serious ECG abnormalities (e.g., evidence of ischemia, myocardial infarction, QTc prolongation (QTc > 0.45 seconds for men, QTc > 0.47 seconds for women))
10) Serious abnormalities of complete blood count or chemistries
11) Active legal problems with the potential to result in incarceration
12) Pregnancy or lactation
13) Need to take medication with significant potential to interact with study medications (e.g., antidepressants, antipsychotics, psychostimulants, treatments for addictions, other dopaminergic or serotonergic agents, lithium, anticonvulsants).
14) Allergy or hypersensitivity to psilocybin or diphenhydramine.
15) High risk of adverse emotional or behavioral reaction based on investigator's clinical evaluation (e.g., evidence of serious personality disorder, antisocial behavior, serious current stressors, lack of meaningful social support).

Participants with elevated blood pressure on screening (i.e., reading above 140/90 mmHg but no higher than 165/95 mmHg) who otherwise qualify for the study may be provisionally included and referred to their primary care provider for management of hypertension. For all participants, vital signs will be measured at all assessment visits, and may be monitored at therapy visits at the discretion of the study physician.

Participants will receive study medication only if blood pressure is less than or equal to 140 systolic, 90 diastolic at safety screening on the day of the drug administration sessions. Participants with a blood pressure reading greater than 165/95 at screening will be excluded from the study.

Participants with a BMI exceeding 35 at screening who otherwise qualify for the study will be administered a dose of study medication that corresponds to the body weight that would yield a BMI of 35 for their height.

[#]Acceptable methods of contraception include oral contraceptives, barrier with spermicide, IUD, levonorgestrel implant (Norplant), medroxyprogesterone acetate (Depo provera), surgical sterilization, contraceptive patch, vaginal contraceptive ring, and complete abstinence (not having sex).

*Significantly impaired liver function is defined as 1) Alanine aminotransferase (ALT) and/or aspartate aminotransferase (AST) > 5 × upper limit of normal (ULN); 2) ALT or AST > 3 × ULN with concomitant total bilirubin > 2.0 × ULN; or 3) ALT or AST ≥ 3 × ULN with the appearance of fatigue, nausea, vomiting, right upper quadrant pain or tenderness, fever, rash, and/or eosinophilia.

^Severe asthma is defined as asthma diagnosis along with any of the following: 1) an active or chronic cough, 2) current respiratory infection 3) current difficulty breathing 4) asthma-related emergency room visit, hospitalization, or oral, intravenous, or intramuscular steroidal treatment within the past 6 months. Participants with asthma diagnosis that are included in the study and are currently using an inhaler will be asked to bring their inhaler to the medication sessions.

3.3.7 Sites

Up to 4 sites will participate in the trial. Sites will be selected on the basis of experience with administration of classic hallucinogens, other clinical trial experience, available staff, available facilities, regulatory approvals, and institutional support.

3.3.8 Study Treatments

3.3.8.1 <u>Pharmacologic Interventions</u>

3.3.8.1.1 Psilocybin

Psilocybin has some advantages over other classic hallucinogens. Recent human studies with psilocybin confirm the relatively benign safety profile [40, 41, 97, 112]. Its duration of action (approximately 6 hours) is intermediate between that of LSD or mescaline and that of DMT. This duration makes it practical to administer during a session lasting no more than 8 hours, but, unlike DMT, allows ample time for the experience to be integrated into normal consciousness. Unlike DMT, it can be administered orally.

Psilocybin will be obtained from a laboratory capable of producing psilocybin meeting CMC requirements for use under IND 113080. Study drug will only be used in patients following acceptable testing results after shipment to the site. Psilocybin will be stored according to state and federal regulatory requirements for handling of a schedule I drug. The appropriate schedule I research licenses will be obtained at each institution.

3.3.8.1.2 Diphenhydramine

It is difficult to create a suitable blind when the active treatment elicits a powerful subjective effect. The purpose of a psychoactive control medication is to create uncertainty as to which medication was administered, and to minimize disappointment due to not receiving active medication. Niacin has been used in some studies because it causes flushing but is essentially a placebo [106, 113]. Griffiths and colleagues have used methylphenidate as a psychoactive control [97].

We propose to use diphenhydramine in the dose range of 50-100 mg as the control condition. In this dose range, diphenhydramine has noticeable subjective effects [114], and could possibly be mistaken for psilocybin by psychedelic-naive participants. Diphenhydramine also has a high index of safety and very low abuse potential [115-118]. There is no evidence that it is effective in the treatment of alcohol dependence.

3.3.8.1.3 Dosing and Administration of Study Medications

Setting. The psilocybin sessions will take place in outpatient facilities that are appropriate for hallucinogen administration sessions. To the extent possible, the room used for the session will be specially prepared for the sessions to provide a warm and home-like rather than a stark clinical quality because of the large influence that setting can have on the subjective effects of hallucinogens. Each site will have detailed SOPs in place for managing medical or psychiatric emergencies should any occur.

Safety Screening on the Day of Drug Administration Sessions. Participants will be required to attempt to remain abstinent during the week preceding the drug administration sessions. On the morning of the psilocybin session, participants must have a negative breathalyzer reading, drug screen must be negative for all non-prescribed tested drugs of abuse except for marijuana, CIWA–Ar score must be less than 8, and vital signs must be within normal limits (afebrile, blood pressure less than or equal to 140 systolic, 90 diastolic, resting pulse less than 100). Female participants of childbearing potential must also have a negative urine pregnancy test.

Dosing of Study Medications. Individualized doses of psilocybin will be prepared by the site pharmacist using an analytical balance. Medication will be placed in an opaque gelatin capsule prior to administration, with the balance of the capsule volume filled with microcrystalline cellulose.

Although the optimal dose of psilocybin in the treatment of alcohol dependence is unknown, several arguments suggest that strong effects are likely to be predictive of therapeutic benefit. The controlled trials of LSD for alcohol dependence used relatively high doses of LSD (median dose 500 mcg [1]) which typically produce very strong effects. The “peak-psychedelic” experience was widely believed to be the active ingredient in LSD therapy for alcoholism. In published work with normal volunteers Griffiths et al. reported that that highest dose used (30 mg/70 kg) produced the strongest effects and the highest frequency of “complete mystical experience” based on their scores on the Pahnke-Richards Mystical Experience Questionnaire [97, 110]. The degree of mystical experience predicted spiritual significance and personal meaningfulness at 14 mo [119]. Participants who had a “complete mystical experience” had significant persistent (14 mo.) increase in the personality dimension of “openness” [120]. The 0.43 mg/kg dose was generally well-tolerated, although fear, anxiety, and dysphoria were common side effects, and 6/36 participants undergoing a single session at this dose reported mild, transient paranoia or ideas of reference at some point during the session [97]. None of the volunteers reported persisting adverse effects or rated the session as having decreased their well-being or satisfaction with life.

There are reasons to believe that higher doses of psilocybin may be needed to achieve strong effects in some alcoholics than the doses that reliably produce such effects in normal volunteers. In our pilot work doses of psilocybin 0.3 mg/kg and 0.4 mg/kg have been well-tolerated in alcohol dependent participants, but responses demonstrated that some alcohol dependent subjects have less pronounced response to psilocybin than those reported by normal volunteers, although others have strong reactions typical of non-alcoholic individuals. This is consistent with observations beginning in the 1950s that alcoholics tended to show greater tolerance to the effects of LSD [107].

To maximize the probability that participants treated with psilocybin will have strong and meaningful experiences while minimizing the chance of significant psychological distress or other adverse events, we propose to titrate the dose of study medications in the second session based on the response in the first session. In the first session, participants in the psilocybin group will receive a moderately high dose of 25 mg/70 kg. For the second session, the dose will be titrated within a range of 25-40 mg/70 kg, depending on the participant’s response in the prior session. The dose will be increased if the participant tolerated the 25 mg/70kg dose well (no clinically significant adverse psychiatric or medical effects) and is willing to increase the dose. The dose will be increased to 30 mg/70kg if the participant had reported strong subjective response to the first session, as evidenced by a total score on the MEQ ≥ 0.6. The dose will be increased to 40 mg/70 kg only if the participant a) had a relatively mild response to 25 mg/70 kg (MEQ total score < 0.6) and b) the therapists and participant agree that increasing the dose to 40mg/70kg will be safe and will maximize likelihood of clinical benefit. The dose may be held at 25 mg/70 kg if the participant is not willing to increase the dose. The second session will be canceled if the participant is not willing to undergo a second session, or if he or she experienced clinically significant adverse psychiatric or medical adverse

events related to the first session. Figure 4 illustrates the dosing algorithm. The first and second decision points (whether to conduct the second session and whether to increase the dose) will be determined by the study clinicians in collaboration with the participant. The third decision point (how much to increase the dose will be determined by the total score of the Mystical Experience Questionnaire (MEQ), scored by a trained research staff member not participating in the study treatment.

In the control condition, the dose of diphenhydramine will be 50 mg in the first session. In the second session the dose will be increased to 100 mg if the participant tolerated the 50 mg dose well (no clinically significant adverse psychiatric or medical effects) and is willing to increase the dose. If the participant has no clinically significant adverse psychiatric or medical effects but is unwilling to increase the dose, the dose will be held at 50 mg. If the participant is not willing to undergo a second session or experienced clinically significant adverse psychiatric or medical effects related to the first session, the second session will be canceled. Participants who decline to participate in the second medication session of the double-blind phase, but otherwise complete the double-blind phase of the trial, and did not experience clinically significant adverse psychiatric or medical effects related to the first medication session, may participate in the third, open-label medication session.

Figure 4: Dose Determination for Second Drug Adminstration Session

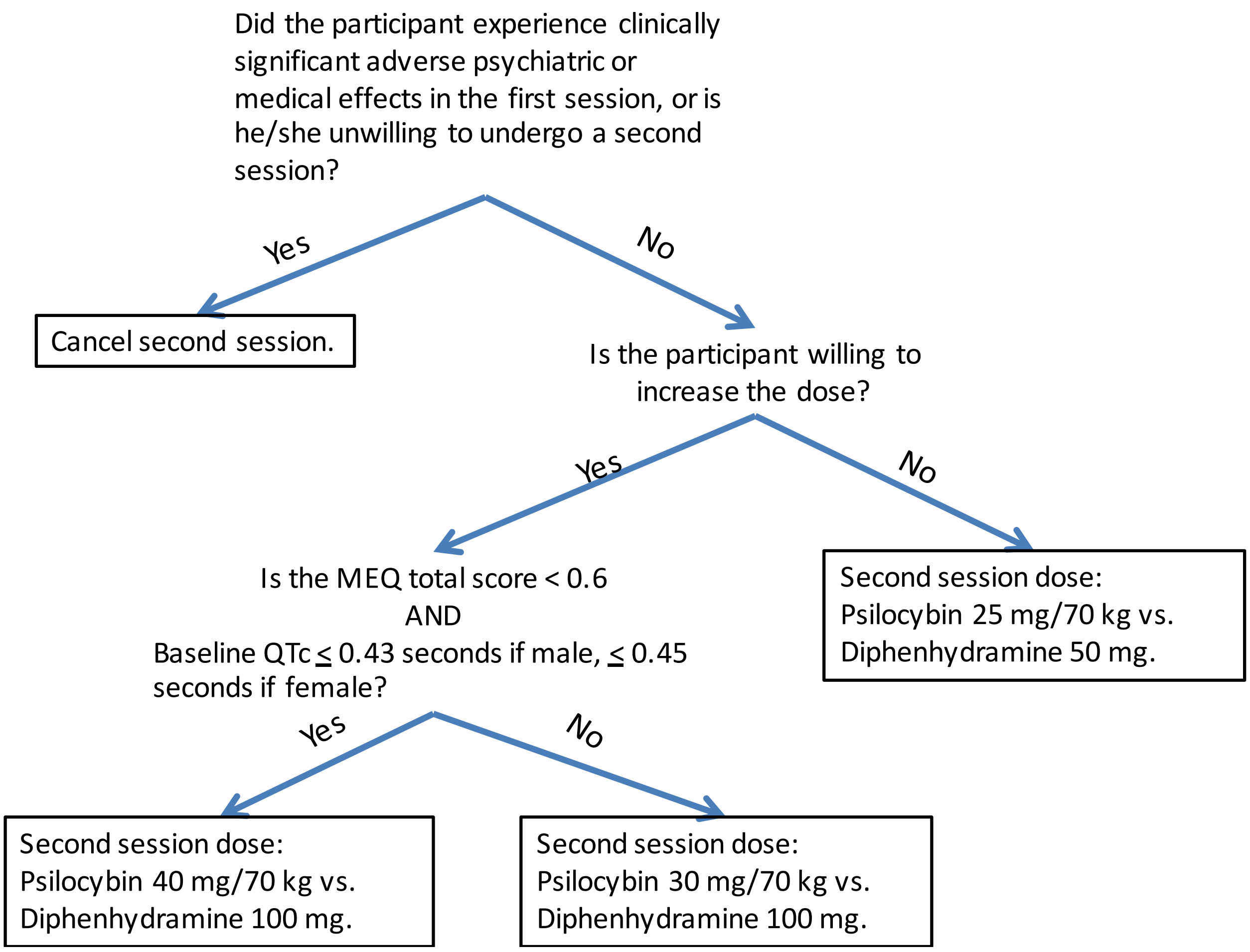

Prior to the third session, ECG will be repeated. The dose will again be capped at 30 mg/70 kg if QTc >0.43 seconds or >0.45 seconds for men and women, respectively. The session will be canceled if QTc >0.45 seconds or 0.47 seconds for men and women respectively. Otherwise, in the third session, participants in the diphenhydramine group will receive psilocybin 25 mg/70 kg, and participants in the psilocybin group will receive the same dose they received in the second session, unless they request a lower dose, in which case they will receive a dose of 25 mg/70kg.

The pharmacologic treatment will be terminated after the first or second session if adverse events during or after the session suggest that another session would pose significant risk, if there are any significant persisting negative effects from the first session, or if the participant does not wish to complete an additional session. If pharmacologic treatment is terminated, the participant still will complete the psychosocial treatment.

Monitoring and Safety Procedures. As psilocybin use is associated with a small risk of emesis, participants will be instructed to have only a light breakfast on the morning of the psilocybin administration sessions, at least an hour before the beginning of the session. Participants will complete assessments including confirmation of medical stability and drug- and alcohol-free status. (If the participant has CIWA-Ar score of 7 or greater, need for detoxification will be assessed and the patient will be referred for treatment if indicated). They will then ingest the capsule containing the study medication, followed by approximately 4 ounces of water. Participants will remain under observation for at least 8 hours following psilocybin administration (maximal effects are expected to occur at approximately 2-3 hours). Two therapists will be present throughout all psilocybin administration sessions (except for breaks, which will be taken one at a time). Interaction with the participants will be supportive and non-directive. Vital signs will be monitored by study staff at 30 minute intervals for three hours, and then hourly. At the time of vital sign measurement, the therapists will also check in with briefly with participants and complete a brief Monitor Rating Questionnaire. Management of hypertension will be as follows. If BP is greater than or equal to 180 systolic or 100 diastolic, subjects will be assessed for discomfort that may be causing the BP elevation, e.g. anxiety, headache/pain, or alcohol withdrawal. These symptoms should be addressed/medicated, and BP will be reassessed at 15 minute intervals until below 180/100. If patient has >2 BP readings of 200/110 or higher, then the BP will be treated directly. At this time, the patient should specifically be assessed for new chest pain, shortness of breath, or focal weakness, in which case the subject will need more urgent medical attention. The following medications will be available for administration by the study physician if needed to treat hypertension (captopril 25 mg orally/sublingually, clonidine 0.1 mg orally, or hydralazine 10 mg orally). In the event that a subject experiences distressing anxious or psychotic symptoms during the experimental or control session, the clinical personnel in attendance will initiate verbal communication designed to reorient and reassure the subject. If that does not effectively alleviate the signs of an anxious or psychotic reaction, diazepam 5-10 mg, orally, or lorazapam 1 mg, intramuscularly, will be administered to lower anxiety. Only if that is ineffective or if the participant presents a danger to self or others will an antipsychotic be administered, olanzapine 5-10 mg, given either orally or intramuscularly. For headache or pain, Tylenol 500-1000 mg and/or Ibuprofen 600 mg-800 mg may be administered once orally.

Beginning 7 hours following drug administration, participants will complete questionnaires and assessments, and a study therapist will complete a brief clinical assessment, including mental status exam, recorded in the progress note. The mental status exam includes the following:

- Appearance
- Behavior
- Speech
- Thought process (ask specifically about persisting hallucinations)
- Thought content (ask specifically about delusional material, suicidality, homicidality)
- Affect and mood
- Cognitive function
- Judgment and insight

If there are no residual effects posing significant risk, the participant will be escorted home at the end of the session by a family member or friend, who will stay with the participant overnight. A study clinician will be available to participants by pager or phone at all times during study participation. If the clinical assessment reveals evidence of any residual effects that could pose a risk to the patient or others (e.g., suicidal thoughts, psychotic symptoms), the therapists will stay with the participant until the symptoms resolve or it is determined that further treatment is required. In the latter case, the participant may be taken to the psychiatric emergency service for further assessment, observation, or even psychiatric admission if deemed clinically necessary.

3.3.8.2 Behavioral interventions

For the current randomized trial we propose to keep the psychosocial treatment procedures very similar to those used in the pilot and described in the therapy manual. Figure 2 illustrates how the behavioral interventions interface each other and with the three drug administration sessions. For simplicity, the behavioral interventions that have to do with the drug administration sessions will be referred to as Preparation, Support, and Integration (PSI). The alcohol therapy component consists of Motivational Enhancement Therapy (MET) followed by cognitive behavioral therapy sessions including implementation of specific strategies for change during the later sessions. To distinguish it from more typical MET approaches, this therapy will be called Motivational Enhancement and Taking Action (META).

As in the pilot study, the therapy will be conducted by a team of two therapists, one responsible for the alcohol-specific treatment (META), the other responsible for the hallucinogen-specific treatment (PSI). The two therapists forming a dyad should be of opposite genders if possible, and should never be both of the gender opposite that of the patient. The META therapist may conduct the META sessions alone, and will also attend the PSI sessions facilitated primarily by the PSI therapist. The PSI therapist may conduct preparation and debriefing sessions alone if necessary, but both therapists must attend the drug administration sessions. In the event that in person visits are not possible, therapy visits may be held via phone or WebEx. Substitutes for drug administration sessions may be used only if they have had at least two meetings with the participant prior to the session. The separation of roles is intended to maintain clarity about the purpose of the sessions, while the attendance of the META therapist at PSI sessions allows the two components to function as an integrated whole. A practical advantage to the separation of roles is that each therapist only needs to have a high degree of expertise in his or her respective area.

3.3.8.2.1 Motivational Enhancement and Taking Action (META)

The META content will be based on procedures used in the UNM pilot study, which are largely based on materials used previously in multisite trials [103, 104, 121]. Because the number of sessions indicated is greater than the 3-4 sessions typically used in MET, and because of the relatively high motivation for change seen in many of the pilot study participants, the META manual developed for this protocol will include greater emphasis on exploration of a patient's goals for change and the development and implementation of specific strategies to meet those change goals.

The goal of the first session is for the therapist to use open motivational interviewing to elicit and clarify the patient's intrinsic motivation for change. During this session, therapists will also offer feedback from the baseline assessment, specifically focusing on drinking percentile relative to population norms, consequences of drinking, and motivation for change. The focus of the second session will be on exploring the patient's values (including spiritual values) and the discrepancy between values and behavior as motivation for change.

The two subsequent META sessions (sessions 3 and 4) will follow up on the patient's goals for change and experiences during the first medication session in relation to the key values identified during META session two. The focus of these sessions is also for the therapist and patient to negotiate a specific treatment plan for the remaining sessions. Using the structure of the behavioral STORC model (situation, thoughts, organic patterns, response, and consequences), therapists will work with patients to identify specific components of STORC sequences in which the patient seems to be encountering difficulties. At each step in the STORC cycle there are usually a number of things that can be done to promote change. For each change goal a patient identifies, therapists will offer a menu of options for strategies potentially useful in facilitating change (e.g., the Situational Factors Menu will include modules on how to identify problem situations, monitoring urges, ways to change your environment, how to ask others for help, and how to surround yourself with support; the Organic Patterns Menu will include modules on exercise, mindfulness practice, sleep hygiene, and nutrition).

In contrast to the first four sessions which are highly structured in that all patients receive similar content on feedback, values exercise, and structuring a treatment plan, the remaining sessions will be individualized to the unique needs of each patient. Specific pull-out teaching modules with worksheets--to be

completed during and between sessions--will offer structure for therapists in the delivery of this portion of the intervention. This will allow therapists to use flexible discretion as to the content of these sessions as the needs of the patient evolve. Activities that may be used during these sessions will include 12-step or other self-help involvement, mindfulness practice, exercise, changes in social network, cognitive behavioral self-help, alternative sources of positive reinforcement that do not involve substance use, or further formal alcohol treatment. The plan for change will be revisited and revised as needed during each session, and therapists will reinforce progress and revise the plan as needed in collaboration with the patient. Consistent with the MI style, these remaining sessions are also intended to be a time to re-engage the patient, continue discussions on experiences and feelings resulting from the medication sessions, support continuing efforts, and address any barriers to goal achievement.

3.3.8.2.2 Preparation, Support, and Integration (PSI)

These procedures will be very similar to those defined in the manual used in the UNM pilot study.

Preparation sessions. There are two preparation sessions before the first drug administration session, and one before the second drug administration session. The primary goals for the first preparation session are to provide an overview of the process and rationale of the study intervention; to allow the clinicians to gather information about the participant's history, current situation, personality, and values; and to facilitate the development of rapport between the participant and the clinicians. The second session includes a review of motivation and expectations for the study; detailed information about the possible physiological and psychological effects of study medication; advice as to how to deal with dysphoric reactions to study medication, should they occur; identification of any personally meaningful items that the participant will bring to the session (e.g., images, family photographs, objects of personal or religious significance); discussion of ground rules for the session; and addressing questions, concerns, hopes, and fears related to the medication-assisted treatment. In the third preparation session, each topic is revisited, plans are revised based on the experience in the prior drug administration sessions, and the therapists and participant decide on the dose of medication to be used in the second drug administration session.

Drug Administration Sessions. Beyond the monitoring procedures described above, the interventions employed during the drug administration sessions are intended to help the participant use the session as productively as possible, rather than to provide directive therapy. Participants wear eyeshades and listen to a standardized program of music through headphones during most of the session. Brief check-ins are used to assess the participant's mental state. Therapists may provide reassurance, support, grounding, and redirection as needed. Participants are encouraged to focus on their internal experience as much as possible, and to "trust, let go, and be open" to the experience rather than try to direct or control it. Once the drug effects have largely subsided (after 5-6 hours) participants may spend increasing amounts of time interacting with the therapists and discussing the content and meaning of the experience. Participants are asked to write down an account of the experience during the evening after the experience, for discussion at subsequent debriefing sessions.

Debriefing Sessions. A debriefing session is scheduled the day after each drug administration session. The basic content of these sessions includes open-ended inquiry concerning the drug administration session and invitation to reflect on the experience. Participants are invited to consider the meaning and implications of the experience, including any changes in views of self, relationships, values, and spirituality. Using the motivational interviewing style, therapists will elicit discussion of how the session has affected the participant's relationship to alcohol and desire to change drinking behavior. Safety assessment will also be completed (mental status exam and follow-up on any adverse events).

3.3.8.2.3 Therapist Selection, Training, Supervision, and Fidelity Monitoring

It is important to specify the content of behavioral treatments in pharmacologic trials, and to ensure fidelity to the specified content. This is particularly important in studies of classic hallucinogens, since it is expected that the therapeutic benefits of the medication could be moderated by non-pharmacologic components of the treatment. We have adapted the procedures used in the COMBINE trial, streamlining them to some extent because the behavioral treatments are not being manipulated experimentally in the

current study, serving rather as the platform for the pharmacologic interventions. All sessions will be audio-recorded if possible. Checklists and rating forms for all components of the therapy will be developed based on those used in COMBINE. These will serve three purposes: to provide guidance to therapists in conducting sessions and allow them to document their self-assessed adherence; to provide material for supervision at the local level; and to provide material for formal fidelity monitoring, which will be done centrally at UNM.

Therapist Qualifications. Both the META and PSI therapists must have at least a master's degree in a related field or be pursuing a master's degree. At least of one or both of the META and PSI therapists must be a licensed physician (MD) for all sessions.

Training and Certification. Overlapping training requirements and procedures are established for the PSI therapists and the META therapists. Therapists in both roles will be required to read the entire therapy manual (covering both PSI and META procedures). Both will be required to attend a 2-day basic training in motivational interviewing and a 1-day basic training on hallucinogen-assisted therapy (unless they have already attended such trainings). In addition, the PSI therapists will attend an additional 1-day training covering the specific manualized approach used in this trial. Similarly the META therapists will receive an additional 2 days of training in the META intervention used in the current trial. In order to receive provisional certification for the type of treatment they will be conducting (META or PSI), therapists will be required to complete one case with a simulated patient (staff member or actor), and receive satisfactory ratings from the Fidelity Monitor for their type of treatment. Up to two additional training cases may be completed if the first one is not satisfactory. Following provisional certification, therapists may begin seeing patients in the training/pilot phase (see Section 3.3.8.2.4 below) or main trial participants. The first case will be rated in its entirety by the Fidelity Monitor, and therapist will receive full certification following satisfactory completion of the first case.

Supervision. Supervision will be conducted locally, and will be the responsibility of a designated lead PSI therapist and lead META therapist at each site. Study clinicians will use session checklists to document adherence to the model. Cases will be discussed in weekly group supervision including all active therapists at the site. The lead therapists will review one META and one PSI session per case for each additional therapist at the site, score them using session checklists, and provide feedback to the therapists. The Fidelity Monitor will have monthly conference calls with the lead therapists, and will periodically review session tapes (including those of the lead therapists) as needed. Patterns of unsatisfactory performance will result in a warning from the Fidelity Monitor, and the Supervisor will implement a performance improvement plan. Persistent or serious deviation from the prescribed treatment model may result in removal of the therapist from the trial until the therapist completes a remedial training plan and demonstrates competence with simulated patients. All session recordings will remain available for further coding and/or qualitative analysis.

3.3.8.2.4 Training/Pilot Phase

At each site, at least 4 participants will be treated in a training/pilot phase prior to recruitment of participants in the main trial. These cases will be randomly assigned to one of the two treatments in blocks of 4, independent of the main trial. Therapists and participants will be blinded to treatment assignment. Pilot participants will be included in the main trial sample if 1: Therapy fidelity ratings are adequate, and 2: No protocol modifications are made that would invalidate their inclusion.

3.3.9 Concomitant Therapy

Participants should not be actively engaged in other forms of addiction treatment during the treatment phase of the study. Ongoing counseling or therapy for conditions other than substance use disorders is permitted. Most psychotropic medications are excluded until after the double-blind drug administration sessions. Excluded medications include antidepressants, antipsychotics, psychostimulants, pharmacologic treatments for addictions, other dopaminergic or serotonergic agents, lithium, and anticonvulsants. Other concomitant medications will be reviewed during the screening medical assessment, and a study physician-investigator will determine whether the participant should continue the medication, temporarily discontinue it, or be excluded from the study. If excluded medication is started or resumed during the double-blind follow-up period (i.e,

during weeks 9-38) and the participant is interested in completing in the open-label session, the study physician will consider whether it is clinically appropriate to taper the medication in preparation for the open-label session, consulting with the participant's prescriber as needed. Examples of possibly appropriate situations include psychostimulant treatment of ADHD or antidepressants for mild depression or anxiety disorder. If the risks of a taper are deemed acceptable and the participant agrees, the medications may be tapered by the participant's prescriber as clinically appropriate. The excluded medications must be completely discontinued for 5 half-lives or one week, whichever is greater, prior to the open-label medication session.

3.3.10 Measures

Measures are listed in Table 1 below, organized by domain.

**Table 1. Assessment Schedule**														
**Assessments by Visit**		**Scr.**	**BL**	**W04**	**W05**	**W08**	**W09**	**W12**	**W24**	**W36**	**W38**	**W39**	**W42**	**W54**
**Medical evaluation**	History and physical, liver function tests, complete blood count, Chem 7, U/A, serum pregnancy test, height and weight, medical history intake form	550'												
	ECG	5'								5'				
	Concomitant meds, menstrual calendar	5'	5'	5'	5'	5'	5'	5'	5'	5'	5'	5'	5'	5'
	Urine pregnancy test			5'		5'					5'			
**Psychiatric and SUD assessment**	Structured Clinical Interview for DSM-IV (SCID), Family History Questionnaire, Folstein MMSE	75'												
**Other baseline characteristics**	Attentional Resource Allocation Scale, Treatment Goal Form, Demographics Form, Locator Form		15'											
**Withdrawal**	CIWA-Ar	5'		5'		5'					5'			
**Substance use and consequences**	Time-line Follow-back, BAC	30'	10'	10'		10'		10'	10'	10'	10'		10'	10'
	Hair/ Nail sample for ethyl glucuronide								5'					
	Urine Drug Screen	5'		5'		5'		5'	5'	5'	5'		5'	5'
	Short Inventory of Problems (past 3 months)		5'					5'	5'	5'				5'
**Acute Hallucinogen Effects**	5D-ASC, HRS, SCQ, Monitor Rating Scale, Mysticism scale, guesses of medication received, MUSE questionnaire			30'		30'					30'			
**Persisting Effects**	Mysticism Scale (lifetime), Spiritual Transcendence Scale, Persisting Effects Questionnaire (follow-up		90'							90'				90'

	only), BMMRS, NEO-PI-3, Schwartz Value Survey													
**Motivation**	Readiness rulers		5’	5’	5’	5’	5’	5’	5’	5’	5’	5’	5’	5’
**Self-efficacy**	Alcohol Abstinence Self-efficacy Scale		10’	10’	10’	10’	10’	10’	10’	10’	10’	10’	10’	10’
**Craving**	Penn Alcohol Craving Scale		5’	5’	5’	5’	5’	5’	5’	5’	5’	5’	5’	5’
**Self-Compassion**	Self-Compassion Scale (Short Form)		5	5	5	5	5	5	5	5	5	5	5	5
**Mood/ Anxiety**	Ham-D, Ham-A		20’	20’	20’	20’	20’	20’	20’	20’	20’	20’	20’	20’
**Safety**	Adverse Events Case Report Form			5’	5’	5’	5’	5’	5’	5’	5’	5’	5’	5’
	Vital Signs and weight	5’	5’	5’	5’	5’	5’	5”	5’	5’	5’	5’	5’	5’
	Visual Analog Scales for abuse potential							5’	5’	5’			5’	5’
**Tx satisfact.**	Visual Analog Scales							5’					5’	
	**Assessment Length**	**185’**	**175’**	**110’**	**55’**	**110’**	**55’**	**85’**	**85’**	**170’**	**110’**	**55’**	**75’**	**170’**

3.3.10.1 Medical Evaluation

Medical screening will consist of medical history and physical, ECG, liver function tests, complete blood count, Chem 7, U/A, serum pregnancy test, height, and weight. Labs may be repeated if needed to ensure safety. Concomitant medications will be recorded at screening and updated at each visit. Women of childbearing potential will complete a menstrual calendar at each assessment visit, and urine pregnancy tests will be completed prior to each drug administration session. Vital signs will be measured at each assessment visit, and during the psilocybin sessions (q 30 minutes for 2 hours, then hourly to 6 hours post administration).

3.3.10.2 Psychiatric and Substance Use Disorder Assessment

For Axis I diagnoses we will use the Structured Clinical Interview for DSM-IV (SCID), a structured interview which yields DSM-IV diagnoses for Axis I disorders including substance abuse and dependence diagnoses [122]. To allow DSM-5 substance use disorder diagnoses to be made as well, an item will be added for the DSM-5 craving criterion. Excellent inter-rater reliability of the DSM-III-R version of the SCID has been reported for schizophrenia, major depression, and substance use disorders (kappas ranging from 0.85 to 0.96), with adequate to excellent reliability for other mood and anxiety disorders, with the exception of obsessive-compulsive disorder [122]. . Family history of addiction and exclusionary psychiatric disorders will be recorded using a Family History Interview. The Folstein Mini–Mental State Examination [111] will be used to screen for cognitive impairment. A score below 26 will exclude subjects from the study.

3.3.10.3 Alcohol Withdrawal

The Clinical Institute Withdrawal Scale—Alcohol, revised (CIWA-Ar) [123] will be used to assess alcohol withdrawal at screening and before the psilocybin sessions.

3.3.10.4 Substance Use

3.3.10.4.1 Self-report

The Time-line Follow-back (TLFB) [124, 125] procedure will be used to assess drug use behavior at baseline and follow-up visits. The TLFB is a semi-structured interview that provides estimates of the daily quantity, frequency, and pattern of drug use during a specified time period. It uses a calendar prompt and number of other memory aids (e.g., holidays, payday, and other personally relevant dates) to facilitate accurate recall of drug use during the target period. The TLFB has shown adequate to excellent reliability and validity over a wide range of research and clinical contexts [100, 126, 127]. It is estimated that the

TLFB assessment will take 10-30 minutes to complete, depending on the length of the assessment period and the patterns of alcohol and drug use. Non-study treatment and 12-step attendance will also be recorded on the TLFB form.

3.3.10.4.2 Objective Measures of Alcohol and Drug Use

Hair/ nail analysis for Ethyl glucuronide (HEtG) Hair/nail samples will be obtained at 24 weeks (20 weeks after the first drug administration session). HEtG is a sensitive and specific measure of recent alcohol consumption, with a cut-off of 27 pg/ng detecting heavy drinking (≥ 60g alcohol/day) at sensitivity of .92 and specificity of .96 [128, 129]. A 3 cm proximal sample of hair (scalp hair if possible) representing a 3-month history of alcohol use will be obtained at week 24 (20 weeks after the first drug administration session). If participants do not have sufficient hair, then fingernails (2 mm from each of 10 fingernails) may be used as an alternative. Samples will be analyzed quantitatively using a liquid chromatography–tandem mass spectrometry (LC-MS/MS) method. Results may be used categorically.

Breath Alcohol Concentration (BAC) will be measured using the Alcosensor III ® or a similar device, which will be calibrated monthly.

Urine Drug Screen (UDS) We will use the Alere™ iCup ® or similar chromatographic immunoassay to test for use of marijuana, cocaine, opiates, and methamphetamine.

3.3.10.5 Substance Use Consequences

The Short Inventory of Problems (SIP-2R) [101], past 3 month version, will be used to measure consequences of alcohol use. The SIP-2R is a 15-item measure that assesses five domains of alcohol-related consequences: (1) social, (2) intrapersonal, (3) interpersonal, (4) impulse control, and (5) physical. Derived from the 50-item Drinkers Inventory of Consequences (DrInC), the SIP-2R has excellent reliability and validity.

3.3.10.6 Acute and Persisting Hallucinogen Effects

3.3.10.6.1 Acute Hallucinogen Effects

5-Dimensional Altered States of Consciousness Scale (5D-ASC) [130] will be used to quantify acute hallucinogen effects. This scale has 94 items using the visual analog scale format, yielding 5 primary dimensions. The primary dimensions are “oceanic boundlessness,” “anxious ego-disintegration,” “visionary restructuralization,” “acoustic alterations,” and “altered vigilance.”

The Hallucinogen Rating Scale (HRS), developed and validated at the University of New Mexico by Rick Strassman et al. (1994) in studies of intravenous DMT and validated in ayahuasca users [131], will also be administered following each drug administration session. It is being used by the Hopkins and Tucson groups in their psilocybin research, and has been used to assess effects of a wide variety of psychoactive drugs including MDE, methamphetamine, psilocybin, ibogaine, MDMA, ayahuasca, methylphenidate, d-amphetamine, ketamine and mCPP. This 99-item scale has 6 subscales: intensity, somaesthesia, affect, perception, cognition, and volition.

The Mysticism Scale will be used to assess “mystical” dimensions of the hallucinogen experience. This 32-item, 3-factor questionnaire has been used in studies of non-drug-induced mystical experiences [132], and has also been used in the psilocybin work of Griffiths et al. (2006).

The States of Consciousness Questionnaire (SCQ) will also be administered. This 100-item questionnaire has been used extensively to measure mystical-type states of consciousness in hallucinogen administration experiments [97, 113, 133-135]. This scale contains the 43 items of the Pahnke-Richards Mystical Experience Questionnaire (MEQ) [97].

A 20-item Monitor Rating Scale [97] will be completed by both therapists at intervals during the psilocybin sessions (every 30 minutes for the first 2 hours, then hourly) to provide objective ratings of participants' behavior and affect during the session.

A novel Music and Sound Experience (MUSE) questionnaire will be administered at the end of each medication session. The questionnaire contains 20 items that assess the impact the music and sounds played during the medication session had on the quality of the participant's experience.

3.3.10.6.2 Persisting Hallucinogen effects

The Persisting Effects Scale [97] is based in large part on items used by Pahnke (1963; 1969), and includes subscales related to positive and negative changes in attitudes, mood, social functioning, and behavior.

The Mysticism Scale (lifetime version) and the ASPIRES Spiritual Transcendence Scale will also be used as measures of persisting effects. The Spiritual Transcendence Scale is a scale addressing the individual's sense of connectedness and meaning [136]. The scale yields a total score and three empirically derived factor scores: prayer fulfillment, universality, and connectedness. We will use the 13-item short form.

The Brief Multidimensional Measure of Religiousness/Spirituality (BMMRS) [137] will be used as a broad measure of multiple domains of spirituality.

The NEO Personality Inventory 3 (NEO-PI-3) [138] will be used to assess personality at baseline, 12, and 36 weeks as a recently published study has shown that psilocybin administration can cause significant persisting increases in the personality dimension of "openness" [120]. The NEO-PI-3 is an updated version of the NEO-PI-R [139]. The NEO inventories measure the personality dimensions of the 5-factor model: neuroticism, extraversion, openness, agreeableness, and conscientiousness, with 6 "facets" within each factor.

The Schwartz Value Survey [140] will be used to assess participants' values and possible shifts in relation to treatment. This instrument includes 57 items organized into 10 cross-culturally valid dimensions of value: self-direction, stimulation, hedonism, achievement, power, security, conformity, tradition, benevolence, and universalism [141].

3.3.10.7 Potential Mediators of Therapeutic Effect

3.3.10.7.1 Motivation

Readiness Rulers will be used as to measure motivation in the course of the trial, [142]. This simple measure comprises Likert-scale ratings of the participant's perception of the importance of change in drinking, confidence of ability to change, and readiness for change. Commitment to the goal of abstinence will be added as a fourth item.

3.3.10.7.2 Self-efficacy

The Alcohol Abstinence Self-Efficacy Scale (AASE) [143] is a self-report questionnaire which has been used widely in the alcohol treatment research, both as a predictor of outcome and as a client-treatment matching variable [144, 145]. Item content of the AASE-C subscale asks the respondent to rate the confidence they have to avoid drinking in different situations. In time-ordered analyses, a relationship has been demonstrated between the AASE-C and AA behaviors and drinking, both at 6 months [146] and 3 years after completing outpatient treatment [147].

3.3.10.7.3 Alcohol craving

The Penn Alcohol Craving Scale [148] will be used to assess craving. This scale has 5 Likert-scaled items with excellent internal consistency and evidence of predictive, construct, and discriminant validity.

3.3.10.7.4 Self-compassion

Self-compassion is a construct, derived from Buddhist psychology, which denotes a kind and non-judgmental attitude toward oneself [149]. A reliable and valid 12-item form of the Self-Compassion Scale [150] will be used as measure of self-compassion.

3.3.10.7.5 Mood and Anxiety

The Hamilton Anxiety (Ham-A) [151] and Depression (Ham-D) (17-item) [152] scales will be used as measures of depression and anxiety at baseline and at each follow-up visit.

3.3.10.7.6 Treatment Satisfaction

Visual Analog Scales will be used to assess participants overall satisfaction with study treatment (separate scales for satisfaction and usefulness).

3.3.10.8 Potential Moderators of Acute and Persistent Effects of Psilocybin

In addition to the participant characteristics measured above, the following measures will be obtained at baseline.

3.3.10.8.1 Dissociation and Absorption

The Attentional Resource Allocation Scale (ARAS) [153] will be used as measure of dissociation and absorption. This questionnaire is derived from factor analyses of two large samples administered both the a composite of the Tellegen Absorption Scale (a 34-item measure of absorption) [154] and the Dissociative Experiences Scale (a 28-item self-report questionnaire) [155] (items of both scales were Likert-scaled in this study). Exploratory and confirmatory factor analyses yielded 15 items loading on three highly intercorrelated factors: imaginative involvement, dissociative amnesia, and attentional dissociation. The single factor model also produced acceptable (but statistically inferior) fit indices.

3.3.10.8.2 Thoughts About Abstinence

Using the form that was used in the COMBINE trial [156], participants will be asked to endorse one of seven treatment goals which can be categorized as: 1) total abstinence; 2) conditional abstinence; and 3) moderation.

3.3.10.8.3 Demographics form

A standard Demographics form will be used to capture information such as ethnicity, education, and employment.

3.3.10.9 Safety Assessment

Adverse events (AEs), when present, will be collected on an AE Case Report Form at the end of the psilocybin sessions and at all subsequent visits. The form will include an assessment of clinical significance and study relatedness. Serious Adverse Events (SAEs) will be documented on an additional SAE form. These CRFs will be based on those used in recent NIDA Clinical Trials Network trials. Vital signs will be obtained at each visit and measured frequently during psilocybin sessions: every half hour for the first 2 hours, then hourly for a total of 8 hours, with more frequent readings as needed (See Therapy Manual). To further assess abuse potential, visual analog scales will be used to assess abuse potential. Scales will include desire to use the study medication again, desire to use the study medication again for pleasurable intoxication ("to get high"), and craving for the study medication.

3.3.10.10 Therapist and Participant Guesses of Treatment Assignment

In order to assess the integrity of the blind, following each drug administration session participants and therapists will be asked to guess 1) what medication was administered (psilocybin or diphenhydramine), and 2) degree of confidence in this guess.

3.3.10.11 Locator form

A standard locator form will be used to collect information on contact who can help locate participants in case they change address and/or phone numbers. Ideally at least 3 independent contacts will be identified.

3.3.11 Data Management and Procedures

Raw data in case report forms (CRFs) will be reviewed for completeness and accuracy at each visit. CRFs and source documents will be stored in locked cabinets in an office which will be locked when the office is not in use. Identifying data (e.g., consent) will be stored in separate locked cabinets. Data will also be entered into "Research Electronic Data Capture" (REDCap™) hosted at each participating site. At the conclusion of this study de-identified data will be downloaded and the project will be deleted off of REDCap™.

Data will be kept for at least 5 years following completion of the study. No PHI data will be kept unless subjects sign the informed consent form. PHI data of individuals who do not sign an informed consent form will be destroyed. Visits may be conducted in-person or via phone and data will be entered either by direct electronic data capture (e.g., tablet computers) by a research assistant or by the participant, or by independent double entry into an encrypted REDCap™ database located on a server and accessible only to approved staff study through a password-protected computer. The study database will be set up not to accept out-of-range values.

3.3.12 Statistical analysis and power

A full description of the planned analyses for this trial can be found in the Statistical Analysis Plan (SAP)

3.3.12.1 Preliminary analyses

In addition to the assessment of the distributional properties of key measures at baseline and follow-up, we will evaluate the nature and pattern of missing data and assess treatment group equivalency at baseline on primary substance use measures and patient characteristics that are central to study aims.

Self-selection and Attrition. Baseline measures of substance use and other relevant characteristics will be used to compare eligible prospects who do and do not drop out prior to randomization and receiving study medication. Contingent on the scale of measurement of the comparison variable, unprotected t-tests and Chi Squares will be done to compare these groups. Two strategies will be used to examine the impact of post-randomization attrition on study internal validity, and both strategies will employ hierarchical linear modeling (HLM) with binary outcomes depicting attrition status (yes/no) jointly at post-medication session visits (Bernoulli function). First, we will examine attrition by group assignment. Here, the HLM will identify both the main effect of group assignment on later attrition as well as the potential for a group-by-time attrition effect. Second, group equivalency on factors potentially predictive of future drinking will be evaluated with t-tests using baseline drinking intensity and other characteristics as dependent measures.

3.3.12.2 Aim 1 (to characterize the acute effects of PO psilocybin 25 mg/70kg, 30mg/70kg, and 40 mg/kg in alcohol dependent patients)

3.3.12.2.1 Outcome measures

The primary safety measures will be changes in vital signs, psychological or other clinically significant adverse events, and serious adverse events using the FDA definition. The primary measure of hallucinogen effect is the 5-Dimensional Altered States of Consciousness Scale (5D-ASC) [130]. Secondary outcomes the Intensity score and other subscales from the Hallucinogen Rating Scale (HRS) [157], the Mysticism scale [132], the Mystical Experience Questionnaire [97], abuse potential visual analog scales, and within-session Monitor Rating Scale Scores.

3.3.12.2.2 Method

Tolerability. Adverse events will be tabulated. The primary safety measures will be changes in vital signs, psychological or other clinically significant adverse events, and serious adverse events using the FDA definition. Blood pressure and ratings will be contrasted by group using t-tests. AEs will be MedRA coded and groups contrasted using Chi squared or Fisher's exact test. All tests will be unprotected against inflated Type I error thus providing the most liberal estimate for potential safety concerns.

Psychoactivity. Scores from the HRS and other scales (mean, SD, median, range) will be computed and reported. Scores will be contrasted by group using t-tests for independent samples. Mean scores will be compared descriptively to means reported in other studies, particularly Griffiths et al. [97, 110].

3.3.12.3 Aim 2 (To evaluate the effect of psilocybin treatment on drinking outcomes for 32 weeks after the first administration, relative to diphenhydramine control)

3.3.12.3.1 Outcome measures

The primary drinking outcome for hypothesis 2 is percent heavy drinking days during weeks 5-36 (the 32 weeks following the first drug administration session) as assessed by the Time-line Follow-back [100]. Other drinking variables will serve as secondary outcomes, including percent days abstinent, drinks per

drinking day, days to first drinking day, days to first heavy drinking day, and consequences of drinking (from the Short Inventory of Problems [101]).

3.3.12.3.2 Method

For PHDD we will employ a MMRM including fixed, categorical effects of treatment, assessment, and site; site-by-treatment and treatment-by-assessment interactions; the continuous, a fixed covariate (week 1-4 PHDD); and using monthly values of PHDD (weeks 5-8, 9-12, 13-16, 17-20, 21-24, 25-28, 29-32, 32-36) as the dependent measure. Site and the site-by-treatment interaction will be removed from the final model if not significant at the $p < 0.05$ level. Secondary drinking measures with a continuous scale of measurement, i.e., PDA and DPDD, will be tested in a similar fashion and for days to first drinking day and days to first heavy drinking day (5 or more standard drinks for a man, 4 or more standard drinks for a woman). Cox survival analysis will be used. Measures of effect size will be calculated for all planned between-group contrasts (Hedges d) and the Q statistic will be calculated to assess the homogeneity of effect across the primary and secondary outcome measures of drinking.

3.3.12.4 <u>Aim 3 (To test whether or not characteristics of the drug administration session experiences mediate effects of psilocybin on short term (1 week) persisting effects and post-session drinking behavior).</u>

3.3.12.4.1 Measures

The three main dimensions of the 5D-ASC ("oceanic boundlessness," "anxious ego-disintegration," "visionary restructuralization,") and the total score from the MEQ will be used as independent measures of acute drug effects. Short term (1-week post-session) effects will be the as follows: for craving, the primary measure is the total score from The Penn Alcohol Craving Scale [148]; for self-efficacy, Abstinence Confidence from the Alcohol Abstinence Self-efficacy Scale [143]); and for motivation the "readiness" score from the Readiness Rulers [142]. Drinking outcomes will be those specified for Specific Aim 2.

3.3.12.4.2 Method

Tests of statistical mediation will use a product-of-coefficients approach with a bootstrapping procedure. This choice provides the most powerful test of mediation with smaller samples and does not assume normality in the distribution of (a'b') product terms (Preacher and Hayes, 2008; Hayes, 2009). HLM will be applied to identify the a and b parameter estimates for the between-group contrast (treatment assignment psilocybin versus control; df = 1), and baseline values of the dependent variable will be entered in level 2.

3.3.12.5 <u>Aim 4 (To evaluate the explanatory value of changes in alcohol craving, self-efficacy, motivation, and other psychological domains in accounting for the observed experimental effect of psilocybin relative to diphenhydramine control)</u>

3.3.12.5.1 *Measures*

For craving, the primary measure is the total score from The Penn Alcohol Craving Scale [148]; for self-efficacy, Abstinence Confidence from the Alcohol Abstinence Self-efficacy Scale [143]); and for motivation the "readiness" score from the Readiness Rulers [142]. Additional secondary measures are defined for depression (Hamilton Depression Scale score [152]) and anxiety (Hamilton Anxiety Scale score). Drinking outcomes will be those specified for Specific Aim 2.

3.3.12.5.2 Method

Statistical procedures described for Aim 3 will be used to determine if the proposed mediators account for the effectiveness of the psilocybin administration. Changes in proposed primary and secondary mediators will be assessed at weeks 5 and 9 (1 week following drug administration) and drinking outcome (PHDD) will be assessed at weeks 8 and 12 (4 weeks post drug administration). No protection for inflated Type I error will be applied to the testing of the three primary mediators, i.e., Penn Alcohol Craving Scale, and Abstinence Self-efficacy from the AASE, and Readiness from the Readiness Rulers. Performance site and the site by treatment group interaction terms will not be entered into the model.

3.3.12.6 Aim 5 (To evaluate pre-post changes in drinking in participants after they receive psilocybin in the third session)

3.3.12.6.1 Measures

The primary drinking outcome for hypothesis 5 is percent heavy drinking days during weeks 39-54 (the 32 weeks following the first drug administration session) as assessed by the Time-line Follow-back [100]. Other drinking variables will serve as secondary outcomes, including percent days abstinent, drinks per drinking day, days to first drinking day, days to first heavy drinking day, and consequences of drinking (from the Short Inventory of Problems [101]).

3.3.12.6.2 Method

Methods for Aim 5 will parallel those used in Aim 2, except that only participants in the control group will be included in this analysis, so treatment and site-by-treatment interactions will be omitted from the model, with the time effect being of principal interest. PHDD will be centered at week 42.

3.3.12.7 Statistical power

Power analysis is based on the primary outcome analysis contrasting the psilocybin group with the control group with respect to percent heavy drinking days in months 1-8 post pharmacologic treatment (Aim 2). With a total sample size of n = 180 (90 assigned to psilocybin and 90 to control), and 5% attrition (realistic because useable data require only 1 month follow-up in participants who have already participated for 1 month, N = 171) our power analysis was based on the assumption that four predictors would be in the regression-based HLM model. We estimate that the study has power of 0.8 to detect an effect of size f^2 = .065, with alpha = 0.05 (2-tailed) and no correction for multiple comparisons. This effect is comparable to that of naltrexone [9, 158, 159], and smaller than those observed in some recent trials of other promising medications [160-162]. Regardless of the statistical significance of these changes, standardized and non-standardized effect sizes will be computed for primary and secondary outcome variables. The addition of an open-label optional third dosing does not affect the required sample size for Aim 2 (primary outcome), but approximately doubles the sample size for Aim 1 and allows for the addition of Aim 5.

3.3.12.8 Ancillary analyses

Additional ancillary interim analyses will be performed on blinded, de-identified data by Ms. Gabrielle Agin-Liebes as part of her doctoral dissertation. Ms. Agin-Liebes is completing her training toward a PhD in clinical psychology at Palo Alto University (PAU), and is a member of PAU's Early Intervention Clinic clinical research laboratory, which provides and evaluates evidence-based treatments to prevent trauma-related problems in recently traumatized individuals. As part of this research laboratory, Ms. Agin-Liebes is examining the effects of trait self-compassion on trauma-related guilt cognitions. Blinded, de-identified scores on the SCQ, Self-Compassion Scale Short-Form (SCS-SF), and number of self-reported drinking days will be shared with Ms. Agin-Liebes to allow her to assess the following aims:

Aim 1) To examine the relationships between self-reported drinking days and self-compassion in the context of a psilocybin-facilitated MET program following two drug administration sessions (either psilocybin or placebo), Aim 2a) To examine within-person, pre-post intervention changes in self-reported drinking days in the context of a medication-facilitated MET program, Aim 2b) To examine within-person, pre-post intervention changes in self-reported self-compassion in the context of a medication-facilitated MET program, Aim 2c) To examine whether these within-person, pre-post intervention changes in self-reported drinking days are greater in participants that score above the median on the SCQ than the than those with SCQ scores below the median score, Aim 2d) To examine whether these within-person, pre-post intervention changes in self-compassion are greater in the in participants that score above the median on the SCQ than the than those with SCQ scores below the median score, and Aim 3) To examine whether SCS-SF scores mediate the relationship between SCQ scores and drinking behavior.

3.3.13 Study Milestones

We propose to implement the study in three stages, with interim analyses and DSMB review after the first and second stages.

Stage 1: Following final protocol approval by all necessary authorities and procurement of necessary funding, staff will be trained, at least 4 pilot/training participants per site will be treated, and 20 participants will be randomized into the main trial across two sites (UNM and NYU, 10 per site). At the end of this stage, feasibility and tolerability will be assessed, and necessary protocol modifications may be made.

Stage 2: An additional 80 participants will be randomized, with the possible addition of one or two additional sites to enhance recruitment capacity. At the end of this stage, interim checks of attrition rates and the assumed error standard deviation σ for the primary outcome measure will be conducted to assess the adequacy of the projected study sample size. This check will be performed by an unblinded statistician, who will estimate the primary model, and report back on error variance and drop-out rates. Additional necessary protocol modifications may be made, and final sample size will be determined. In addition, safety interim looks will be performed (without formal statistical testing) at the regular DSMB meetings or unscheduled times per the DSMB's request. Although at this time we are not planning a formal statistical interim analysis for efficacy or futility, such an interim analysis can be performed if requested by the DSMB or the sponsor.

Stage 3: Assuming 180 participants are required, the 80 remaining participants will be randomized, with an additional 9 months to complete treatment and assessment of the final participants.

3.3.14 Regulatory Considerations

3.3.14.1 Statement of Compliance

This trial will be conducted in compliance with the appropriate protocol, current Good Clinical Practice (GCP), the principles of the Declaration of Helsinki, and all other applicable regulatory requirements. Participating sites must obtain written approval of the study protocol, informed consent form, other supporting documents, and any advertising for participant recruitment from their local institutional review board (IRB) in order to participate in the study. Prior to study initiation, the protocol and the informed consent documents will be reviewed and approved by an appropriate Ethics Review Committee (ERC) or IRB. Any amendments to the protocol or consent materials must be approved before they are implemented. Annual progress reports and local Serious Adverse Event (SAE) reports will be submitted to each IRB, according to its usual procedures.

3.3.14.2 Necessary approvals

Human trials with Schedule I substances pose special challenges. The UNM site has already obtained an IND to study the use of psilocybin in the treatment of alcohol dependence, and has Schedule 1 DEA license and board of pharmacy approval to store, dispense, and administer psilocybin at UNM. The NYU site has an active Schedule I license to dispense psilocybin in the treatment of anxiety associated with cancer and will amend their Schedule 1 license for the purposes of this trial. The trial will not begin until the following have been achieved. 1) The existing UNM IND (#113080) will be amended to include the new protocol and the NYU site; 2) The existing DEA Schedule 1 licenses will be amended to include the new protocol; 3) any necessary local requirements, including Board of Pharmacy Schedule 1 permits, if necessary, will be obtained. Any additional sites will also satisfy these requirements prior to beginning participation in the study.

3.3.14.3 Regulatory Files

The regulatory files should contain all required regulatory documents, study-specific documents, and all important communications. Regulatory files will be checked at each participating site for the regulatory documents compliance prior to study initiation, throughout the study, as well as at the study closure.

3.3.14.4 Health Insurance Portability and Accountability Act (HIPAA)

Study sites may be required by their institutions to obtain authorization from participants for use of protected health information. Sites will be responsible for communicating with their IRBs or Privacy Boards and obtaining the appropriate approvals or waivers to be in regulatory compliance.

3.3.14.5 Financial Disclosure

All investigators will comply with the requirements of 42 CFR Part 50, Subpart F to ensure that the design, conduct, and reporting of the research will not be biased by any conflicting financial interest. Everyone with decision-making responsibilities regarding the protocol will have an up-to-date signed financial disclosure form on file.

3.3.14.6 Clinical Monitoring

Site staff will be required to audit source documentation, including informed consent forms and HIPAA forms, regulatory documents and case report forms on a biannual basis. Site staff will be responsible for local quality assurance and will verify that study procedures are properly followed and that site staff are trained and able to conduct the protocol appropriately. If the site staff's review of study documentation indicates that additional training of study personnel is needed, this will be arranged as per the PI. Study team members will review each other's data for completeness, accuracy, and fidelity to the protocol.

3.3.14.7 Study Documentation

Study documentation includes all case report forms, data correction forms, workbooks, source documents, monitoring logs and appointment schedules, sponsor-investigator correspondence, and signed protocol and amendments, Ethics Review Committee or Institutional Review Board correspondence, approved consent form, and signed participant consent forms. Source documents include all recordings of observations or notations of clinical activities and all reports and records necessary for the evaluation and reconstruction of the clinical research study. Whenever possible, the original recording of an observation should be retained as the source document; however, a photocopy is acceptable provided that it is a clear, legible, and an exact duplication of the original document.

3.3.15 Compensation to Participants

Participants will be reimbursed $50 for the screening visit, baseline visit, and each comprehensive visit (weeks 4, 8, 12, 24, 36, 38, 42 and 54), and $20 for the interim visits 1 week after each drug administration session (weeks 5, 9 and 39). Participants completing all of the assessments would therefore receive a total of $560.

4. HUMAN SUBJECTS PROTECTIONS

4.1 Risks to Human Subjects

4.1.1 Human Subjects Involvement and Characteristics.

4.1.1.1 Participants

Subjects will be women and men, age 25 to 65 with DSM-IV alcohol dependence, recruited from the community through advertisements at 2-4 sites. Participants will meet the inclusion and exclusion criteria listed in Section 3.3.6 of the protocol. Recruitment will continue until up to approximately 180 participants are randomized.

4.1.1.2 Study treatments

The proposed investigation is a multi-site, double-blind active-controlled trial (n = 180, 90 per group) contrasting the acute and persisting effects of psilocybin to those of diphenhydramine in the context of outpatient alcoholism treatment. The total duration of psychosocial treatment in the double blind period will be 12 weeks, and double-blind drug-administration sessions will occur after 4 and 8 weeks. The first psilocybin session will employ a moderately high dose of psilocybin (25 mg/70 kg). The dose may be increased in the second session to 30 mg/70 kg or 40 mg/70 kg, depending on response in prior sessions. The dose of diphenhydramine will start at 50 mg, and may be increased to 100 mg in the second session if 50 mg is well tolerated in the first session. Following completion of the double-blind period (34 weeks after randomization) all participants who meet interim safety criteria will be offered an additional session in which psilocybin will be administered. Drug administration will occur during 8-hour sessions conducted in an outpatient setting under close medical and psychiatric monitoring by two trained monitors. Psychosocial treatment will occur in the context of a behavioral intervention incorporating a modified version of the

Motivational Enhancement Therapy (Called Motivational Enhancement and Taking Action, META) [103] as well as preparation before and debriefing after the psilocybin session.

Procedures for minimizing COVID-19 risk among participants and study team members during required in-person visits (including routine temperature checks, use of personal protective equipment, symptom checks, etc.) are outlined in the COVID Safety Procedures SOP (etc-research-01\EtOH-14-00614\4. Study Documentation\3. SOPs\ SOP_14-00614_COVIDSafety_Procedures_V2_09.01.2020.docx).

4.1.2 Sources of Materials

Materials obtained from participants will include samples of blood, urine, hair, fingernails, and breath. Data will be collected through use of interviews, questionnaires, physical examination, and laboratory tests. These data will include measures of medical health (medical evaluation), alcohol withdrawal, psychiatric diagnosis including drug and alcohol use disorders, substance use, alcohol consequences, motivation, self-efficacy, craving, mood and anxiety, acute effects of psilocybin administration, persisting effects of psilocybin administration, and safety. These materials and data will be obtained specifically for research purposes

4.1.3 Potential Risks

4.1.3.1 Risks of experimental medications

4.1.3.1.1 Psilocybin

The test article used in the proposed study is psilocybin. In this study psilocybin will be used as the putatively active treatment. Psilocybin is a Schedule 1 substance with no currently accepted medical use. However, it has been used safely in numerous previous human studies, both in the US and abroad. In carefully conducted clinical research settings, with careful screening, preparation and support, the risks of psilocybin administration appear to be low [40, 41, 43, 97, 105]. Classic hallucinogens including psilocybin have the lowest physiological toxicity of all well-known drugs of abuse [163]. A recent analysis by the Independent Scientific Committee on Drugs in the UK found that individual and societal harms associated with use of psilocybin mushrooms were the lowest of any of the 20 drugs assessed [6]. However, there are some rare but potentially serious effects that require attention. With each exposure to psilocybin there is an additional chance that study participants may experience adverse events.

- Experiences with hallucinogens provoke a variety of positive and negative emotional responses in the hours to weeks following the acute drug experience, which can be unsettling. Anxiety and dysphoria are common during psilocybin intoxication. These symptoms can be distressing, and were the predominant emotional state in 4/36 sessions at 0.43 mg/kg reported by Griffiths et al. (2006). These symptoms generally respond well to reassurance. Anxiety rarely can escalate to panic.
- Increased blood pressure and heart rate during intoxication (mean increase of approximately 20 mm Hg systolic, 12 mm Hg diastolic, mean increase in pulse of approximately 10 bpm in Griffiths et al., 2006). This side effect is common, but unlikely to be clinically significant with careful screening for hypertension and cardiovascular disease.
- Serotonin syndrome is possible if psilocybin is used in combination with other serotonergic drugs. Individuals known to be taking such drugs will be excluded from the study. This adverse event is expected to be very uncommon.
- Transient psychotic symptoms (delusions, paranoia, hallucinations) have been reported during psilocybin intoxication. We are unaware of any reports of persistent psychotic symptoms associated with the use of psilocybin, but such symptoms have been reported following use of LSD [43, 164]. Individuals with history of psychosis or vulnerability (e.g., family history) are thought to be at higher risk for psychotic reactions to hallucinogens, but we cannot rule out a very small risk of such reactions to psilocybin in normal individuals.
- Secondary analyses of a pharmacokinetic study [165] revealed an association between psilocybin level increase in QTc (See Investigator Brochure v2.). at psilocin concentrations above 31.1 ng/ml.
- Hallucinogen persisting perception disorder can be caused by hallucinogen use. The incidence of this disorder is thought to be very low, and associated primarily with use of LSD [47].

- Headaches are common following psilocybin administration [109]. These headaches are not severe, resolve within a day, and appear to respond to over-the-counter pain medications.
- Psilocybin, like other classic hallucinogens, is not reliably reinforcing in humans or in animal models used to study addiction potential [46]. However, psilocybin is an abusable drug. It is theoretically possible that a participant could develop a pattern of psilocybin misuse due to his/her experience in the trial, although this has not been reported in previous studies in which psilocybin or other classic hallucinogens have been administered to humans.

Although there is considerable clinical experience with psilocybin over more than 50 years, it has not been used in controlled trials with alcohol dependent subjects. There is, however, a large literature dating from the 1950s through the early 1970s, including over 1000 subjects, on the use of LSD in the treatment of alcoholism [1, 11-15, 21-26]. Few safety issues were noted in these studies.

4.1.3.1.2 Diphenhydramine

Diphenhydramine is used as an active control medication in this study. Diphenhydramine is a H1 antagonist (antihistamine) which is available over the counter and by prescription for treatment of insomnia, allergic reactions, parkinsonism, and motion sickness. It has been safely administered in doses up to 400 mg PO [114-116, 166]. Reported side effects and adverse events associated with diphenhydramine include the following (from package insert [167]).

- *General:* Urticaria, drug rash, anaphylactic shock, photosensitivity, excessive perspiration, chills, dryness of mouth, nose and throat.
- *Cardiovascular System:* Hypotension, headache, palpitations, tachycardia, extrasystoles.
- *Hematologic System:* Hemolytic anemia, thrombocytopenia, agranulocytosis.
- *Nervous System:* Sedation, sleepiness, dizziness, disturbed coordination, fatigue, confusion, restlessness, excitation, nervousness, tremor, irritability, insomnia, euphoria, paresthesia, blurred vision, diplopia, vertigo, tinnitus, acute labyrinthitis, neuritis, convulsions.
- *GI System:* Epigastric distress, anorexia, nausea, vomiting, diarrhea, constipation.
- *GU System:* Urinary frequency, difficult urination, urinary retention, early menses.
- *Respiratory System:* Thickening of bronchial secretions, tightness of chest and wheezing, nasal stuffiness.

4.1.3.1.3 Adjunctive medications

With the exception of acetaminophen and ibuprofen, use of these medications is expected to occur uncommonly if at all in this study.

- For treatment of severe hypertension: captopril 25mg orally or sublingually; clonidine Orally, initial 0.1mg; may be followed by additional doses of 0.1 mg every hour, if necessary, to a maximum total dose of 0.7 mg. Hydralazine orally, 10mg once. Common side effects include headache, lightheadedness, dizziness, flushing, orthostatic hypotension, and reflex tachycardia. Uncommon but serious reactions include severe hypotension, paradoxical bradycardia angioedema and allergic reactions.
- For severe anxiety: Diazepam 5-10 mg orally or Lorazepam 1 mg intramuscularly. The most common effects of benzodiazepines are sedation, dizziness, weakness, and unsteadiness. Rare but serious adverse events that could occur with a single administration include respiratory depression, apnea, and anaphylaxis.
- Olanzapine 5-10 mg orally or intramuscularly for psychotic symptoms (delusions, hallucinations, disorganized behavior) that pose a significant danger. Common side effects of olanzapine possible with one or two doses are somnolence, dizziness, hypotension, akathisia, extrapyramidal symptoms, dry mouth, tremor, nausea, and vomiting. Rare but serious side effects include seizures, neuroleptic malignant syndrome and tardive dyskinesia, (the latter two are extremely unlikely with one or two small doses). The risk of hypotension is increased when intramuscular olanzapine is given in combination with benzodiazepines, so intramuscular olanzapine will not be given if diazepam has already been administered.

- For headache or pain: Acetominophen 500-1000 mg orally q4 hours prn pain/headache and/or Ibuprofen 600 mg-800 mg orally x q8 prn pain/headache. Common side effects include nausea, stomach discomfort.

4.1.3.1.4 Risk in pregnancy and lactation

Risks of psilocybin in pregnancy and lactation are unknown. Diphenhydramine is Pregnancy Category B Benzodiazepines are pregnancy category D, with evidence of fetal risk. Use during lactation is considered possibly unsafe. Olanzapine is pregnancy category C, not for use in nursing. Captopril is pregnancy category D, evidence of fetal toxicity. Hydralazine and clonidine are pregnancy category C.

4.1.3.2 Risks of psychosocial treatments

There are no known specific risks to META or the preparation and debriefing sessions used in the proposed trial. Non-specific risks include discomfort with sharing personal information, and the risk of therapist misconduct. These risks are judged to be very minor.

4.1.3.3 Risks of blood draws.

For most people, needle punctures for blood draws do not cause any serious problems. However, they may cause bleeding, bruising, discomfort, infections and/or pain at the needle site, or dizziness.

4.1.3.4 Risks of assessment procedures

There are no known psychological risks associated with the questionnaires used in the study, all of which have been used extensively in clinical populations. It is possible that discussion of substance use and psychiatric symptoms may cause emotional discomfort in some participants. To minimize such discomfort, the following steps will be taken. The consent form will fully inform the participants about the nature of the information to be disclosed in the protocol, and the participants will be informed in the consent form that they can refuse to answer any questions or withdraw from the study at any time. Participants will be informed that all information is confidential. The steps taken to guard confidentiality, as well as the limits to confidentiality, will be described. One of the investigators of the project will be available to meet with any participant who becomes distressed about any aspect of the protocol and wishes to discuss this.

4.1.3.5 Risks to confidentiality and potential legal consequences.

Information from this study may be submitted to the U.S. Food and Drug Administration (FDA). Medical records which identify subjects and the consent form signed by subjects may be inspected by the FDA and the local Institutional Review Board. Because of the need to release information to these parties, absolute confidentiality cannot be guaranteed. The results of this research project may be presented at meetings or in publications. However, the identity of individual subjects will not be disclosed in those presentations. Additionally, with the participant's permission, their information may be disclosed to a contact such as a friend or family member that they identify in a written release specifying what information may be shared with whom.

4.1.3.6 Risks and benefits of alternative treatments.

Standard of care for outpatient treatment of alcohol dependence is pharmacotherapy with medication monitoring and/or evidence-based psychosocial treatment (individual and/or group therapy). The FDA-approved treatments for alcohol dependence are disulfiram (Antabuse), naltrexone (ReVia and Vivitrol) and acamprosate (Campral). Common side effects of disulfiram include the disulfiram-alcohol reaction, rash, peripheral neuropathy, drowsiness, impotence, headache, acne, and metallic taste. Serious reactions include respiratory depression, cardiovascular collapse, arrhythmias, acute MI, congestive heart failure, seizures, coma, psychosis, optic neuritis, and hepatitis. All but the last three are potential consequences of a severe disulfiram-alcohol reaction. Common side effects of naltrexone include insomnia, nausea, vomiting, anxiety, headache, abdominal pain, muscle aches, rash, dizziness, fatigue, somnolence, anorexia, constipation, liver dysfunction, and chills. Serious reactions include suicidality, depression, precipitated opiate withdrawal syndrome, hypersensitivity reaction, and hepatotoxicity. Common side effects of acamprosate include diarrhea, insomnia, anxiety, depression, asthenia, nausea, pruritus, and dizziness. Serious reactions include suicidality and depression. Evidence-based psychosocial treatments for alcohol dependence include methods based on motivational interviewing (such as the META used in this study), cognitive, behavioral, and cognitive-

behavioral approaches, 12-step facilitation therapies, approaches involving the spouse and family, and standardized counseling approaches incorporating elements of these various approaches.

4.2 Adequacy of Protection Against Risks

4.2.1 Recruitment and Informed Consent

4.2.1.1 Recruitment

Patients will be recruited from the community through advertisements in local media.

4.2.1.2 Pre-Screening

An IRB-approved pre-screening form will be used by study staff to pre-screen individuals expressing interest in the study, to assess whether they are likely to qualify for the study. Interested participants who pass the pre-screening will be referred for informed consent. No PHI data will be kept unless subjects sign the informed consent form. PHI data of individuals who do not sign an informed consent form will be destroyed. The only information that will be retained from the pre-screening form for individuals that do not pass the pre-screening is the reason they were excluded, the date of the pre-screening, and where they learned about the study.

4.2.1.3 Informed consent process and document

Interested patients will be provided with an informed consent form, approved by the local site's Institutional Review Board and including all pertinent details of the study including description of the following: the assessment interview and questionnaires; the follow-up interviews; description of experimental treatment; risks and benefits of study procedures; alternatives to participation in the study; confidentiality; emergency treatment and compensation for injury; payment for participation; a statement that patients will be informed of any new findings affecting the risks or benefits of the study; a statement that participation is voluntary and that the patient may withdraw at any time; and information about whom to contact with questions or in case of emergency. The consent form will also include a section that allows participants to decide if they would like to participate in any potential future studies related to this study. The consent form will also include assurances of confidentiality and a statement that participation is entirely voluntary, that the decision to participate will in no way influence other aspects of the patient's treatment or involvement in the main trial, and that the participant is free to withdraw participation at any time. Prior to signing the informed consent document, patients will be required to pass an informed consent quiz (10 items, passing score 90% or above) covering key elements of the study. Patients who score less than 90% on the informed consent quiz will be counseled further and will have up to a total of 3 chances to pass the quiz. Patients indicate their consent to participate in the study by signing and returning the informed consent form.

4.2.2 Protection Against Risk

4.2.2.1 Management of risk due to study treatments

Risks of hallucinogen administration are thought to be minimized by a) careful screening of participants, b) extensive preparation, c) establishment of rapport and trust between participants and monitors, d) presence of two monitors during the psilocybin sessions, e) a safe and reassuring physical environment, f) careful planning for unusual but potentially serious adverse reactions, and g) debriefing and follow-up for possible residual adverse effects [105]. These principles are incorporated into our protocol as follows.

a. Subject selection: Exclusion criteria are designed to exclude patients who would be at elevated risk for adverse events due to psilocybin. These criteria are listed in Section 3 above, and include both medical exclusions and psychiatric exclusions for serious psychopathology, history of violent or suicidal behavior, and family history of psychosis or suicide.

b. Subject preparation: In addition to the information and discussion provided in the informed consent process, participants will spend a total of 6 hours in 4 sessions: two 1-hour META sessions, and two preparation sessions (4 hours total) including both the PSI therapist and the META therapist. Both of the clinicians will be present during the psilocybin sessions. Preparation will include 1) open-ended questions to establish rapport, learn about the participant's history, belief system, and values, assess motivation and expectations for the

study, and discuss any previous experience with hallucinogens; 2) detailed information about the physiological and psychological effects of psilocybin; 3) emphasis that the purpose of the psilocybin sessions is to facilitate behavior change, and discussion of how this could work; 4) advice as to how to deal with dysphoric reactions to psilocybin, should they occur; 5) identification of any personally meaningful items that the participant will bring to the session (e.g., photographs, images, objects of personal or religious significance); and 6) discussion of ground rules for the session.

c. Establishment of rapport and trust: The extensive contact between the participant and therapists prior to the psilocybin sessions is intended to build rapport and trust during the psilocybin sessions.

d. Support during psilocybin sessions: Because this study involves use of psilocybin in a clinical population as part of an intervention intended to be therapeutic, the two study therapists will serve as the monitors who will attend the patient during the psilocybin sessions. At least one of these therapists will be a licensed clinician.

e. Physical environment: The psilocybin session will take place in outpatient facilities that are appropriate for hallucinogen administration sessions. To the extent possible, the room used for the session will be specially prepared for the sessions to provide a warm and home-like rather than a stark clinical quality because of the large influence that setting can have on the subjective effects of hallucinogens. Each site will have detailed SOPs in place for managing medical or psychiatric emergencies should any occur.

f. Planning for emergencies: Each of the research sites has established practices for managing medical and psychiatric emergencies, and the study staff will continue to utilize these procedures. Staff will be responsible for monitoring participants for possible clinical deterioration or other problems, and for implementing appropriate courses of action. Acute adverse reactions to psilocybin initially will be managed by increasing psychological support (“talking down”). Medications will be available for administration if needed to treat hypertension (sublingual captopril 25 mg orally/sublingually, clonidine 0.1 mg PO, and hydralazine 10 mg PO), anxiety (diazepam 5-10 mg PO, or lorazepam 1 mg IM), or acute psychosis posing a danger to the participant or others (olanzapine 5-10 mg PO or IM). Referral for emergency treatment and/or hospitalization will be available at each site. A study clinician will be available by pager or phone at all times during study participation.

g. Debriefing and Follow-up: Debriefing sessions, approximately 2 hours long and conducted by the PSI therapist and the META therapist on the day after the psilocybin sessions, consist of the following elements: 1) open-ended inquiry concerning the psilocybin session (and what has happened since); 2) invitation to reflect on the experience; 3) discussion of what has changed as a result of the session; 4) discussion of how the session has affected the participant’s relationship to alcohol; and 5) assessment of mental status. Additional META sessions and booster sessions (two 1-hour sessions following the first debriefing session, and three 1-hour sessions following the second debriefing session) will allow further opportunity to process the experience.

4.2.2.2 Minimization of risk to confidentiality

Confidentiality of research material will be ensured by storing the research materials in locked cabinets in NYU space within the A, C-D, and H buildings of Bellevue Hospital Center. Material will be available only to project staff, and only as needed. All project staff will be thoroughly trained in issues relating to confidentiality. Participants will be identified in case report forms (CRFs) by initials and an identification code. Data will be entered into REDCap™, a program designed specifically to protect patient privacy and confidentiality (see section 3.3.11). Published reports will be based on group data; no individual data will be reported. As a further protection to confidentiality, we will request a Confidentiality Certificate from the Department of Health and Human Services (DHHS). With this Certificate, the investigators cannot be forced (for example by court order or subpoena) to disclose research information that may identify individual patients in any Federal, State, or local civil, criminal, administrative, legislative, or other proceedings. Disclosure will be necessary, however, upon request of DHHS for audit or program evaluation purposes. DHHS ensures confidentiality of requested data. Participants will be notified in the informed consent that the Certificate of Confidentiality does not prevent them or a member of their family from voluntarily releasing information about themselves and their involvement in the research. If an insurer, employer or other person obtains their written consent to receive research information, then the researcher may not use the Certificate to withhold that information. Participants will also

be informed that the Certificate of Confidentiality does not prevent the researchers from disclosing voluntarily, without their consent, information that would identify them as a participant of the research project under the following circumstances: the present danger of child abuse, suicide, and/or homicide. In addition to current measures, the present study will comply with the Health Insurance Portability and Accountability Act of 1996. Records related to this study will not be released to third parties.

4.3 Potential Benefits of the Proposed Research to Human Subjects and Others

4.3.1 Potential Benefits to Participants

Participants may or may not experience clinical benefit from this study. There is equipoise regarding the effects of the experimental drug used in this study. Aspects of study participation likely to be beneficial include free medical and psychiatric evaluations, the attention and support or of participating in a clinical trial, and a course of an evidence-based psychosocial treatment for alcohol dependence.

4.3.2 Potential Benefits to Others.

Knowledge gained through this study may aid the development of more effective treatments for individuals with alcohol dependence and other addictive disorders.

4.3.3 Risk Benefit Assessment

Risks to individual participants appear to be balanced by the likely benefits of study participation, and outweighed by the potential benefits to others.

4.4 Importance of the Knowledge to be Gained

There is an urgent need to develop more effective methods to help people who suffer from addictions and other forms of loss of behavioral control (e.g., overeating). The knowledge gained through this study could point the way to an entirely new treatment for alcohol dependence, and may also yield important gains in understanding of the psychological processes that underlie behavior change. As indicated above, these potential benefits (in addition to the potential benefits to individual participants) appear to justify the risks to individual participants.

4.5 Data and Safety Monitoring Plan

4.5.1 General Considerations

Although the safety profile of psilocybin in clinical research is well established, our pilot study is the only controlled trial to date using psilocybin in an alcohol dependent population (although there are more than thirty published studies reporting on the use of LSD in the treatment of alcohol dependence). The data and safety monitoring plan is therefore designed both to ensure that the risks of medications and study-related procedures are minimized for patients, and to minimize any doubt that there are adequate safeguards in place to minimize this risk.

4.5.2 Institutional Review Board (IRB)

All study procedures will be approved by the local IRB at each participating site.

4.5.3 Data and Safety Monitoring Board

A Data and Safety Monitoring Board (DSMB) will be established, comprising three to five individuals appointed by the Heffter Research Institute. This committee will meet (in person or by teleconference) prior to enrollment of the first participant in the double blind and at least annually thereafter, including meetings after enrollment of the first 10 participants, and upon completion of stage 1 and stage 2 of the trial. At its meetings the Board will

- review protocol violations and deviations to assess adequacy of study
- ensure documentation of informed consent
- review enrollment and retention
- review IND information
- discuss investigator or key personnel changes

- review completeness and quality of data collection forms
- evaluate the aggregate analysis of adverse events/serious adverse events
- review confidentiality
- review interim analyses conducted at completion of stage 1 and stage 2 of the trial.

Following each meeting, the board will provide the PI with a report including a recommendation to continue the study unchanged, continue with modifications of the protocol and/or the consent form to protect participant safety, or terminate the study.

4.5.4 Procedures for Monitoring and Reporting Adverse Events.

The study staff will be trained to monitor for and report adverse events and Serious Adverse Events. Adverse events (AEs), when present, will be collected on an AE case report form at the end of the first psilocybin session and at all subsequent visits. The form will include an assessment of clinical significance and study relatedness. Serious Adverse Events (SAEs) will be documented on a separate SAE form. AEs will be reported in accordance with federal law and policies the IRB requirements. Reporting procedures vary depending on the severity of the AE.

4.5.4.1 Safety reporting to the FDA

Safety reporting will follow 21CFR312.32. Site investigators will notify the Lead Investigator of any SAEs within 24 hours of becoming aware of the event. The Sponsor will notify the FDA regarding any suspected adverse reaction associated with the use of the drug that is both serious and unexpected. The sponsor will also notify the FDA of findings from other human, animal, or in vitro studies that suggest increased risk to humans, or any pattern of increase in the rate of adverse events over what is expected. Each notification will be made as soon as possible and no greater than 15 calendar days after the initial receipt of the information. Each written notification will be submitted on an FDA Form 3500A. The Principal Investigator will also notify the FDA of any unexpected fatal or life-threatening experience associated with the use of the drug as soon as possible but no later than 7 calendar days after the initial receipt of the information.

4.5.4.2 Other reporting to FDA

On an annual basis as part of the update to the study IND, the Principal Investigator will submit to the FDA:

A summary of all IND safety reports submitted during the past year.

A list of all subjects who died during the participation in the investigation, listing cause of death for each.

A list of subjects who dropped out during the course of the investigation in association with any adverse experience, whether or not thought to be drug related.

4.5.4.3 Stopping Rules for Study

Stopping rules will be put in place if 1) there are untoward and concerning levels of Adverse Event (AE) or Serious Adverse Event (SAE) outcomes attributable to psilocybin, or 2) there is a significant worsening of drinking behavior or consequences in the psilocybin group. The methodology that will be used for both of the 2 rules above will be as follows: interim analyses will be performed in the aggregate (psilocybin and control groups combined) for both AEs/SAEs and drinking clinical outcomes, and these results will be reviewed by the DSMB. If the aggregate analyses reveal either concerning levels of AEs/SAEs or worsening drinking outcomes, then an unblinded statistician will analyze the data to determine (as best as possible from the data) if the concerning AEs/SAEs and/or the worsening drinking outcomes are significantly due to the psilocybin group compared to the placebo. If the DSMB finds it is likely the psilocybin group is contributing to negative outcomes, they will consider solutions including protocol changes or potentially stopping the study.

5. REFERENCES

1. Krebs, T.S. and P.O. Johansen, *Lysergic acid diethylamide (LSD) for alcoholism: meta-analysis of randomized controlled trials.* J Psychopharmacol, 2012.
2. Bogenschutz, M.P., *Effects of psilocybin in the treatment of addictions: a review and preliminary results from two ongoing trials.* Neuropsychopharmacology, 2012. **38**: p. S15-S16.
3. Bogenschutz, M.P., *Studying the Effects of Classic Hallucinogens in the Treatment of Addictions: Rationale, Methodology, and Current Research with Psilocybin.* Current Drug Abuse Reviews, In press.
4. Hasin, D.S., et al., *Prevalence, correlates, disability, and comorbidity of DSM-IV alcohol abuse and dependence in the United States: results from the National Epidemiologic Survey on Alcohol and Related Conditions.* Arch Gen Psychiatry, 2007. **64**(7): p. 830-42.
5. Rehm, J., et al., *Global burden of disease and injury and economic cost attributable to alcohol use and alcohol-use disorders.* Lancet, 2009. **373**(9682): p. 2223-33.
6. Nutt, D.J., L.A. King, and L.D. Phillips, *Drug harms in the UK: a multicriteria decision analysis.* Lancet, 2010. **376**(9752): p. 1558-65.
7. Volkow, N.D. and T.K. Li, *Drugs and alcohol: treating and preventing abuse, addiction and their medical consequences.* Pharmacol Ther, 2005. **108**(1): p. 3-17.
8. Berglund, M., *A better widget? Three lessons for improving addiction treatment from a meta-analytical study.* Addiction, 2005. **100**(6): p. 742-50.
9. Anton, R.F., et al., *Combined pharmacotherapies and behavioral interventions for alcohol dependence: the COMBINE study: a randomized controlled trial.* JAMA, 2006. **295**(17): p. 2003-17.
10. Fiellin, D.A., et al., *Counseling plus buprenorphine-naloxone maintenance therapy for opioid dependence.* N Engl J Med, 2006. **355**(4): p. 365-74.
11. Abuzzahab, F.S., Sr. and B.J. Anderson, *A review of LSD treatment in alcoholism.* Int Pharmacopsychiatry, 1971. **6**(4): p. 223-35.
12. Halpern, J.H., *The use of hallucinogens in the treatment of addiction.* Addiction Research, 1996. **4**(2): p. 177-189.
13. Mangini, M., *Treatment of alcoholism using psychedelic drugs: a review of the program of research.* J Psychoactive Drugs, 1998. **30**(4): p. 381-418.
14. Dyck, E., *'Hitting Highs at Rock Bottom': LSD Treatment for Alcoholism, 1950–1970.* Social History of Medicine, 2006. **19**(2): p. 313-329.
15. Grinspoon, L. and J.B. Balakar, *Psychedelic drugs reconsidered*. 1997, New York: The Lindesmith Center.
16. Ludwig, A.M. and J. Levine, *A Controlled Comparison of Five Brief Treatment Techniques Employing Lsd, Hypnosis, and Psychotherapy.* Am J Psychother, 1965. **19**: p. 417-35.
17. Savage, C. and O.L. McCabe, *Residential psychedelic (LSD) therapy for the narcotic addict. A controlled study.* Arch Gen Psychiatry, 1973. **28**(6): p. 808-14.
18. Grof, S., et al., *DPT as an adjunct in psychotherapy of alcoholics.* Int Pharmacopsychiatry, 1973. **8**(1): p. 104-15.

19. Rhead, J.C., et al., *Psychedelic drug (DPT)-assisted psychotherapy with alcoholics: a controlled study.* Journal of Psychedelic Drugs, 1977. **9**(4): p. 287-300.

20. Miller, W.R. and P.L. Wilbourne, *Mesa Grande: a methodological analysis of clinical trials of treatments for alcohol use disorders.* Addiction, 2002. **97**(3): p. 265-77.

21. Smart, R.G., et al., *A controlled study of lysergide in the treatment of alcoholism. 1. The effects on drinking behavior.* Q J Stud Alcohol, 1966. **27**(3): p. 469-82.

22. Hollister, L.E., J. Shelton, and G. Krieger, *A controlled comparison of lysergic acid diethylamide (LSD) and dextroamphetmine in alcoholics.* Am J Psychiatry, 1969. **125**(10): p. 1352-7.

23. Ludwig, A., et al., *A clinical study of LSD treatment in alcoholism.* Am J Psychiatry, 1969. **126**(1): p. 59-69.

24. Bowen, W.T., R.A. Soskin, and J.W. Chotlos, *Lysergic acid diethylamide as a variable in the hospital treatment of alcoholism: a follow-up study.* J Nerv Ment Dis, 1970. **150**(2): p. 111-8.

25. Pahnke, W.N., et al., *The experimental use of psychedelic (LSD) psychotherapy.* JAMA, 1970. **212**(11): p. 1856-63.

26. Tomsovic, M. and R.V. Edwards, *Lysergide treatment of schizophrenic and nonschizophrenic alcoholics: a controlled evaluation.* Q J Stud Alcohol, 1970. **31**(4): p. 932-49.

27. Maciulaitis, R., et al., *Ibogaine, an anti-addictive drug: pharmacology and time to go further in development. A narrative review.* Hum Exp Toxicol, 2008. **27**(3): p. 181-94.

28. Vollenweider, F.X. and M. Kometer, *The neurobiology of psychedelic drugs: implications for the treatment of mood disorders.* Nat Rev Neurosci, 2010. **11**(9): p. 642-51.

29. Krupitsky, E., et al., *The combination of psychedelic and aversive approaches in alcoholism treatment.* Alcoholism Treatment Quarterly, 1992. **9**: p. 99-105.

30. Krupitsky, E., et al., *Ketamine psychotherapy for heroin addiction: immediate effects and two-year follow-up.* J Subst Abuse Treat, 2002. **23**(4): p. 273-83.

31. Krupitsky, E.M., et al., *Single Versus Repeated Sessions of Ketamine-Assisted Psychotherapy for People with Heroin Dependence.* Journal of Psychoactive Drugs 2007. **39**(1): p. 13-19.

32. Kolp, E., et al., *Ketamine Enhanced Psychotherapy: Preliminary Clinical Observations on Its Effectiveness in Treating Alcoholism.* The Humanistic Psychologist, 2006. **34**(4): p. 399-422.

33. Price, R.B., et al., *Effects of Intravenous Ketamine on Explicit and Implicit Measures of Suicidality in Treatment-Resistant Depression.* Biol Psychiatry, 2009.

34. Diazgranados, N., et al., *A randomized add-on trial of an N-methyl-D-aspartate antagonist in treatment-resistant bipolar depression.* Arch Gen Psychiatry, 2010. **67**(8): p. 793-802.

35. Zarate, C.A., Jr., et al., *A randomized trial of an N-methyl-D-aspartate antagonist in treatment-resistant major depression.* Arch Gen Psychiatry, 2006. **63**(8): p. 856-64.

36. Mathew, S.J., et al., *Riluzole for relapse prevention following intravenous ketamine in treatment-resistant depression: a pilot randomized, placebo-controlled continuation trial.* Int J Neuropsychopharmacol, 2010. **13**(1): p. 71-82.

37. aan het Rot, M., et al., *Safety and efficacy of repeated-dose intravenous ketamine for treatment-resistant depression.* Biol Psychiatry, 2010. **67**(2): p. 139-45.

38. Berman, R.M., et al., *Antidepressant effects of ketamine in depressed patients.* Biol Psychiatry, 2000. **47**(4): p. 351-4.

39. Krystal, J.H., *Ketamine and the potential role for rapid-acting antidepressant medications.* Swiss Med Wkly, 2007. **137**(15-16): p. 215-6.

40. Moreno, F.A., et al., *Safety, tolerability, and efficacy of psilocybin in 9 patients with obsessive-compulsive disorder.* J Clin Psychiatry, 2006. **67**(11): p. 1735-40.

41. Grob, C.S., et al., *Pilot Study of Psilocybin Treatment for Anxiety in Patients With Advanced-Stage Cancer.* Arch Gen Psychiatry, 2010.

42. Johnson, M.W. and R.R. Griffiths, *Psilocybin in smoking cessation: a pilot study*, in *118th Annual Meeting of the American Psychological Association*. 2010: San Diego, CA.

43. Strassman, R.J., *Adverse reactions to psychedelic drugs. A review of the literature.* J Nerv Ment Dis, 1984. **172**(10): p. 577-95.

44. Wu, L.T., et al., *Hallucinogen use disorders among adult users of MDMA and other hallucinogens.* Am J Addict, 2008. **17**(5): p. 354-63.

45. Gillespie, N.A., et al., *Factor and item-response analysis DSM-IV criteria for abuse of and dependence on cannabis, cocaine, hallucinogens, sedatives, stimulants and opioids.* Addiction, 2007. **102**(6): p. 920-30.

46. Glennon, R.A., *Pharmacology of classical hallucinogens and related designer drugs*, in *Principles of Addiction Medicine, fourth edition*, R.K. Ries, et al., Editors. 2009, Lippincott, Williams, and Wilkins: Philadeophia, PA. p. 215-230.

47. Halpern, J.H. and H.G. Pope, Jr., *Hallucinogen persisting perception disorder: what do we know after 50 years?* Drug Alcohol Depend, 2003. **69**(2): p. 109-19.

48. Kunitz, S.J. and J.E. Levy, *Drinking careers: a twenty-five year study of three Navajo populations*. 1994, New Haven: Yale University Press.

49. Doering-Silveira, E., et al., *Report on psychoactive drug use among adolescents using ayahuasca within a religious context.* J Psychoactive Drugs, 2005. **37**(2): p. 141-4.

50. Fabregas, J.M., et al., *Assessment of addiction severity among ritual users of ayahuasca.* Drug Alcohol Depend, 2010. **111**(3): p. 257-61.

51. Halpern, J.H., et al., *Evidence of health and safety in American members of a religion who use a hallucinogenic sacrament.* Med Sci Monit, 2008. **14**(8): p. SR15-22.

52. Ross, S., *Serotonergic Hallucinogens and Emerging Targets for Addiction Pharmacotherapies.* Psychiatric Clinics of North America, 2012. **35**: p. 357-374.

53. Bogenschutz, M.P. and J.A. Pommy, *Therapeutic mechanisms of classic hallucinogens in the treatment of addictions: from indirect evidence to testable hypotheses.* Drug Testing and Analysis, In press.

54. Ray, T.S., *Psychedelics and the Human Receptorome.* PLoS ONE, 2010. **5**(2): p. e9019.

55. Nichols, D.E., *Hallucinogens.* Pharmacol Ther, 2004. **101**(2): p. 131-81.

56. Vollenweider, F.X., et al., *Psilocybin induces schizophrenia-like psychosis in humans via a serotonin-2 agonist action.* Neuroreport, 1998. **9**(17): p. 3897-902.

57. Puig, M.V., et al., *In vivo modulation of the activity of pyramidal neurons in the rat medial prefrontal cortex by 5-HT2A receptors: relationship to thalamocortical afferents.* Cereb. Cortex, 2003. **13**: p. 870-882.

58. Beique, J.C., et al., *Mechanism of the 5-hydroxytryptamine 2A receptor-mediated facilitation of synaptic activity in prefrontal cortex.* Proc. Natl Acad. Sci. USA 2007. **104**: p. 9870-9875.

59. Aghajanian, G.K. and G.J. Marek, *Serotonin, via 5-HT2A receptors, increases EPSCs in layer V pyramidal cells of prefrontal cortex by an asynchronous mode of glutamate release.* Brain Res, 1999. **825**(1-2): p. 161-71.

60. Zhang, C. and G.J. Marek, *AMPA receptor involvement in 5-hydroxytryptamine2A receptor-mediated pre-frontal cortical excitatory synaptic currents and DOI-induced head shakes.* Prog Neuropsychopharmacol Biol Psychiatry, 2008. **32**(1): p. 62-71.

61. Gonzalez-Maeso, J., et al., *Hallucinogens recruit specific cortical 5-HT(2A) receptor-mediated signaling pathways to affect behavior.* Neuron, 2007. **53**(3): p. 439-52.

62. Schmid, C.L. and L.M. Bohn, *Serotonin, but not N-methyltryptamines, activates the serotonin 2A receptor via a ss-arrestin2/Src/Akt signaling complex in vivo.* J Neurosci, 2010. **30**(40): p. 13513-24.

63. Schmid, C.L., K.M. Raehal, and L.M. Bohn, *Agonist-directed signaling of the serotonin 2A receptor depends on beta-arrestin-2 interactions in vivo.* Proc Natl Acad Sci U S A, 2008. **105**(3): p. 1079-84.

64. Moreno, J.L., et al., *Metabotropic glutamate mGlu2 receptor is necessary for the pharmacological and behavioral effects induced by hallucinogenic 5-HT2A receptor agonists.* Neurosci Lett, 2011. **493**(3): p. 76-9.

65. Buckholtz, N.S., et al., *Lysergic acid diethylamide (LSD) administration selectively downregulates serotonin2 receptors in rat brain.* Neuropsychopharmacology, 1990. **3**: p. 137-148.

66. Gresch, P.J., et al., *Behavioral tolerance to lysergic acid diethylamide is associated with reduced serotonin-2A receptor signaling in rat cortex.* Neuropsychopharmacology, 2005. **30**: p. 1693-1702.

67. Akash, K.G., et al., *Enhanced dopamine D2 receptor function in hypothalamus and corpus striatum: their role in liver, plasma and in vitro hepatocyte ALDH regulation in ethanol treated rats.* J Biomed Sci, 2008. **15**(5): p. 623-31.

68. Tsuchioka, M., et al., *Serotonin (5-HT) induces glial cell line-derived neurotrophic factor (GDNF) mRNA expression via the transactivation of fibroblast growth factor receptor 2 (FGFR2) in rat C6 glioma cells.* J Neurochem, 2008. **106**(1): p. 244-57.

69. Vaidya, V.A., et al., *5-HT2A receptor-mediated regulation of brain-derived neurotrophic factor mRNA in the hippocampus and the neocortex.* J Neurosci, 1997. **17**(8): p. 2785-95.

70. Ghitza, U.E., et al., *Role of BDNF and GDNF in drug reward and relapse: a review.* Neurosci Biobehav Rev, 2010. **35**(2): p. 157-71.

71. Jones, K.A., et al., *Rapid modulation of spine morphology by the 5-HT2A serotonin receptor through kalirin-7 signaling.* Proc Natl Acad Sci U S A, 2009. **106**(46): p. 19575-80.

72. Vollenweider, F.X., et al., *Positron emission tomography and fluorodeoxyglucose studies of metabolic hyperfrontality and psychopathology in the psilocybin model of psychosis.* Neuropsychopharmacology, 1997. **16**(5): p. 357-72.

73. Gouzoulis-Mayfrank, E., et al., *Neurometabolic effects of psilocybin, 3,4-methylenedioxyethylamphetamine (MDE) and d-methamphetamine in healthy volunteers. A double-blind, placebo-controlled PET study with [18F]FDG.* Neuropsychopharmacology, 1999. **20**(6): p. 565-81.

74. Riba, J., et al., *Increased frontal and paralimbic activation following ayahuasca, the pan-Amazonian inebriant.* Psychopharmacology (Berl), 2006. **186**(1): p. 93-8.

75. Carhart-Harris, R.L., et al., *Neural correlates of the psychedelic state as determined by fMRI studies with psilocybin.* Proc Natl Acad Sci U S A, 2012. **109**(6): p. 2138-43.

76. Carhart-Harris, R.L., et al., *Functional Connectivity Measures After Psilocybin Inform a Novel Hypothesis of Early Psychosis.* Schizophr Bull, 2012.

77. Carhart-Harris, R.L., et al., *Implications for psychedelic-assisted psychotherapy: functional magnetic resonance imaging study with psilocybin.* Br J Psychiatry, 2012. **200**(3): p. 238-44.

78. Dawson, D.A., R.B. Goldstein, and B.F. Grant, *Rates and correlates of relapse among individuals in remission from DSM-IV alcohol dependence: a 3-year follow-up.* Alcohol Clin Exp Res, 2007. **31**(12): p. 2036-45.

79. James, W., *The varieties of religious experience.* 1902, Cambridge, MA: Harvard University Press.

80. Miller, W.R., *The phenomenon of quantum change.* J Clin Psychol, 2004. **60**(5): p. 453-60.

81. Miller, W.R. and J. C'de_Baca, *Quantum change : when epiphanies and sudden insights transform ordinary lives*. 2001, New York: Guilford Press.

82. Forcehimes, A.A., *De profundis: spiritual transformations in Alcoholics Anonymous.* J Clin Psychol, 2004. **60**(5): p. 503-17.

83. Kaskutas, L.A., et al., *The role of religion, spirituality and Alcoholics Anonymous in sustained sobriety.* Alcoholism Treatment Quarterly, 2003. **21**: p. 1-16.

84. Robinson, E.A., K.J. Brower, and E. Kurtz, *Life-Changing Experiences, Spirituality and Religiousness of Persons Entering Treatment for Alcohol Problems.* Alcoholism Treatment Quarterly, 2003. **21**(4): p. 3-16.

85. Grof, S., *LSD psychotherapy*. 4th ed. 2008, Ben Lomond, CA: Multidisciplinary Association for Psychedlic Studies.

86. Leuner, H., *Present state of psycholytic therapy and its possibilities*, in *The use of LSD in psychotherapy and alcoholism*, H.A. Abramson, Editor. 1967, Bobbs-Merrill: Indianapolis. p. 101-116.

87. Buckman, J., *Theroetical aspects of LSD therapy*, in *The use of LSD in psychotherapy and alcoholism*, H.A. Abramson, Editor. 1967, Bobbs-Merrill: Indianapolis. p. 83-100.

88. Hoffer, A., *A program for treatment of alcoholism: LSD, malvaria, and nicotinic acid.*, in *The use of LSD in psychotherapy and alcoholism*, H.A. Abramson, Editor. 1967, Bobbs-Merrill: Indianapolis. p. 343-406.

89. Sherwood, J.N., M.J. Stolaroff, and W.W. Harman, *The psychedelic experience--a new concept in psychotherapy.* J Neuropsychiatr, 1962. **4**: p. 69-80.

90. Unger, S.M., *Mescaline, Lsd, Psilocybin, and Personality-Change - a Review.* Psychiatry, 1963. **26**(2): p. 111-125.

91. McGlothlin, W., S. Cohen, and M.S. McGlothlin, *Long Lasting Effects of Lsd on Normals.* Archives of General Psychiatry, 1967. **17**(5): p. 521-&.

92. Mogar, R.E. and C. Savage, *Personality-Change Associated with Psychedelic (Lsd) Therapy - a Preliminary-Report.* Psychotherapy-Theory Research and Practice, 1964. **1**(4): p. 154-162.

93. Savage, C., et al., *The effects of psychedelic (LSD) therapy on values, personality, and behavior.* Int J Neuropsychiatry, 1966. **2**(3): p. 241-54.

94. Bottrill, J.H., *Personality change in LSD users.* J Gen Psychol, 1969. **80**(2d Half): p. 157-61.

95. Baggott, M.J., et al., *Investigating the Mechanisms of Hallucinogen-Induced Visions Using 3,4-Methylenedioxyamphetamine (MDA): A Randomized Controlled Trial in Humans.* PLoS ONE, 2010. **5**(12): p. 13.

96. Griffiths, R.R., et al., *Mystical-type experiences occasioned by psilocybin mediate the attribution of personal meaning and spiritual significance 14 months later.* J Psychopharmacol, 2008.

97. Griffiths, R.R., et al., *Psilocybin can occasion mystical-type experiences having substantial and sustained personal meaning and spiritual significance.* Psychopharmacology (Berl), 2006. **187**(3): p. 268-83; discussion 284-92.

98. Griffiths, R.R., et al., *Psilocybin occasioned mystical-type experiences: immediate and persisting dose-related effects.* Psychopharmacology (Berl), 2011.

99. Bogenschutz, M.P. and J.M. Pommy, *Therapeutic mechanisms of classic hallucinogens in the treatment of addictions: from indirect evidence to testable hypotheses.* Drug Test Anal, 2012. **4**(7-8): p. 543-55.

100. Sobell, L.C., et al., *Reliability of a timeline method: assessing normal drinkers' reports of recent drinking and a comparative evaluation across several populations.* British Journal of Addiction, 1988. **83**: p. 393-402.

101. Miller, W.R., J.S. Tonigan, and R. Longabaugh, *The Drinker Inventory of Consequences (DrInC): An instrument for assessing adverse consequences of alcohol abuse. Test Manual (Vol. 4)*. 1995, Rockville, MD: US Government Printing Office.

102. Miller, W.R. and S. Rollnick, *Motivational interviewing: Preparing people for change*. 2nd ed. 2002, New York: Guilford Press.

103. Miller, W.R., et al., *Motivational enhancement therapy manual: A clinical research guide for therapists treating individuals with alcohol abuse and dependence*. Project MATCH Monograph Series, ed. M.E. Mattson. Vol. 2. 1992, Rockville, MD: National Institute on Alcohol Abuse and Alcoholism.

104. Obert, J.L. and C. Farentinos, *A Manual for Motivational Enhancement Treatment (MET) to Improve Treatment Engagement and Outcome in Subjects Seeking Treatment for Substance Abuse*. 2000, Manual for Protocol 0004, NIDA Clinical Trials Network.

105. Johnson, M., W. Richards, and R. Griffiths, *Human hallucinogen research: guidelines for safety.* J Psychopharmacol, 2008. **22**(6): p. 603-20.

106. Grob, C.S., et al., *Pilot study of psilocybin treatment for anxiety in patients with advanced-stage cancer.* Arch Gen Psychiatry, 2011. **68**(1): p. 71-8.

107. Chwelos, N., et al., *Use of d-lysergic acid diethylamide in the treatment of alcoholism.* Q J Stud Alcohol, 1959. **20**: p. 577-90.

108. Strassman, R.J. and C.R. Qualls, *Dose-response study of N,N-dimethyltryptamine in humans. I. Neuroendocrine, autonomic, and cardiovascular effects.* Arch Gen Psychiatry, 1994. **51**(2): p. 85-97.

109. Johnson, M.W., R. Andrew Sewell, and R.R. Griffiths, *Psilocybin dose-dependently causes delayed, transient headaches in healthy volunteers.* Drug Alcohol Depend, 2012. **123**(1-3): p. 132-40.

110. Griffiths, R.R., et al., *Psilocybin occasioned mystical-type experiences: immediate and persisting dose-related effects.* Psychopharmacology (Berl), 2011. **218**(4): p. 649-65.

111. Folstein, M.F., S.E. Folstein, and P.R. McHugh, *"Mini-mental state". A practical method for grading the cognitive state of patients for the clinician.* J Psychiatr Res, 1975. **12**(3): p. 189-98.

112. Hasler, F., et al., *Acute psychological and physiological effects of psilocybin in healthy humans: a double-blind, placebo-controlled dose-effect study.* Psychopharmacology (Berl), 2004. **172**(2): p. 145-56.

113. Pahnke, W., *Drugs and mysticism: an analysis of the relationship between psychedelic drugs and the mystical consciousness*. 1963, Harvard University.

114. Preston, K.L., et al., *Subjective and behavioral effects of diphenhydramine, lorazepam and methocarbamol: evaluation of abuse liability.* J Pharmacol Exp Ther, 1992. **262**(2): p. 707-20.

115. Mumford, G.K., K. Sliverman, and R.R. Grioffiths, *Reinforcing, subjective, and performance effects of lorazepam and diphenhydramine in humans.* Experimental and Clinical Psychopharmacology, 1996. **4**(4): p. 421-430.

116. Feltner, D.E. and G. Haig, *Evaluation of the subjective and reinforcing effects of diphenhydramine, levetiracetam, and valproic acid.* J Psychopharmacol, 2011. **25**(6): p. 763-73.

117. Jaffe, J.H., et al., *A postmarketing study of relative abuse liability of hypnotic sedative drugs.* Addiction, 2004. **99**(2): p. 165-73.

118. Griffiths, R.R. and M.W. Johnson, *Relative abuse liability of hypnotic drugs: a conceptual framework and algorithm for differentiating among compounds.* J Clin Psychiatry, 2005. **66 Suppl 9**: p. 31-41.

119. Griffiths, R., et al., *Mystical-type experiences occasioned by psilocybin mediate the attribution of personal meaning and spiritual significance 14 months later.* J Psychopharmacol, 2008. **22**(6): p. 621-32.

120. Maclean, K.A., M.W. Johnson, and R.R. Griffiths, *Mystical experiences occasioned by the hallucinogen psilocybin lead to increases in the personality domain of openness.* J Psychopharmacol, 2011. **25**(11): p. 1453-61.

121. Miller, W.R., ed. *Combined Behavioral Intervention manual: A clinical research guide for therapists treating people with alcohol abuse and dependence (Vol. 1)*. 2004, National Institute on Alcohol Abuse and Alcoholism: Bethesda, MD.

122. First, M.B., et al., *Structured Clinical Interview for DSM-IV Axis I Disorders-Patient Edition*. 1997, New York, NY: Biometrics Research Department, New York State Psychiatric Institute.

123. Sullivan, J.T., et al., *Assessment of alcohol withdrawal: The revised Clinical Institute Withdrawal Assessment for Alcohol scale (CIWA-Ar).* British Journal of Addiction, 1989. **84**: p. 1353-1357.

124. Sobell, L.C. and M.B. Sobell, *Timeline follow-back: A technique for assessing self-reported alcohol consumption*, in *Measuring alcohol consumption: Psychosocial and biological methods*, R.A. Litten and J.P. Allen, Editors. 1992, Humana Press: Totowa, NJ.

125. Sobell, L.C. and M.B. Sobell, *Timeline Follow Back: A calendar method for assessing alcohol and druq use (User's Guide).* 1996, Toronto: Addiction Research Foundation.

126. Sobell, L.C., et al., *Reliabiity of the Alcohol Timeline Followback when administered by telephone ad by computer.* Drug and Alcohol Dependence, 1996. **42**: p. 49-54.

127. Carey, K.B., *Reliability and validity of the Time-Line Follow-Back Interview among psychiatric outpatients: A preliminary report.* Psychol Addict Behav, 1997. **11**: p. 26-33.

128. Boscolo-Berto, R., et al., *Ethyl glucuronide concentration in hair for detecting heavy drinking and/or abstinence: a meta-analysis.* Int J Legal Med, 2013. **127**(3): p. 611-9.

129. Morini, L., L. Politi, and A. Polettini, *Ethyl glucuronide in hair. A sensitive and specific marker of chronic heavy drinking.* Addiction, 2009. **104**(6): p. 915-20.

130. Dittrich, A., *The standardized psychometric assessment of altered states of consciousness (ASCs) in humans.* Pharmacopsychiatry, 1998. **31**: p. 80-84.

131. Riba, J., et al., *Psychometric assessment of the Hallucinogen Rating Scale.* Drug Alcohol Depend, 2001. **62**(3): p. 215-23.

132. Hood, R.W.J., et al., *Dimensions of the mysticism scale: confirming the three-factor structure in the United States and Iran.* J Sci Study Relig, 2001. **40**: p. 691-705.

133. Pahnke, W.N., *Psychedelic drugs and mystical experience.* Int Psychiatry Clin, 1969. **5**: p. 149-162.

134. Richards, W.A., et al., *The peak experience variable in DPT-assisted psychotherapy with cancer patients.* J Psychedelic Drugs, 1977. **9**: p. 1-10.

135. Turek, I.S., R.A. Soskin, and A.A. Kurland, *Methylenedioxyamphetamine (MDA) subjective effects.* J Psychedelic Drugs, 1974. **6**: p. 7-14.

136. Piedmont, R.L., *Does spirituality represent the sixth factor of personality? Spiritual transcendence and the five-factor model.* J Person, 1999. **67**: p. 985-1013.

137. Fetzer Institute, *Multidimensional measurement of religiousness/spirituality for use in health research*. 1999, Kalamazoo, MI: Fetzer Institute.

138. McCrae, R.R., P.T. Costa Jr, and T.A. Martin, *The NEO-PI-3: a more readable revised NEO Personality Inventory.* J Pers Assess, 2005. **84**(3): p. 261-70.

139. Costa Jr., P.T. and R.R. McCrae, *Revised NEO Personality Inventory (NEO-PI-R) and NEO Five Factor Inventory (NEOFFI) Professional Manual*. 1992, Odessa, FL: Psychological Assessment Resources.

140. Schwartz, S.H., *Universals in the content and structure of values: Theory and empirical tests in 20 countries*, in *Advances in experimental social psychology*, M. Zanna, Editor. 1992, Academic Press: New York. p. 1-65.

141. Schwartz, S.H., *Les valeurs de base de la personne: théorie, mesures et applications [Basic human values: theory, measurement, and applications].* Revue française de sociologie, 2006. **42**(1): p. 249-288.

142. Forcehimes, A., Miller, W. R., Steele, J. M., & Tonigan, J. S. , *A simple measure of motivation: Importance, confidence and readiness for change in drinking.* Alcoholism: Clinical and Experimental Research, 2005. **29**(5, Supplement): p. 155 (Abstract).

143. DiClemente, C.C., et al., *The Alcohol Abstinence Self-Efficacy scale.* J Stud Alcohol, 1994. **55**(2): p. 141-8.

144. Project MATCH Research Group, *Project MATCH secondary a priori hypotheses.* Addiction, 1997. **92**(12): p. 1671-98.

145. Vielva, I. and I. Iraurgi, *Cognitive and behavioural factors as predictors of abstinence following treatment for alcohol dependence.* Addiction, 2001. **96**(2): p. 297-303.

146. Morgenstern, J., et al., *Affiliation with Alcoholics Anonymous after treatment: a study of its therapeutic effects and mechanisms of action.* J Consult Clin Psychol, 1997. **65**(5): p. 768-77.

147. Connors, G.J., J.S. Tonigan, and W.R. Miller, *A longitudinal model of intake symptomatology, AA participation and outcome: retrospective study of the project MATCH outpatient and aftercare samples.* J Stud Alcohol, 2001. **62**(6): p. 817-25.

148. Flannery, B.A., J.R. Volpicelli, and H.M. Pettinati, *Psychometric properties of the Penn Alcohol Craving Scale.* Alcohol Clin Exp Res, 1999. **23**(8): p. 1289-95.

149. Neff, K.D., *The development and validation of a scale to measure self-compassion.* Self and Identity, 2003. **2**: p. 223-250.

150. Raes, F., et al., *Construction and factorial validation of a short form of the Self-Compassion Scale.* Clin Psychol Psychother, 2011. **18**(3): p. 250-5.

151. Hamilton, M., *The assessment of anxiety status by rating.* British Journal of Medical Psychology, 1959. **32**: p. 50-55.

152. Hamilton, M., *A rating scale for depression.* J Neurol Neurosurg Psychiatry, 1960. **23**: p. 56-62.

153. Carleton, R.N., M.P. Abrams, and G.J. Asmundson, *The Attentional Resource Allocation Scale (ARAS): psychometric properties of a composite measure for dissociation and absorption.* Depress Anxiety, 2010. **27**(8): p. 775-86.

154. Tellegen, A. and G. Atkinson, *Openness to absorbing and self-altering experiences ("absorption"), a trait related to hypnotic susceptibility.* J Abnorm Psychol, 1974. **83**(3): p. 268-77.

155. Carlson, E.B. and F.W. Putnam, *An update on the dissociative experiences scale.* Dissociation, 1993. **6**: p. 16-27.

156. Bujarski, S., et al., *The effects of drinking goal on treatment outcome for alcoholism.* J Consult Clin Psychol, 2013. **81**(1): p. 13-22.

157. Strassman, R.J., et al., *Dose-response study of N,N-dimethyltryptamine in humans. II. Subjective effects and preliminary results of a new rating scale.* Arch Gen Psychiatry, 1994. **51**(2): p. 98-108.

158. Comer, S.D., M.A. Sullivan, and G.K. Hulse, *Sustained-release naltrexone: novel treatment for opioid dependence.* Expert Opin Investig Drugs, 2007. **16**(8): p. 1285-94.

159. Srisurapanont, M. and N. Jarusuraisin, *Naltrexone for the treatment of alcoholism: a meta-analysis of randomized controlled trials.* Int J Neuropsychopharmacol, 2005. **8**(2): p. 267-80.

160. Johnson, B.A., et al., *Oral topiramate for treatment of alcohol dependence: a randomised controlled trial.* Lancet, 2003. **361**(9370): p. 1677-85.

161. Johnson, B.A., et al., *Topiramate for treating alcohol dependence: a randomized controlled trial.* JAMA, 2007. **298**(14): p. 1641-51.

162. Simpson, T.L., et al., *A pilot trial of the alpha-1 adrenergic antagonist, prazosin, for alcohol dependence.* Alcohol Clin Exp Res, 2009. **33**(2): p. 255-63.

163. Gable, R.S., *Comparison of acute lethal toxicity of commonly abused psychoactive substances.* Addiction, 2004. **99**(6): p. 686-96.

164. Abraham, H.D., A.M. Aldridge, and P. Gogia, *The psychopharmacology of hallucinogens.* Neuropsychopharmacology, 1996. **14**(4): p. 285-98.

165. Brown, R.T., et al., *Pharmacokinetics of Escalating Doses of Oral Psilocybin in Healthy Adults.* Clin Pharmacokinet, 2017. **56**(12): p. 1543-1554.

166. Wolf, B., et al., *Abuse liability of diphenhydramine in sedative abusers.* NIDA Res Monogr, 1989. **95**: p. 486-7.

167. UDL Laboratories, I., *Dihpenhydramine Hydrochloride (package insert)*. 2007, UDL Laboratories, Inc.: Rockford, IL.

Statistical analysis plan for a double-blind trial of psilocybin-assisted treatment of alcohol dependence

ClinicalTrials.gov Identifier: NCT02061293

NYU SoM IRB Protocol#: 14-00614

IND Protocol#: 113080-02

Table of Contents

ADMINISTRATIVE INFORMATION 3
1. TITLE AND TRIAL REGISTRATION 3
2. VERSION NUMBER, PROTOCOL NUMBER, CHANGE SUMMARY 3
3. ROLES AND RESPONSIBILITIES 4

INTRODUCTION 5
4. BACKGROUND AND RATIONALE 5
5. OBJECTIVES 5

STUDY METHODS 6
6. TRIAL DESIGN 6
7. RANDOMIZATION 6
8. SAMPLE SIZE 6
9. HARMS 6
10. FRAMEWORK OF PRIMARY HYPOTHESES 7
11. STATISTICAL INTERIM ANALYSIS AND STOPPING GUIDANCE 7
12. TIMING OF FINAL ANALYSIS 8
13. TIMING OF OUTCOME ASSESSMENTS 9

STATISTICAL PRINCIPLES 10
14. CONFIDENCE INTERVALS AND P VALUES 10
15. ADHERENCE AND PROTOCOL DEVIATIONS 10
16. ANALYSIS POPULATIONS 11

TRIAL POPULATION 11
17. ELIGIBILITY 11
18. RECRUITMENT 13
19. WITHDRAWAL/FOLLOW-UP 14
20. SCREENING DATA 14
21. BASELINE PATIENT CHARACTERISTICS 15
22. OUTCOME DEFINITIONS 16
23. ANALYSIS METHODS 18
24. MISSING DATA 19
25. ADDITIONAL ANALYSES 19
26. STATISTICAL SOFTWARE REFERENCES 22

REFERENCES 22

LIST OF ABBREVIATIONS AND DEFINITION OF TERMS 23
27. AMENDMENT 1: INTRODUCTION OF MANOVA TO RESOLVE INDETERMINACY FOLLOWING EXECUTION OF PRIMARY ANALYSIS PLAN 24

Administrative Information

1. Title and Trial Registration

Title: Statistical analysis plan for a double-blind trial of psilocybin-assisted treatment of alcohol dependence
Trial Registration: ClinicalTrials.gov Identifier: NCT02061293
NYU SoM IRB Protocol#: 14-00614; IND Protocol#: 113080-02

2. Version Number, Protocol Number, Change Summary

Date	Protocol version no.	SAP version no.	Summary of changes
May 11, 2020	4.11	1.0	SAP created
March 31, 2021	4.11	1.1	Detail specifying removal of site effect along with the site-by-treatment interaction on page 17 corrected to reflect language in earlier versions of protocol (<version 4.10).<br><br>Amendment regarding primary analysis added (Amendment 1).
November 29, 2021	4.11	1.2	Updated means/statistics in Amendment 1 with corrected drinking data following QA on primary outcome.<br><br>Minor edit: Hedges' d → Hedges' g
January 7, 2022	4.11	1.3	Updated Means/statistics in Amendment 1 with corrected drinking data following discovery and correction of data processing error.<br><br>Replaced "DPDD" (drinks per drinking day) with "DPD" (drinks per day) in Aim 2 of analysis plan and removed DPDD from the Figure in Amendment 1, as DPDD was not intended to be a key secondary outcome.<br><br>Edited text and order of material in Amendment 1 for clarity.

Date	Protocol version no.	SAP version no.	Summary of changes
March 20, 2022	4.11	1.4	Updated Means/statistics in Amendment 1 with corrected drinking data following discovery and correction of data entry error (TLFB data for participant 2127 week 4 was added to the data set and ANOVAs were re-run).<br>Corrected an error in section 16, page 11: correct number of randomized participants was 95, not 96.

3. Roles and Responsibilities

Names, affiliations, and roles of SAP contributors:

J. Scott Tonigan, PhD; Professor, Department of Psychology, University of New Mexico: *SAP author*

Sarah E. Mennenga, PhD; Postdoctoral Researcher, Department of Psychiatry, New York University School of Medicine: *Primary statistician*

Eugene M. Laska, PhD; Professor, Department of Psychiatry, New York University School of Medicine: *Senior/supervising statistician*

Michael P. Bogenschutz, MD; Professor, Department of Psychiatry, New York University School of Medicine: *Principal investigator/clinical lead*

Introduction

4. Background and Rationale

Of all drugs of abuse, alcohol is possibly the most damaging in the US and globally when factoring in preventable death, premature death, disability, healthcare/societal costs, adverse medical and neuropsychiatric complications, unintentional injuries, and its significant causal link to suicidal and violent behaviors. Alcohol use disorders (AUDs) are highly prevalent in the US, are among the most disabling of all diseases worldwide, and are associated with a staggering economic and societal cost [5][6][7]. The effects of currently available treatments for addiction remain disappointingly small, particularly for pharmacotherapies other than agonist medications such as methadone [8].

In the 1950s through early 1970s there was extensive research on the use of the prototypical classic hallucinogen LSD in the treatment of alcoholism (For reviews see [11-15]). A recent meta-analysis [1] examined 6 randomized trials (4 of which were fully double-blind) of LSD for alcohol dependence [21-26]. A total of 325 participants received active treatment with LSD, and 211 received control treatment. At the first post-treatment follow-up (ranging from 1 month to 12 months) the odds ratio for improvement was 1.96, favoring LSD (95% confidence interval 1.36-2.84, $Z = 3.59$, $p = .0003$). These robust effects provide a strong rationale for resuming clinical investigation of classic hallucinogens for the treatment of alcoholism and other addictions.

5. Objectives

Aim 1: To characterize the acute effects of PO psilocybin 25 mg/70 kg, 30 mg/70 kg, and 40 mg/70 kg in alcohol dependent patients.

Hypothesis 1: The drug will be well-tolerated, and acute effects will be similar to those observed in normal volunteers.

Aim 2: To evaluate the effect of psilocybin treatment on drinking outcomes for 32 weeks after the first administration, relative to diphenhydramine control.

Hypothesis 2: A greater decrease in percent heavy drinking days will be observed among participants that receive psychoactive doses of psilocybin in combination with psychosocial treatment than in those that receive diphenhydramine.

Aim 3: To test whether or not characteristics of the drug administration session experiences mediate effects of psilocybin on short term (1 week) persisting effects and post-session drinking behavior.

Hypothesis 3: Intensity of experience during the drug administration sessions will predict post-session changes in craving, self-efficacy, and motivation, and these changes will in turn predict changes in drinking behavior during the 32 weeks following initial drug administration.

Aim 4: To evaluate the explanatory value of changes in alcohol craving, self-efficacy, motivation, and other psychological domains in accounting for the observed experimental effect of psilocybin relative to diphenhydramine control.

Hypothesis 4: Changes in craving, self-efficacy, and motivation will mediate the effects of psilocybin treatment on drinking behavior.

Aim 5: To evaluate pre-post changes in drinking in participants after they receive psilocybin in the third session.

Hypothesis 5: participants in the control group will show decreases in drinking during the 16 weeks following the psilocybin session at week 38.

Study Methods

6. Trial Design

The trial is a two-site, randomized, double-blind, active-controlled trial (n = 180, randomized 1:1) contrasting the acute and persisting effects of psilocybin to those of diphenhydramine in the context of outpatient alcoholism treatment.

7. Randomization

Randomization is performed as close as possible to the first drug administration session in order to restrict the intent-to-treat sample to patients who receive study medication. The randomization is stratified by site and consists of balanced varied size blocks within strata to ensure relative equality of assignment across treatment groups. The block sizes are not revealed to participating investigators and are randomly selected from a small number of different block sizes to help reduce the likelihood of an investigator predicting the next treatment assignment. The Principal Investigator (MPB) specified the randomization structure and the study pharmacist generated the randomization list and assigns treatment sequentially according the sequence for each site.

8. Sample Size

Power analysis is based on the primary outcome analysis contrasting the psilocybin group with the control group with respect to percent heavy drinking days in months 1-8 post pharmacologic treatment (Aim 2). With a total sample size of n = 180 (90 assigned to psilocybin and 90 to control), and 5% attrition, our power analysis assumed that four predictors would be in the regression-based HLM model.

We estimated at the outset of the trial that the study has power of 0.8 to detect an effect of size $f^2 = .065$, with alpha = 0.05 (2-tailed) and no correction for multiple comparisons. This effect is comparable to that of naltrexone [9, 158, 159], and smaller than those observed in some recent trials of other promising medications [160-162]. Regardless of the statistical significance of these changes, standardized and non-standardized effect sizes will be computed for primary and secondary outcome variables. The addition of an open-label optional third dosing does not affect the required sample size for Aim 2 (primary outcome), but approximately doubles the sample size for Aim 1 and allows for the addition of Aim 5.

With our final sample size of 93 (modified ITT sample) and primary outcome measured at 8 timepoints, we are powered at 0.8 to detect a small effect of f = 0.10 (d = 0.2) within the MMRM framework at $\alpha = 0.05$.

9. Harms

All AEs (including new AEs, worsening of baseline conditions, clinically significant laboratory findings, disease-related signs and symptoms that were not present at baseline and any event or finding that the investigator feels is clinically significant), regardless of causality or severity will be recorded and all treatment-emergent AEs will be analyzed.

Treatment Emergent Adverse Event (TEAE): An AE that begins after the first administration of the study drug.

Temporally-Related AEs (TRAEs): TEAEs that have an onset concurrent with, or within 24 hours following, administration of study medication in the two double-blind medication sessions.

All possibly or probably related AEs, severe AEs, and all SAEs will be recorded, along with the following information:

- Name of the event (coded)
- Duration (days)
- Resolution (y/n)
- Severity:
  - *Mild:* awareness of sign or symptom, but easily tolerated; usually does not require intervention
  - *Moderate:* discomfort sufficient to cause interference with normal activities; intervention may be needed
  - *Severe:* incapacitating, with inability to perform normal activities; treatment or other intervention usually needed
- Relationship to study drug:
  - *Probable:* This category applies to AEs that are considered, with a high degree of certainty, to be related to the study drug. An AE may be considered probable, if (must have first two):
    - It follows a reasonable temporal sequence from administration of the drug.
    - It cannot be reasonably explained by the known characteristics of the subject's clinical state, environmental or toxic factors, or other modes of therapy administered to the subject.
    - It follows a known pattern of response to the suspected drug.
  - *Possible:* This category applies to those AEs in which the connection with the study drug administration appears unlikely but cannot be ruled out with certainty. An AE may be considered possible if, or when (must have first two):
    - It follows a reasonable temporal sequence from administration of the drug.
    - It may have been produced by the subject's clinical state, environmental or toxic factors, or other modes of therapy administered to the subject.
    - It follows a known pattern of response to the suspected drug.
- Action taken regarding study drug (none, dose adjusted, drug discontinued)
- Treatment (y/n)
- SAE (y/n)

10. Framework of primary hypotheses

Hypothesis 1 (safety): Non-inferiority
Hypothesis 2 (efficacy): Superiority

11. Statistical Interim Analysis and Stopping Guidance

The study is implemented in in three stages, with interim analyses after the first and second stages.

Stage 1: Following final protocol approval by all necessary authorities and procurement of necessary funding, staff will be trained, at least 4 pilot/training participants per site will be treated, and 20 participants will be randomized into the main trial across two sites (UNM and NYU, 10 per site). Pilot/training participants will be randomly assigned to one of the two treatments in blocks of 4, independent of the main trial. Therapists and participants will be blinded to treatment assignment. Pilot/training participants will be included in the main trial sample if 1: Therapy fidelity ratings are adequate, and 2: no protocol modifications are made that would invalidate their inclusion. At the end of this stage, feasibility and tolerability will be assessed, and necessary protocol modifications may be made.

Stage 2: An additional 80 participants will be randomized, with the possible addition of one or two additional sites to enhance recruitment capacity. At the end of this stage, sample size re-estimation will be performed by an unblinded statistician, and final sample size will be determined.

Stage 3: Assuming 180 participants are required, the 80 remaining participants will be randomized.

In addition, safety interim looks will be performed (without formal statistical testing) at the regular DSMB meetings or unscheduled times per the DSMB's request. Although at this time we are not planning a formal statistical interim analysis for efficacy or futility, such an interim analysis can be performed if requested by the DSMB or the sponsor.

Details of guidelines for stopping the trial early

Following completion of the interim analysis specified at the end of Stage 2, enrollment for the trial may be continued if funding allows for continued recruitment through 180 participants, and sample size re-estimation suggests that statistical power is insufficient after recruiting 100 participants into the main trial.

Decision to end recruitment on March 30, 2020

Following an indefinite mandatory suspension of research recruitment beginning on March 19, 2020 due to the outbreak of a novel coronavirus (COVID-19), it was determined that enrollment for this trial would be halted prior to completion of Stage 2. The final numbers of participants randomized at each site within the pilot and main trial are as follows:

University of New Mexico:

- 4 pilot participants
- 9 main trial participants

New York University School of Medicine:

- 4 pilot participants
- 79 main trial participants

12. Timing of Final Analysis

Stage 1: Baseline characteristics

Analysis of baseline sample characteristics are to be initiated by an unblinded statistician as soon as data lock is achieved for the required instruments through the week 4 assessment.

Stage 2: Acute Effects of Study Medication

Preliminary analyses of acute effects of treatment with psilocybin versus diphenhydramine are to be initiated by an unblinded statistician as soon as data lock is achieved for the required instruments through the week 8 assessment.

Stage 3: Double-Blind Outcomes
Analyses corresponding with aims 1-4 (including the primary outcome analysis) will be conducted by an unblinded statistician when data lock is achieved for the required instruments through the week 36 assessment.

Stage 4: Open-label Outcomes
Final planned analyses corresponding with Aims 1 and 5 will be conducted when data lock is achieved for the required instruments through the week 54 assessment.

13. Timing of Outcome Assessments

		Scr.	BL	W04	W05	W08	W09	W12	W24	W36	W38	W39	W42	W54
**Psychiatric and SUD assessment**	Structured Clinical Interview for DSM-IV (SCID), Family History Questionnaire	x												
**Other baseline characteristics**	Treatment Goal Form, Demographics Form, Attentional Resource Allocation Scale, Mysticism Scale A		x											
**Substance use and consequences**	Time-line Follow-back	x	x	x		x		x	x	x	x		x	x
	Short Inventory of Problems (past 3 months)		x					x	x	x				x
**Acute Hallucinogen Effects**	5D-ASC, HRS, SCQ, Monitor Rating Scale, Mysticism scale B, guesses of medication received			x		x					x			

**Motivation**	Readiness rulers		x	x	x	x	x	x	x	x	x	x	x	x
**Self-efficacy**	Alcohol Abstinence Self-efficacy Scale		x	x	x	x	x	x	x	x	x	x	x	x
**Craving**	Penn Alcohol Craving Scale		x	x	x	x	x	x	x	x	x	x	x	x
**Mood/Anxiety**	Ham-D, Ham-A		x	x	x	x	x	x	x	x	x	x	x	x
	Adverse Events			x	x	x	x	x	x	x	x	x	x	x
**Safety**	Vital Signs	x	x	x	x	x	x	x	x	x	x	x	x	x
	Visual Analog Scales for abuse potential							x	x	x			x	x
**Tx satisfact.**	Visual Analog Scales							x					x	

Visit windows:

There were no visit windows specified for the protocol, therefore there is substantial variability in actual inter-assessment-intervals. Since the primary outcome measure (TLFB) produces a continuous calendar of substance use throughout the trial, regardless of assessment date, no correction will be made to data from the TLFB for assessments that happen outside of the target date. Scores on all other assessments that fall outside of the following windows will be set to missing:

Assessment	Max days early (-)	Max days late (+)
wk 4, 8, 38	4	4
wk 5, 9, 39	3	14
wk 12, 24, 36, 42, 54	7	28

Statistical Principles

14. Confidence intervals and P values

Level of statistical significance (alpha level) will be set at p=0.05 throughout unless otherwise specified. Ninety-five percent confidence intervals will be provided for all statistics.

15. Adherence and protocol deviations

Adherence to the intervention (per protocol sample) will be defined as completing all of the following treatment visits: Preparation Sessions 1 & 2, Medication Session 1, Debrief 1, and META 1-7. Rates of adherence to the following levels of treatment will be reported within each arm and chi-square analysis will be used to determine whether rates of adherence differ between treatment groups within the modified ITT sample:

- **Per protocol (Completed Med 1 plus therapy):** Preparation Sessions 1 & 2, Medication Session 1, Debrief 1, and META 1-7

- **Completed Med 1 and 2 plus therapy:** Preparation Sessions 1-3, Medication Sessions 1-2, Debrief 1-2, and META 1-7
- **Completed Med 1 and 3 plus therapy:** Completed Preparation Sessions 1, 2 & 4, Medication Sessions 1 & 3, Debrief 1 & 3, and META 1-9
- **Completed Med 1-3 plus therapy:** Preparation Sessions 1-4, Medication Sessions 1-3, Debrief 1-3, and META 1-9

16. Analysis populations

Final analysis samples:

- *Intention to Treat (ITT):* Randomized sample (n=95)
- *Modified ITT:* Received study medication (n=93) *[Primary analysis sample]*
- *Per Protocol:* Completed Preparation Sessions 1 & 2, Medication Session 1, Debrief 1, and META 1-7
- *Complete Case:* Provided timeline followback and safety outcomes for 36 weeks after receiving medication

Trial Population

17. Eligibility

Participants will be recruited from the community through advertising and referral, according to recruitment plans to be developed locally at each site.

Participants will be:

1) Males and females age 25-65 with SCID (DSM-IV) diagnosis of alcohol dependence who
2) Want to stop or decrease their drinking
3) Are not participating in any formal treatment for alcohol dependence (12-step meetings are not considered treatment)
4) Are able to provide voluntary informed consent
5) Have at least 4 heavy drinking days in the past 30 days
6) If female of childbearing potential, are willing to use approved form of contraception[#] from screening until after the psilocybin administration sessions
7) Have a family member or friend who can pick them up and stay with them overnight after the psilocybin administration sessions
8) Are able to provide adequate locator information.

Participants will be excluded if they have:

1) Medical conditions that would preclude safe participation in the trial (e.g., seizure disorder, significantly impaired liver function*, coronary artery disease, history of arrhythmia, heart failure, uncontrolled hypertension (above 165/95 mmHg at screening), history of cerebrovascular accident, severe asthma^, hyperthyroidism, narrow-angle glaucoma, stenosing peptic ulcer, pyloroduodenal obstruction, symptomatic prostatic hypertrophy, or bladder-neck obstruction)
2) Exclusionary psychiatric conditions (schizophrenia, schizoaffective disorder, bipolar disorder, current major depressive episode, current post-traumatic stress disorder, current suicidality or history of medically serious suicide attempt)
3) Cognitive impairment (Folstein Mini Mental State Exam [111] score < 26)

4) A family history of schizophrenia or schizoaffective disorder (first or second degree relatives), or bipolar disorder type 1 (first degree relatives)
5) History of hallucinogen use disorder, any use in the past 1 year, or >25 lifetime uses;
6) Cocaine, psychostimulant, opioid, or cannabis dependence (past 12 months)
7) Current non-medical use of cocaine, psychostimulants, or opioids (past 30 days)
8) Significant alcohol withdrawal (CIWA-Ar score greater than 7. Patients presenting at screening in withdrawal may be referred for detoxification and reassessed within 30 days)
9) Serious ECG abnormalities (e.g., evidence of ischemia, myocardial infarction, QTc prolongation (QTc > 0.45 seconds for men, QTc > 0.47 seconds for women))
10) Serious abnormalities of complete blood count or chemistries
11) Active legal problems with the potential to result in incarceration
12) Pregnancy or lactation
13) Need to take medication with significant potential to interact with study medications (e.g., antidepressants, antipsychotics, psychostimulants, treatments for addictions, other dopaminergic or serotonergic agents, lithium, anticonvulsants).
14) Allergy or hypersensitivity to psilocybin or diphenhydramine.
15) High risk of adverse emotional or behavioral reaction based on investigator's clinical evaluation (e.g., evidence of serious personality disorder, antisocial behavior, serious current stressors, lack of meaningful social support).

Participants will receive study medication only if blood pressure is less than or equal to 140 systolic, 90 diastolic at safety screening on the day of the drug administration sessions.

18. Recruitment

CONSORT diagram to be completed:

CONSORT Flow Diagram: Double-Blind Phase [Weeks -4 – 36]

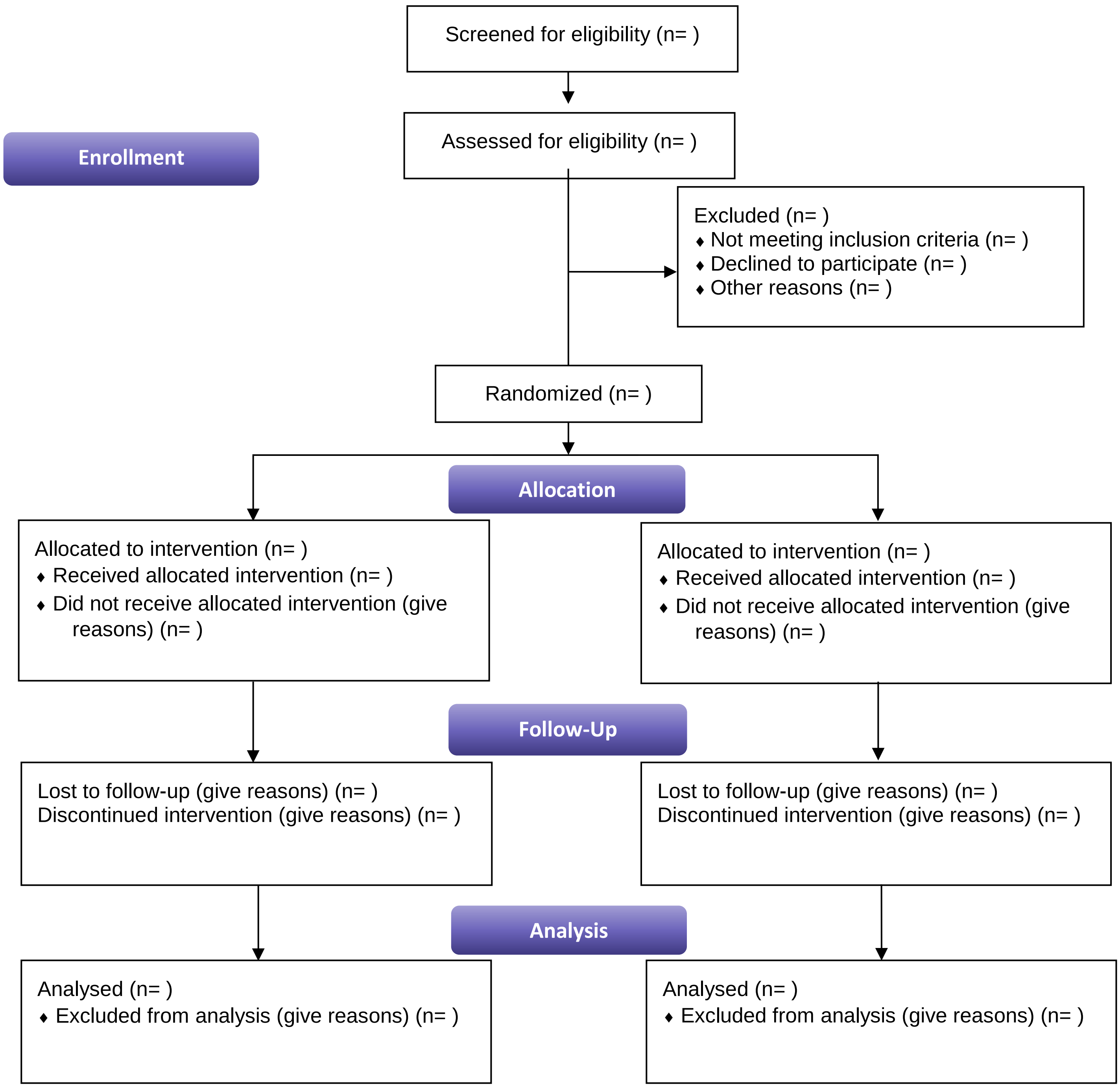

CONSORT Flow Diagram: Open-Label Extension Phase [Weeks 38 – 54]

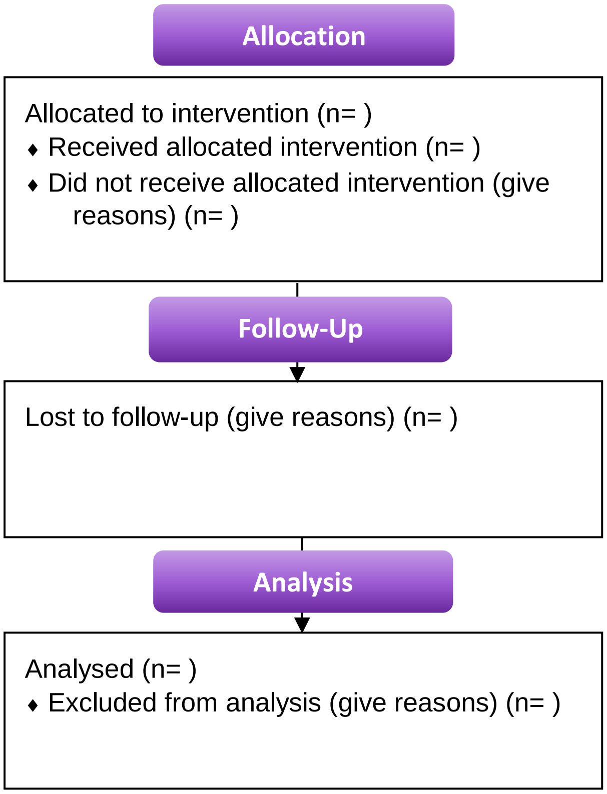

19. Withdrawal/follow-up

Participants can withdraw from participation at the following levels:

- *Medication withdrawal:* withdraw from further medication, but continue assessments and therapy
- *Treatment withdrawal:* withdraw from further medication and therapy, but continue assessments
- *Participation withdrawal:* withdraw from further medication, therapy, and assessments

Data from participants that received study medication, but withdraw from further medication (medication w/d) or medication and therapy (treatment w/d) will be included in the primary analyses. Partial data from participants that withdraw from assessments (participation w/d), or were LTFU prior to completion, will be included in the primary analyses. A count of per-protocol participants withdrawn from medication, treatment, and participation, and lost to follow-up will be presented by timing of withdrawal/LTFU relative to receipt of study medication. Frequencies of reasons for withdrawal following receipt of study medication will be generated.

20. Screening data

Descriptive information (means and standard deviations, or counts) will be generated for the following variables within participants that were screened by phone but not enrolled and within enrolled participants. Analysis of variance or chi square analysis will be used to

determine whether the enrolled sample differs from the pre-screened but not enrolled sample on the following variables [phone screen]:

- Age
- Gender
- Reasons for exclusion will be tabulated within participants that were pre-screened but not enrolled

Descriptive information (means and standard deviations, or counts) will be generated for the following variables in the enrolled but not randomized, and randomized sample. Analysis of variance or chi square analysis will be used to determine whether the randomized sample differs from the enrolled but not randomized sample on the following variables [phone screen/screening]:

- Number of lifetime hallucinogen uses
- Most recent hallucinogen use
- Age
- Gender
- Total family income
- Level of education
- Ethnic Group
- Living Situation
- Living situation safe and stable
- Marital status
- Employment status
- Degree
- Percent drinking days past 90 days
- Drinks per day past 90 days
- Percent heavy drinking days past 90 days

21. Baseline patient characteristics

Descriptive information (means and standard deviations/counts and other distributional properties) will be generated for the following variables measured prior to administration of study medication within each treatment group and for the whole ITT sample. Analysis of variance or chi square analysis will be used to determine treatment group equivalency, as well as differences between eligible prospects who do and do not drop out prior to receiving study medication, on primary substance use measures and other patient characteristics that are central to study aims, including the following variables:

- Number of AUD dependence criteria [SCID; screening]
- Age of AUD onset [SCID; screening]
- History of Mood Disorders [SCID; screening]
- History of Anxiety Disorders [SCID; screening]
- Demographics [screening]
- Family history questionnaire [screening]
- Treatment Goal Form [baseline]
- Mysticism Scale Form A [baseline]
- Drinking-related problems [Short Inventory of Problems; baseline]
- Readiness to reduce drinking [Readiness Rulers; baseline – week 4]
- Self-Efficacy [Alcohol Abstinence Self-Efficacy Scale; baseline – week 4]
- Alcohol Craving [Penn Alcohol Craving Scale; baseline – week 4]

- Mood [Hamilton Depression Scale; baseline – week 4]
- Anxiety [Hamilton Anxiety Rating Scale; baseline – week 4]
- Percent Heavy Drinking Days [Timeline Follow-back; 90 days prior to screening - wk4]
- Percent Days Abstinent [Timeline Follow-back; 90 days prior to screening - wk4]
- Drinks per Day [Timeline Follow-back; 90 days prior to screening - wk4]
- Drinks per Drinking Day [Timeline Follow-back; 90 days prior to screening - wk4]

22. Outcome Definitions

Primary Analysis: Double-Blind Outcomes

Assessments to be locked:

- Timeline Followback [screening – week 36]
- Short Inventory of Problems [weeks 12, 24, 36]
- Readiness Rulers [weeks 5, 8, 9, 12, 24, 36]
- Alcohol Abstinence Selfefficacy Scale [weeks 5, 8, 9, 12, 24, 36]
- Penn Alcohol Craving Scale [weeks 5, 8, 9, 12, 24, 36]
- Hamilton Depression Scale [weeks 5, 8, 9, 12, 24, 36]
- Hamilton Anxiety Rating Scale [weeks 5, 8, 9, 12, 24, 36]
- AEs [through week 36]
- Vital signs [week 4, 8]
- 5D-ASC [week 4, 8]
- MEQ [week 4, 8]
- Mysticism Scale Form B [week 4, 8]
- HRS [week 4, 8]
- Monitor ratings form [week 4, 8]
- Abuse Potential Visual Analog Scales [week 12]
- Treatment Satisfaction Visual Analog Scales [week 12]

Aim 1: To characterize the acute effects of PO psilocybin 25 mg/70kg, 30mg/70kg, and 40 mg/70kg in alcohol dependent patients.

The primary safety measures will be vitals signs (systolic and diastolic blood pressure (mmHg) and heart rate (BPM) from prior to medication administration to 30 min, 1 hr, 1hr30min, 2hrs, 3hrs, 4hrs, 5hrs, and 6hrs post medication administration at weeks 4, 8, and 38), abuse potential visual analog scales measured at wks 12, 24, 36, 42, and 54, number of Adverse Events (AEs), and number of Serious Adverse Events (SAEs) using the FDA definition.

The primary measure of hallucinogen effect is the 5-Dimensional Altered States of Consciousness Scale (5D-ASC; G-ASC subscale score) [130]; secondary outcomes are total and other subscale scores of the 5D-ASC, the Intensity and other subscale scores from the Hallucinogen Rating Scale (HRS) [157], the Interpretation, Introvertive, and Extrovertive subscale scores from the Mysticism scale [132], total and subscale scores from the Mystical Experience Questionnaire (MEQ) [97], and within-session Monitor Rating Scale Scores for Intensity, Anxiety, and Paranoia measured at weeks 4, 8, and 38.

Aim 2: To evaluate the effect of psilocybin treatment on drinking outcomes for 32 weeks after the first administration, relative to diphenhydramine control.

The primary outcome for hypothesis 2 is monthly percent heavy drinking days during weeks 5-36 (weeks 5-8, 9-12, 13-16, 17-20, 21-24, 25-28, 29-32, 33-36; the 32 weeks following the first drug administration session), assessed by the Timeline Follow-back [100]. Other

drinking variables from the same time frame will serve as secondary outcomes, (from the Short Inventory of Problems [101] at 12, 24, and 36 week assessments).

Covariates for Aim 2 analyses of drinking outcomes will include corresponding measures during weeks 1-4 (the 4 weeks prior to medication administration).

Aim 3: To test whether or not characteristics of the drug administration session experiences mediate effects of psilocybin on short term (1 week) persisting effects and post-session drinking behavior.

Mediators that will be examined include scores from the three main dimensions of the 5D-ASC (oceanic boundlessness, anxious ego-disintegration, visionary restructuralization) and the total score from the MEQ, measured at the week 4 assessment.

Dependent variables will be change in craving (total score from The Penn Alcohol Craving Scale [148]), self-efficacy (Abstinence Confidence from the Alcohol Abstinence Self-efficacy Scale [143]), and motivation (readiness score on the Readiness Rulers [142]), from the week 4 to week 5 assessment (pre- to post- medication), as well as drinking outcomes, expressed as change in percent heavy drinking days, percent days abstinent, and drinks per drinking day from pre- (4 – 0 weeks prior to medication session 1) to post- (1 to 4 weeks after medication session 1) medication administration.

Aim 4: To evaluate the explanatory value of changes in alcohol craving, self-efficacy, motivation, and other psychological domains in accounting for the observed experimental effect of psilocybin relative to diphenhydramine control.

Primary mediators will be change in craving (total score from The Penn Alcohol Craving Scale [148]), self-efficacy (Abstinence Confidence from the Alcohol Abstinence Self-efficacy Scale [143]), and motivation (readiness score from the Readiness Rulers [142]) from the week 4 to week 9 assessment (pre- to post- medication). Additional secondary mediators will include change in mood (Hamilton Depression Scale score [152]) and anxiety (Hamilton Anxiety Scale score) from the week 4 to week 9 assessment (pre- to post-medication).

Dependent variables will include drinking outcomes expressed as change in percent heavy drinking days, percent days abstinent, and drinks per drinking day from pre- (4 – 0 weeks prior to medication session 1) to post- (1 – 4 weeks following medication session 2) medication administration.

Open-Label Outcomes

Assessments to be locked:

- Timeline Followback [screening – week 54]
- Short Inventory of Problems [week 54]
- Readiness Rulers [weeks 38, 39, 42, 54]
- Alcohol Abstinence Selfefficacy Scale [weeks 38, 39, 42, 54]
- Penn Alcohol Craving Scale [weeks 38, 39, 42, 54]
- Hamilton Depression Scale [weeks 38, 39, 42, 54]
- Hamilton Anxiety Rating Scale [weeks 38, 39, 42, 54]
- AEs [through week 54]
- Vital signs [week 38]
- 5D-ASC [week 38]
- MEQ [week 38]
- Mysticism Scale Form B [week 38]
- HRS [week 38]

- Monitor ratings form [week 38]

Aim 5: <u>To evaluate pre-post changes in drinking in participants after they receive psilocybin in the third session.</u>

The primary drinking outcome for hypothesis 5 is monthly percent heavy drinking days during weeks 39-54 (weeks 39-42, 43-46, 47-50, 50-54) as assessed by the Time-line Follow-back [100]. Other drinking variables will serve as secondary outcomes, including percent days abstinent, drinks per drinking day, days to first drinking day, days to first heavy drinking day, and consequences of drinking (from the Short Inventory of Problems [101] at the week 54 assessment). Covariates for Aim 5 analyses of drinking outcomes will include corresponding measures during weeks 33-36 (the 4 weeks prior to week 36).

23. Analysis Methods

Preliminary analyses

For all analyses, in addition to the assessment of the distributional properties of key measures at follow-up, we will evaluate the nature and pattern of missing data.

Primary Analysis: Double-Blind Outcomes

Aim 1: <u>To characterize the acute effects of PO psilocybin 25 mg/70kg, 30mg/70kg, and 40 mg/70kg in alcohol dependent patients.</u>

Tolerability: Blood pressure, heart rate, and monitor ratings form item scores will be contrasted within the MMRM framework including fixed, categorical effects of treatment and assessment, a treatment-by-assessment interaction, and a fixed covariate (final score on each outcome prior to medication administration). Treatment-emergent AEs will be coded, tabulated, and treatment groups contrasted using Chi squared or Fisher's exact test.

<u>Psychoactivity</u>: Scores from the HRS and other scales (mean, SD, median, range) will be computed and reported. At week 4, scores will be contrasted by group (psilocybin 25 mg/70kg vs. diphenhydramine 50mg) using t-tests for independent samples. At week 8, scores will be contrasted by group (psilocybin 25 mg/70kg vs. psilocybin 30mg/70kg vs. psilocybin 40 mg/70kg vs. diphenhydramine 50mg vs. diphenhydramine 100mg) using analysis of variance. Mean scores will be compared descriptively to means reported in other studies, particularly Griffiths et al. [97, 110]. Visual analog scales for abuse potential will be contrasted between-groups within the MMRM framework including fixed, categorical effects of treatment and assessment, and a treatment-by-assessment interaction.

Aim 2: <u>To evaluate the effect of psilocybin treatment on drinking outcomes for 32 weeks after the first administration, relative to diphenhydramine control.</u>

To determine whether psilocybin treatment reduces drinking within participants and relative to diphenhydramine control, we will employ a MMRM including fixed, categorical effects of treatment, assessment, and site; site-by-treatment and treatment-by-assessment interactions; the continuous, a fixed covariate (week 1-4 PHDD); and using monthly values of PHDD (weeks 5-8, 9-12, 13-16, 17-20, 21-24, 25-28, 29-32, 32-36) as the dependent measure. The primary timepoint at which we expect the treatment groups to differ is during weeks 9-12. The site-by-treatment interaction will be removed from the final model if not significant at the $p < 0.05$ level. Secondary drinking measures with a continuous scale of measurement, e.g., PDA and DPD, will be tested in a similar fashion and for days to first drinking day and days to first heavy drinking day (5 or more standard drinks for a man, 4 or more standard drinks for a woman). Cox survival analysis will be used. Measures of effect size will be calculated for all

planned between-group contrasts (Hedges g) and the Q statistic will be calculated to assess the homogeneity of effect across the primary and secondary outcome measures of drinking.

Aim 3: To test whether or not characteristics of the drug administration session experiences mediate effects of psilocybin on short term (1 week) persisting effects and post-session drinking behavior.

Tests of statistical mediation will use a product-of-coefficients approach with a bootstrapping procedure. This choice provides the most powerful test of mediation with smaller samples and does not assume normality in the distribution of (a'b') product terms (Preacher and Hayes, 2008; Hayes, 2009). HLM will be applied to identify the a and b parameter estimates for the between-group contrast (treatment assignment psilocybin versus control; df = 1), and baseline values of the dependent variable will be entered in level 2. No protection for inflated Type I error will be applied to the testing of the three primary mediators. Performance site and the site by treatment group interaction terms will not be entered into the model.

Aim 4: To evaluate the explanatory value of changes in alcohol craving, self-efficacy, motivation, and other psychological domains in accounting for the observed experimental effect of psilocybin relative to diphenhydramine control.

Methods will be identical to those described for Aim 3.

Open-Label Outcomes

Aim 5: To evaluate pre-post changes in drinking in participants after they receive psilocybin in the third session.

Methods for Aim 5 will parallel those used in Aim 2, except that only participants in the control group will be included in this analysis, so treatment and site-by-treatment interactions will be omitted from the model, with the time effect being of principal interest. PHDD will be centered at week 42.

24. Missing Data

We will examine attrition by group assignment using hierarchical linear modeling (HLM) with binary outcomes depicting attrition status (yes/no) at each post-medication follow-up assessment. The main effect of group assignment on attrition as well as the potential for a group-by-time attrition effect will be examined.

If missing data can be assumed to be MAR or MCAR, the Full Information Maximum Likelihood (FIML) approach will be used to estimate the model using all available data. This approach employs multiple imputations and maximum likelihood estimation to estimate population parameters most likely to have given rise to the sample statistics.

25. Additional Analyses

To explore whether aspects of the drug experience are important for clinical outcomes, scores on acute hallucinogen measures will be evaluated as continuous independent variables predicting change in drinking within a regression framework. Measures of acute hallucinogen effects and drinking outcomes will be identical to those specified in Aim 3. If significant predictors of drinking outcomes are found, change in craving, self-efficacy, and motivation (as described in Aim 4) will be evaluated as mediators of the relationships between continuous scores on acute hallucinogen measures and change in drinking outcomes, again using only data from individuals that received the drug. Goals for treatment (abstinence vs. moderation) will be evaluated as a moderator of the treatment effect on drinking outcomes (IV and DV as described in Aim 3).

Acute Effects of Study Medication [Stage 2]

Description of medication adherence and analysis of acute effects of study medication will occur during stage 2 of analysis, and include data from the following instruments/timepoints. These analyses will assess the acute physiological, psychological, and experiential effects of study medication, as well as medication adherence, guesses of medication received, and safety during, and immediately after (within 24 hours of), the two double-blind drug administration sessions. Specific questions regarding baseline predictors of safety and acute hallucinogen effects will also be evaluated in this stage.

- 5D-ASC [week 8]
- MEQ [week 8]
- Mysticism Scale Form B [week 8]
- HRS [week 8]
- Monitor ratings form [week 8]
- Vital signs [week 8]
- AEs [TEAs Wk 4 + 1d Wk 8 + 1d]
- Guesses of medication received [week 4, 8]

We will report the number and proportion of: 1) ITT participants within each treatment group that participated in both medication sessions, and medication session 1 only; and 2) mITT participants within each treatment group that did not participate in med 2 due to participant preference, and due to a clinically significant AE. Chi square analysis will be used to analyze group equivalency in preference- and AE-prompted medication withdrawal.

Descriptive statistics (means and standard deviations) of participant and therapist guesses of medication received and confidence of guesses will be calculated and reported. Rates of disagreement between therapists, between therapists and participants, and within therapists and participants between medications sessions will be calculated and reported.

TRAEs (w/in 24 hours of medication administration) will be coded, tabulated, and treatment groups contrasted using Chi squared or Fisher's exact test.

The following baseline variables will be evaluated as predictors of physiological measures and clinician- and participant- rated psychological effects of the drug at the first medication session using regression analysis within psilocybin-treated participants. AUD-related variables and past psychedelic use variables will be entered into two separate predictive equations, and other variables will be evaluated separately.

- AUD-related:
  - Percent Heavy Drinking Days [Timeline Follow-back; 90 days prior to screening through medication administration]
  - Percent Days Abstinent [Timeline Follow-back; 90 days prior to screening - wk4]
  - Drinks per Day [Timeline Follow-back; 90 days prior to screening - wk4]
  - Drinks per Drinking Day [Timeline Follow-back; 90 days prior to screening - wk4]
  - Duration (years drinking)
  - Age of onset [SCID; screening]
  - #AUD symptoms [SCID; screening]
  - Number of family history of alcohol-related problems [Family history questionnaire screening]
- Number of past uses of psychedelics (by type)
  - Psilocybin/LSD [SCID; screening]
  - MDMA [SCID; screening]

  - Other [SCID; screening]
- Absorption [Attentional Resource Allocation scale; baseline]
- Lifetime mysticism [Mysticism scale A; baseline]

Outcomes measured repeatedly during drug administration sessions, including physiological measures (HR, systolic BP, diastolic BP) and clinician- rated psychological effects of the drug (Intensity, Anxiety, and Paranoia from the monitor ratings form), will be assessed using a between-groups nested repeated measures design, in which measures are nested within medication session.

Participant-rated effects of the drug (AHE scales) will be analyzed using repeated measures ANOVA, with scores 8 hours after each medication administration as the DV. IVs will include treatment arm (psilocybin vs. diphenhydramine) and dose of treatment assigned at the second medication session (25, 30, 40).

Additional exploratory analyses will begin to evaluate the structure of variance in scores on measures of various psychological dimensions of acute hallucinogen effects. Correlation matrices displaying intra-individual correlations between medication sessions on all orthogonal subscales of measures of acute hallucinogen effects, and within -individual and - medication session correlations between the various subscales will be generated within psilocybin-treated participants. Significance level will be set at 0.01 to partially adjust for number of comparisons, although these analyses are considered purely exploratory.

Whether weight-based dosing significantly enhances control of acute hallucinogen effects over flat dosing will be assessed using hierarchical regression analysis. For this analysis, scores on selected acute hallucinogen subscales (5D-ASC, G-ASC subscale score; HRS, Intensity subscale score) and change from baseline to peak measurements on cardiovascular variables (HR, systolic BP, diastolic BP) within psilocybin-treated participants at medication sessions 1 and 2 will first be predicted by a set of baseline variables, including mg/kg dose received (25, 30, or 40), administration sequence (1 or 2), and other significant baseline predictors of response in medication session 1 (from analyses specified above) (equation 1 below). Next, deviation of actual mg dose received from mg/kg dose amount (based on deviation of body weight from 70kg average) will be entered as an additional predictor to evaluate its predictive contribution, over and above the variables specified in the previous step (equation 2 below):

- Eq 1: DV = b1mg/kg + b2sequence + b3blvar1 ... +bXblvarX + b0
- Eq 2: DV = b1mg/kg + b2sequence + b3blvar1 ... +bXblvarX + b(X+1)mgdev + b0

Additional weight-based dosing analyses will be conducted as described above, using change in PHDD days (from [90d prior to bl] to [wk8-wk36]), days to first heavy drinking day (from wk8-wk36), and consequences of drinking (wk36) as dependent variables representing clinical change.

Proportion of mITT participants that meet the following binary treatment success criteria within each treatment group will be reported, and chi-square analysis will be used to contrast groups. Scores on the SIP will be contrasted using one-way ANOVA between participants that do and do not meet each of the following criteria:

- Abstinence (0 DD) during post-drug follow-up (from wk8-wk36)
- O HDD at post-drug 2 follow-up (from wk8-wk36)
- 60% reduction in PHDD from 90d pre-screen to post-drug follow-up (from wk8-wk36)

Descriptive statistics (means and standard deviations) will be reported within each treatment group on Treatment Satisfaction and Abuse Potential Visual Analog Scales measured at week 12. Treatment arms will be contrasted on mean treatment satisfaction and abuse potential ratings with one-way ANOVA.

Repeated measures ANOVA will be used to asses within-group change in PHDD, PDA, DPD, and DPDD across the first medication session [(bl-wk4) to (wk5-wk8)] versus across the second medication session [from (wk5-wk8) to (wk8-wk12)], and within-group change across the first medication session [(bl-wk4) to (wk5-wk8)], versus total change across both medication sessions [from (bl-wk4) to (wk8-wk12)].

26. Statistical Software References

Analyses will be conducted on IBM SPSS Statistics for Windows Version 25.0 (Released 2017, Armonk, NY: IBM Corp.)

References

Trial Master File and Statistical Master File are located at:

Bellevue Hospital Center, 462 First Ave, A Bldg, 8th Fl, Rm 836, New York, NY 10016

Standard Operating Procedures are maintained electronically at:

etc-research-01\EtOH-14-00614\4. Study Documentation\3. SOPs

Data Management Plan is maintained electronically at:

etc-research-01\EtOH-14-00614\9. Data and Statistical Analysis\SAP

List of Abbreviations and Definition of Terms

Abbreviation	Definition/Term
AE	adverse event
BP	blood pressure
CRF	case report form
DSMB	data and safety monitoring board
DSM-5	Diagnostic and Statistical Manual of Mental Disorders, Fifth Edition
FDA	Food and Drug Administration
HR	heart rate
IND	investigational new drug
IRB	Institutional review board
SAE	serious adverse event
TEAE	treatment-emergent adverse event
TRAE	temporally-related adverse event

27. Amendment 1: Introduction of MANOVA to resolve indeterminacy following execution of primary analysis plan

The original SAP for this trial included a parameter refinement step following the primary analysis that called for exclusion of the site-by-treatment interaction term, should it not be "significant at the p < .05 level." The intention of this refinement step was to remove non-meaningful terms from the final model, and there was no consideration made regarding which set of results (i.e. results from the model that included versus excluded the site-by-treatment interaction) would be interpreted as primary, should there be substantial disagreement between the two sets.

Upon executing the analyses as specified in the SAP, we discovered that the p-value associated with the treatment-by-site interaction was greater than 0.05 (p=0.104). Other estimates from this model indicate that the site-by-interaction term may be accounting for meaningful variance in the primary outcome (F=2.70; means and SEs/95%CIs for the interaction between treatment and site below).

rand_tx	rand_site	Mean	SE	95% CI Lower	95% CI Upper
0	1	27.69	7.14	13.50	41.88
	2	18.16	2.79	12.61	23.71
1	1	5.96	6.72	-7.39	19.29
	2	13.70	2.75	8.24	19.17

Moreover, removal of the site-by-treatment interaction substantially changed estimates of the treatment effect, an unintended consequence. While results overall offered support for a treatment effect on the primary outcome measure, the model including the site-by-treatment interaction term estimated the treatment effect to be considerably larger (F=6.20, p=0.015) than the model with the term removed (no other changes to the model) (F=3.47, p=0.066); group means and SEs/95% CIs from each model are below.

Model	rand_tx	Mean	SE	95% CI Lower	95% CI Upper
1	0	22.93	3.83	15.32	30.53
	1	9.83	3.63	2.62	17.04
2	0	19.57	3.28	13.05	26.09
	1	12.71	3.21	6.33	19.10

For the main secondary drinking outcomes (percent drinking days and drinks per day), each returned p-values greater than 0.05 for the site-by-treatment interaction, and removal of the site-by-treatment term substantially <u>increased</u> estimates of the treatment effect for these outcomes, as shown below.

		INCLUDING SITE X TREATMENT INTERACTION				*EXCLUDING SITE X TREATMENT INTERACTION*			
		Numerator df	Denominator df	F	p	Numerator df	Denominator df	F	p
PERCENT HEAVY DRINKING DAYS	Intercept	1	90.73	2.23	.139	1	90.225	1.956	.165
	Baseline	1	93.76	41.34	.000	1	93.158	40.478	.000
	Treatment	1	92.51	6.198	.015	1	90.260	3.465	.066
	Time	7	622.77	1.030	.408	7	622.256	1.038	.403
	Site	1	91.86	.028	.867	1	91.503	.005	.943
	Treatment x Time	7	622.75	.527	.815	7	622.240	.522	.818
	Treatment x Site	1	92.60	2.699	.104	-	-	-	-
PERCENT DRINKING DAYS	Intercept	1	92.56	1.086	.300	1	92.650	1.175	.281
	Baseline	1	93.42	74.329	.000	1	93.210	74.150	.000
	Treatment	1	94.49	4.218	.043	1	92.520	11.297	.001
	Time	7	624.66	2.254	.029	7	624.696	2.251	.029
	Site	1	94.49	.965	.328	1	94.744	1.009	.318
	Treatment x Time	7	624.65	.466	.860	7	624.692	.468	.858
	Treatment x Site	1	94.72	.154	.696	-	-	-	-
DRINKS PER DAY	Intercept	1	91.96	1.987	.162	1	91.832	1.854	.177
	Baseline	1	92.60	61.746	.000	1	92.352	61.776	.000
	Treatment	1	93.61	7.434	.008	1	91.618	9.424	.003
	Time	7	623.97	1.261	.267	7	623.819	1.266	.265
	Site	1	93.23	.024	.877	1	93.250	.011	.915
	Treatment x Time	7	623.96	1.424	.193	7	623.814	1.417	.195
	Treatment x Site	1	93.86	.670	.415	-	-	-	-

The figure below illustrates the site-by-treatment interaction for the primary and key secondary drinking outcomes (Purple bars represent psilocybin treatment participants; black bars represent diphenhydramine treated participants).

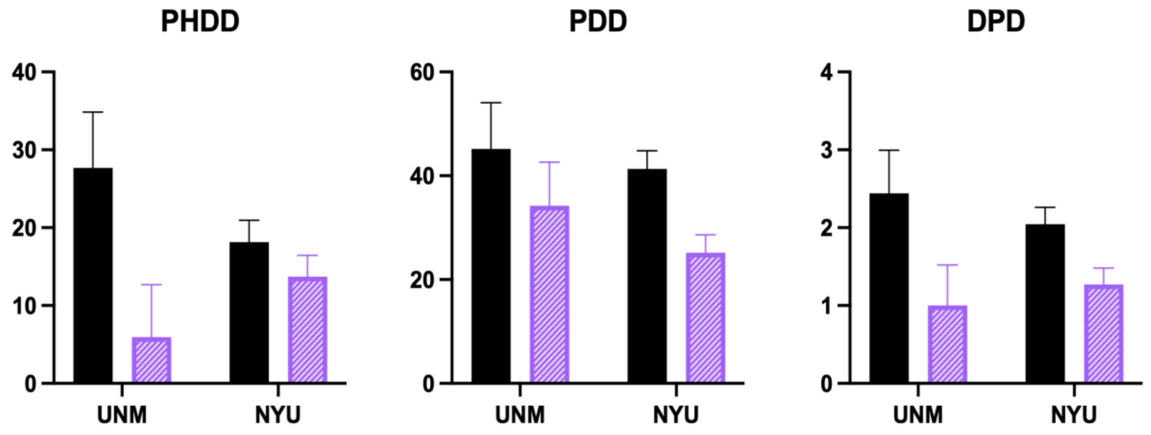

Because the initial models returned evidence for a potential site-by-treatment interaction and produced varying estimates of treatment effect across the drinking metrics analyzed (drinks per day, percent drinking days, percent heavy drinking days, and drinks per drinking day in non-abstinent participants), which were then substantially impacted by inclusion/removal of the site-by-treatment interaction term in a way that was directionally inconsistent (i.e. some effect estimates increased in size and some decreased in size), it is not possible to provide a reasonably accurate estimate of the effect of treatment on drinking based on these results alone.

To resolve this indeterminacy, the PI (Dr. Michael Bogenschutz) met with the trial's Primary Statistician (Dr. Sarah Mennenga) and Senior/Supervising Statistician (Dr. Eugene Laska) to discuss the intent and impact of the parameter refinement step described above, and how analysis for the primary outcome associated with this trial should proceed. The substantial variability across the site-by-treatment and treatment effect estimates for each drinking metric prompted the decision to evaluate the treatment effect in a single multivariate model including each drinking outcome that could be meaningfully represented in each trial participant (percent heavy drinking days, percent drinking days, and drinks per day). This was done to answer the question of what effect psilocybin versus diphenhydramine randomization had on *drinking* as a larger construct than its component parts. Marginal effects will be decomposed, with treatment effect at weeks 9-12 on percent heavy drinking days again designated as the primary contrast. We will not base inclusion of any parameter on its p-value, a decision that is in agreement with extensive literature establishing that: 1) p-values should not be interpreted as measures of effect size [1], 2) discarding effects or classifying them as meaningless based solely on p-values being greater than 0.05 leads to scientific error and apparent irreproducibility (especially in situations where the model is underpowered, such as in our estimate of a site-by-treatment interaction)[2], and 3) in order to be consistent with use and interpretation recommendations set forth by Fisher, larger p-values should be interpreted as offering some evidence against the null hypothesis [3,4]. Specifically, multivariate repeated measures analysis of variance (RM MANOVA) including fixed, categorical effects of treatment, assessment, and site; site-by-treatment and treatment-by-assessment interactions; fixed covariates for each dependent measure (week 1-4 PDD, PHDD, DPD); and monthly values of PDD, PHDD, and DPD (weeks 5-8, 9-12, 13-16, 17-20, 21-24, 25-28, 29-32, 32-36) as a nested multivariate dependent measure, will be used to assess the effect of psilocybin treatment on primary and secondary outcomes.

References

1. Griffiths, P. & Needleman, J. Statistical significance testing and p-values: Defending the indefensible? A discussion paper and position statement. *Int. J. Nurs. Stud.* **99**, 103384 (2019).

2. Van Calster, B., Steyerberg, E. W., Collins, G. S. & Smits, T. Consequences of relying on statistical significance: Some illustrations. *Eur. J. Clin. Invest.* **48**, e12912 (2018).

3. Amrhein, V., Korner-Nievergelt, F. & Roth, T. The earth is flat (p > 0.05): significance thresholds and the crisis of unreplicable research. *PeerJ* **5**, (2017).

4. Amrhein, V., Greenland, S. & McShane, B. Scientists rise up against statistical significance. *Nature* **567**, 305–308 (2019).
