# Supplementary material for: Percentage of Heavy Drinking Days Following Psilocybin-Assisted Psychotherapy vs Placebo in the Treatment of Adult Patients With Alcohol Use Disorder: A Randomized Clinical Trial
Source: JAMA Psychiatry. 2022 Aug 24;79(10):953–62. doi: 10.1001/jamapsychiatry.2022.2096 (PMC9403854; doi:10.1001/jamapsychiatry.2022.2096)
Supplement: Supplement 2. — eTable 1. Treatment Effects on Problems Related to Drinking eTable 2. Adverse Events eFigure. Treatment Effects on Cardiovascular Outcomes [file jamapsychiatry-e222096-s002.pdf]

Supplemental Online Content

Bogenschutz MP, Ross S, Bhatt S, et al. Percentage of heavy drinking days following psilocybin-assisted psychotherapy vs placebo in the treatment of adult patients with alcohol use disorder: a randomized clinical trial. *JAMA Psychiatry*. Published online August 24, 2022. doi:10.1001/jamapsychiatry.2022.2096

eTable 1. Treatment Effects on Problems Related to Drinking
eTable 2. Adverse Events
eFigure. Treatment Effects on Cardiovascular Outcomes

This supplemental material has been provided by the authors to give readers additional information about their work.

eTable 1. Treatment Effects on Problems Related to Drinking

a. **Final Visit (including all week 36 assessments)**

	Timepoint	Diphenhydramine			Psilocybin			Treatment Effect		
		n	Mean	Standard Deviation	n	Mean	Standard Deviation	**g**	**95% CI**	**P-Value**
**Physical**	*Baseline*	45	4.76	2.25	47	4.45	2.05			
	Week 36	40	3.08	2.42	44	1.30	1.86	0.83	0.38 to 1.28	<0.001
**Inter-Personal**	*Baseline*	45	3.64	2.87	47	3.51	2.59			
	Week 36	40	2.08	2.41	44	1.16	2.01	0.42	-0.02 to 0.85	0.061
**Intra-Personal**	*Baseline*	45	6.67	2.12	47	6.55	2.07			
	Week 36	40	4.22	2.71	44	2.43	2.36	0.71	0.27 to 1.15	0.002
**Impulse Control**	*Baseline*	45	2.87	2.12	47	2.49	2.03			
	Week 36	40	1.70	1.80	44	0.75	1.64	0.55	0.12 to 0.99	0.013
**Social Responsibility**	*Baseline*	45	3.67	2.88	47	3.26	2.48			
	Week 36	40	1.93	2.34	44	0.95	1.80	0.47	0.04 to 0.91	0.035
**Total Problems**	*Baseline*	45	21.60	9.61	47	20.26	8.89			
	Week 36	40	13.00	10.48	44	6.59	8.80	0.67	0.23 to 1.11	0.003

© 2022 Bogenschutz MP et al. *JAMA Psychiatry.*

b. MMRM Results

	Timepoint	Diphenhydramine			Psilocybin			Treatment Effect		
		n	Mean	SD	n	Mean	SD	**g**	**95% CI**	**P-value**
**Physical**	*Baseline*	45	4.76	2.25	47	4.45	2.05			
	Week 12	22	2.62	2.65	26	2.63	2.38	-0.01	-0.56 to 0.57	.984
	Week 24	25	2.60	2.93	16	0.59	2.32	0.74	0.10 to 1.39	.016
	Week 36	24	2.52	2.90	21	0.24	2.59	0.83	0.22 to 1.44	.006
**Inter-Personal**	*Baseline*	45	3.64	2.87	47	3.51	2.59			
	Week 12	22	1.93	2.50	26	2.03	2.24	-0.04	-0.53 to 0.61	.886
	Week 24	25	2.29	2.84	16	1.47	2.36	0.31	-0.32 to 0.94	.317
	Week 36	24	1.85	2.82	21	1.14	2.64	0.26	-0.33 to 0.85	.382
**Intra-Personal**	*Baseline*	45	6.67	2.12	47	6.55	2.07			
	Week 12	22	4.36	3.18	26	4.17	2.84	0.06	-0.50 to 0.63	.826
	Week 24	25	4.00	3.52	16	1.55	2.80	0.75	0.10 to 1.40	.015
	Week 36	24	3.80	3.48	21	1.45	3.13	0.71	0.10 to 1.31	.019
**Impulse Control**	*Baseline*	45	2.87	2.12	47	2.49	2.03			
	Week 12	22	1.32	1.92	26	1.41	1.72	-0.05	-0.52 to 0.62	.867
	Week 24	25	1.33	2.16	16	0.68	1.72	0.33	-0.31 to 0.96	.287
	Week 36	24	1.46	2.13	21	0.55	1.93	0.45	-0.15 to 1.04	.134
**Social Responsibility**	*Baseline*	45	3.67	2.88	47	3.26	2.48			
	Week 12	22	2.08	2.48	26	1.96	2.22	0.05	-0.51 to 0.62	.856
	Week 24	25	1.84	2.81	16	1.39	2.33	0.17	-0.46 to 0.80	.578
	Week 36	24	1.60	2.79	21	1.10	2.59	0.19	-0.40 to 0.77	.528
**Total Problems**	Baseline	45	21.60	9.61	47	20.26	8.89			
	Week 12	22	12.00	11.00	26	12.06	9.87	-0.01	-0.56 to 0.57	.984
	Week 24	25	11.69	12.34	16	5.65	10.07	0.53	-0.11 to 1.16	.088
	Week 36	24	10.86	12.25	21	4.29	11.22	0.56	-0.04 to 1.15	.062

© 2022 Bogenschutz MP et al. *JAMA Psychiatry.*

Caption: Problems related to alcohol were assessed using the Short Index of Problems (SIP-2R). Mean total scores and subscale scores are shown, along with sample sizes and standard deviations used to calculate Hedge's g and 95% confidence intervals for between-group comparisons at the baseline assessment *(covariate in the model)*, and each follow-up timepoint assessed *(weeks 12, 24, 36).* P values are uncorrected for multiple comparisons. Supplemental Table 2a shows results of post-hoc analysis including all data collected at the final visit of the double-blind observation period, regardless of whether it occurred within the pre-specified window. Supplemental Table 2b shows results of Mixed Models for Repeated Measures (MMRM) analyses including only data from visits occurring within pre-specified windows (See SAP).

© 2022 Bogenschutz MP et al. *JAMA Psychiatry.*

eTable 2: Adverse Events

a. **AEs occurring after first Administration of Medication (Week 4) through Week 36**

SOC	PT	PSILO (N = 48)	PSILO %	DIPHEN (N = 45)	DIPHEN %	TOTAL (N=93)	TOTAL %	P-VALUE
**BLOOD AND LYMPHATIC SYSTEM DISORDERS**								
	Anemia	0	0.0	1	2.2	1	1.1	0.48
	Thrombocytosis	0	0.0	1	2.2	1	1.1	0.48
**CARDIAC DISORDERS**								
	Palpitations	1	2.1	0	0.0	1	1.1	1.00
**EYE DISORDERS**								
	Cataract	1	2.1	0	0.0	1	1.1	1.00
	Photopsia	1	2.1	0	0.0	1	1.1	1.00
**GASTROINTESTINAL DISORDERS**								
	Abdominal pain	1	2.1	0	0.0	1	1.1	1.00
	Abdominal pain upper	2	4.2	0	0.0	2	2.2	0.50
	Constipation	0	0.0	1	2.2	1	1.1	0.48
	Diarrhea	0	0.0	2	4.4	2	2.2	0.48
	Diverticulitis	1	2.1	0	0.0	1	1.1	1.00
	Dyspepsia	1	2.1	0	0.0	1	1.1	1.00
	Food poisoning	1	2.1	0	0.0	1	1.1	1.00
	Mallory-Weiss syndrome	0	0.0	1	2.2	1	1.1	0.48
	Nausea	10	20.8	4	8.9	14	15.1	0.15
	Toothache	1	2.1	0	0.0	1	1.1	1.00
	Vomiting	2	4.2	1	2.2	3	3.3	1.00
**GENERAL DISORDERS AND ADMINISTRATION SITE CONDITIONS**								
	Asthenia	1	2.1	0	0.0	1	1.1	1.00
	Fatigue	1	2.1	0	0.0	1	1.1	1.00
	Influenza like Illness	0	0.0	1	2.2	1	1.1	0.48
	Oedema	0	0.0	1	2.2	1	1.1	0.48
	Pain	1	2.1	3	6.7	4	8.3	0.35
	Peripheral swelling	0	0.0	1	2.2	1	1.1	0.48
	Pyrexia	0	0.0	1	2.2	1	1.1	0.48
**IMMUNE SYSTEM DISORDERS**								
	Dermatitis contact	0	0.0	1	2.2	1	1.1	0.48
	Food allergy	1	2.1	0	0.0	1	1.1	1.00

© 2022 Bogenschutz MP et al. *JAMA Psychiatry.*

SOC	PT	PSILO (N = 48)	PSILO %	DIPHEN (N = 45)	DIPHEN %	TOTAL (N=93)	TOTAL %	P-VALUE
**INFECTIONS AND INFESTATIONS**								
	Bronchitis	0	0.0	3	6.7	3	3.2	0.11
	Bronchitis bacterial	0	0.0	1	2.2	1	1.1	0.48
	Corona virus infection	2	4.2	0	0.0	2	2.2	0.50
	Eye infection	0	0.0	1	2.2	1	1.1	0.48
	Fungal infection	0	0.0	1	2.2	1	1.1	0.48
	Gingivitis	1	2.1	1	2.2	2	2.2	1.00
	Influenza	1	2.1	2	4.4	3	3.2	0.61
	Pneumonia	2	4.2	0	0.0	2	2.2	0.50
	Upper respiratory tract infection	2	4.2	0	0.0	2	2.2	0.50
	Viral upper resp. tract infection	2	4.2	3	6.7	5	5.4	0.67
**INJURY, POISONING, AND PROCEDURAL COMPLICATIONS**								
	Alcohol poisoning	2	4.2	0	0.0	2	2.2	0.50
	Limb injury	1	2.1	0	0.0	1	1.1	1.00
	Muscle strain	1	2.1	0	0.0	1	1.1	1.00
	Traumatic lung injury	0	0.0	1	2.2	1	1.1	0.48
**INVESTIGATIONS**								
	Biopsy cervix	1	2.1	0	0.0	1	1.1	1.00
	Blood pressure diastolic increased	1	2.1	0	0.0	1	1.1	1.00
	Blood pressure increased	1	2.1	0	0.0	1	1.1	1.00
**METABOLISM AND NUTRITION DISORDERS**								
	Hyponatremia	0	0.0	1	2.2	1	1.1	0.48
**MUSCULOSKELETAL AND CONNECTIVE TISSUE DISORDERS**								
	Arthralgia	0	0.0	1	2.2	1	1.1	0.48
	Back pain	1	2.1	3	6.7	4	4.3	0.35
	Musculoskeletal pain	1	2.1	1	2.2	2	2.2	1.00
	Myalgia	1	2.1	0	0.0	1	1.1	1.00
	Pain in extremity	0	0.0	1	2.2	1	1.1	0.48
**NEOPLASMS BENIGN, MALIGNANT AND UNSPECIFIED**								
	Malignant melanoma	0	0.0	1	2.2	1	1.1	0.48
**NERVOUS SYSTEM DISORDERS**								
	Dizziness	1	2.1	1	2.2	2	2.2	1.00
	Headache	21	43.8	2	4.4	23	24.7	**< 0.01**
	Hypoesthesia	0	0.0	1	2.2	1	1.1	0.48

© 2022 Bogenschutz MP et al. *JAMA Psychiatry.*

SOC	PT	PSILO (N = 48)	PSILO %	DIPHEN (N = 45)	DIPHEN %	TOTAL (N=93)	TOTAL %	P-VALUE
	Migraine	0	0.0	1	2.2	1	1.1	0.48
	Psychomotor hyperactivity	1	2.1	0	0.0	1	1.1	1.00
	Sedation	0	0.0	1	2.2	1	1.1	0.48
	Sinus headache	0	0.0	2	4.4	2	2.2	0.23
**PSYCHIATRIC DISORDERS**								
	Alcohol withdrawal syndrome	1	2.1	2	4.4	3	3.2	0.61
	Anger	0	0.0	1	2.2	1	1.1	0.48
	Anxiety	7	14.6	1	2.2	8	8.6	**0.06**
	Depressed mood	3	6.3	2	4.4	5	5.4	1.00
	Depression	2	4.2	2	4.4	4	4.3	1.00
	Dysphoria	1	2.1	0	0.0	1	1.1	1.00
	Illusion	1	2.1	0	0.0	1	1.1	1.00
	Insomnia	3	6.3	2	4.4	5	5.4	1.00
	Restlessness	0	0.0	1	2.2	1	1.1	0.48
	Suicidal Ideation	4	8.3	3	6.7	7	7.5	1.00
**RENAL AND URINARY DISORDERS**								
	Urinary incontinence	1	2.1	0	0.0	1	1.1	1.00
**REPRODUCTIVE SYSTEM AND BREAST DISORDERS**								
	Testicular pain	1	2.1	0	0.0	1	1.1	1.00
**RESPIRATORY, THORACIC, AND MEDIASTINAL DISORDERS**								
	Cough	0	0.0	1	2.2	1	1.1	0.48
	Dyspnea	1	2.1	0	0.0	1	1.1	1.00
	Hyperventilation	1	2.1	0	0.0	1	1.1	1.00
	Lower resp. tract congestion	0	0.0	2	4.4	2	2.2	0.23
	Nasal congestion	1	2.1	0	0.0	1	1.1	1.00
	Oropharyngeal pain	1	2.1	2	4.4	3	3.2	0.61
	Rhinorrhea	0	0.0	1	2.2	1	1.1	0.48
	Sinus congestion	0	0.0	1	2.2	1	1.1	0.48
**SOCIAL CIRCUMSTANCES**								
	Sexual abuse	0	0.0	1	2.2	1	1.1	0.48
**SURGICAL AND MEDICAL PROCEDURES**								
	Arthoscopic surgery	0	0.0	1	2.2	1	1.1	0.48
	Endodontic procedure	0	0.0	1	2.2	1	1.1	0.48
	Skin cosmetic procedure	1	2.1	0	0.0	1	1.1	1.00

© 2022 Bogenschutz MP et al. *JAMA Psychiatry.*

© 2022 Bogenschutz MP et al. *JAMA Psychiatry.*

b. Treatment-Emergent Adverse Events Occurring within 48 Hours of Medication Administration

SOC	PT	PSILO (N = 48)	PSILO %	DIPHEN (N = 45)	DIPHEN %	TOTAL (N=93)	TOTAL %	P-VALUE
**CARDIAC DISORDERS**								
	Palpitations	1	2.1	0	0.0	1	1.1	1.00
**GASTROINTESTINAL DISORDERS**								
	Abdominal pain	1	2.1	0	0.0	1	1.1	1.00
	Abdominal pain upper	1	2.1	0	0.0	1	1.1	1.00
	Dyspepsia	1	2.1	0	0.0	1	1.1	1.00
	Nausea	10	20.8	3	6.7	13	14.1	**0.07**
	Vomiting	2	4.2	1	2.2	3	3.3	1.00
**GENERAL DISORDERS AND ADMINISTRATION SITE CONDITIONS**								
	Asthenia	1	2.1	0	0.0	1	1.1	1.00
	Fatigue	1	2.1	0	0.0	1	1.1	1.00
	Edema	0	0.0	1	2.2	1	1.1	0.48
	Pain	1	2.1	1	2.2	2	2.2	1.00
**INVESTIGATIONS**								
	Blood pressure diastolic increased	1	2.1	0	0.0	1	1.1	1.00
	Blood pressure increased	1	2.1	0	0.0	1	1.1	1.00
**MUSCULOSKELETAL AND CONNECTIVE TISSUE DISORDERS**								
	Arthralgia	0	0.0	1	2.2	1	1.1	0.48
	Back pain	1	2.1	1	2.2	2	2.2	1.00
	Musculoskeletal pain	1	2.1	0	0.0	1	1.1	1.00
**NERVOUS SYSTEM DISORDERS**								
	Dizziness	1	2.1	1	2.2	2	2.2	1.00
	Headache	21	43.8	1	2.2	22	23.9	**< 0.01**
	Psychomotor hyperactivity	1	2.1	0	0.0	1	1.1	1.00
	Sedation	0	0.0	1	2.2	1	1.1	0.48
**PSYCHIATRIC DISORDERS**								
	Anger	0	0.0	1	2.2	1	1.1	0.48
	Anxiety	4	8.3	0	0.0	4	4.3	0.12
	Depressed mood	0	0.0	1	2.2	1	1.1	0.48
	Depression	1	2.1	0	0.0	1	1.1	1.00
	Dysphoria	1	2.1	0	0.0	1	1.1	1.00
	Illusion	1	2.1	0	0.0	1	1.1	1.00
	Insomnia	3	6.3	2	4.4	5	5.4	1.00

© 2022 Bogenschutz MP et al. *JAMA Psychiatry.*

SOC	PT	PSILO (N = 48)	PSILO %	DIPHEN (N = 45)	DIPHEN %	TOTAL (N=93)	TOTAL %	P-VALUE
	Restlessness	0	0.0	1	2.2	1	1.1	0.48
	Suicidal Ideation	1	2.1	0	0.0	1	1.1	1.00
**RENAL AND URINARY DISORDERS**								
	Urinary incontinence	1	2.1	0	0.0	1	1.1	1.00
	Hyperventilation	1	2.1	0	0.0	1	1.1	1.00
	Oropharyngeal pain	0	0.0	1	2.2	1	1.1	0.48
**SURGICAL AND MEDICAL PROCEDURES**								
	Endodontic procedure	0	0.0	1	2.2	1	1.1	0.48

Caption: Table 3a lists frequency and percentage of participants experiencing adverse events for all adverse events occurring during the 32-week double-blind observation period following the first administration of study medication. Table 3b lists frequency and percentage of participants experiencing adverse events during the 48 hours following study drug administration (Session 1 or Session 2) the first administration of study medication Adverse events are organized by MedDRA System Class (SOC) and preferred term (PT). P-values from Fisher's exact tests are shown for descriptive purposes. Values less than 0.1 are shown in bold.

There were 34 duplicates AEs (PTs occurring more than once in an individual) during the double-blind follow-up period and 14 duplicates within 48 hours of drug administration. For the entire 32-week period, the duplicates were Diarrhea (2 AEs in the diphenhydramine group), Nausea (3 AEs in the psilocybin group, 1 AE in the diphenhydramine group), Upper respiratory tract infection (1 AE in the psilocybin group), Viral upper respiratory tract infection (1 AE in the diphenhydramine group), Alcohol poisoning (1 AE in the psilocybin group), Back pain (1 AE in the psilocybin group, 2 AEs in the diphenhydramine group), Dizziness (1 AE in the diphenhydramine group), Headache (12 AEs in the psilocybin group, 1 AE in the diphenhydramine group), Alcohol withdrawal syndrome (2 AEs in the diphenhydramine group), Anxiety (1 AE in the psilocybin group, 1 AE in the diphenhydramine group), Depressed mood (1 AE in the diphenhydramine group), Depression (1 AE in the psilocybin group), and Suicidal ideation (1 AE in the psilocybin group, 1 AE in the diphenhydramine group). For the 48 hours following drug administration, the duplicates were Nausea (3 AEs in the psilocybin group), Dizziness (1 AE in the diphenhydramine group), and Headache (9 AEs in the psilocybin group, 1 AE in the diphenhydramine group).

© 2022 Bogenschutz MP et al. *JAMA Psychiatry.*

eFigure. Treatment Effects on Cardiovascular Outcomes

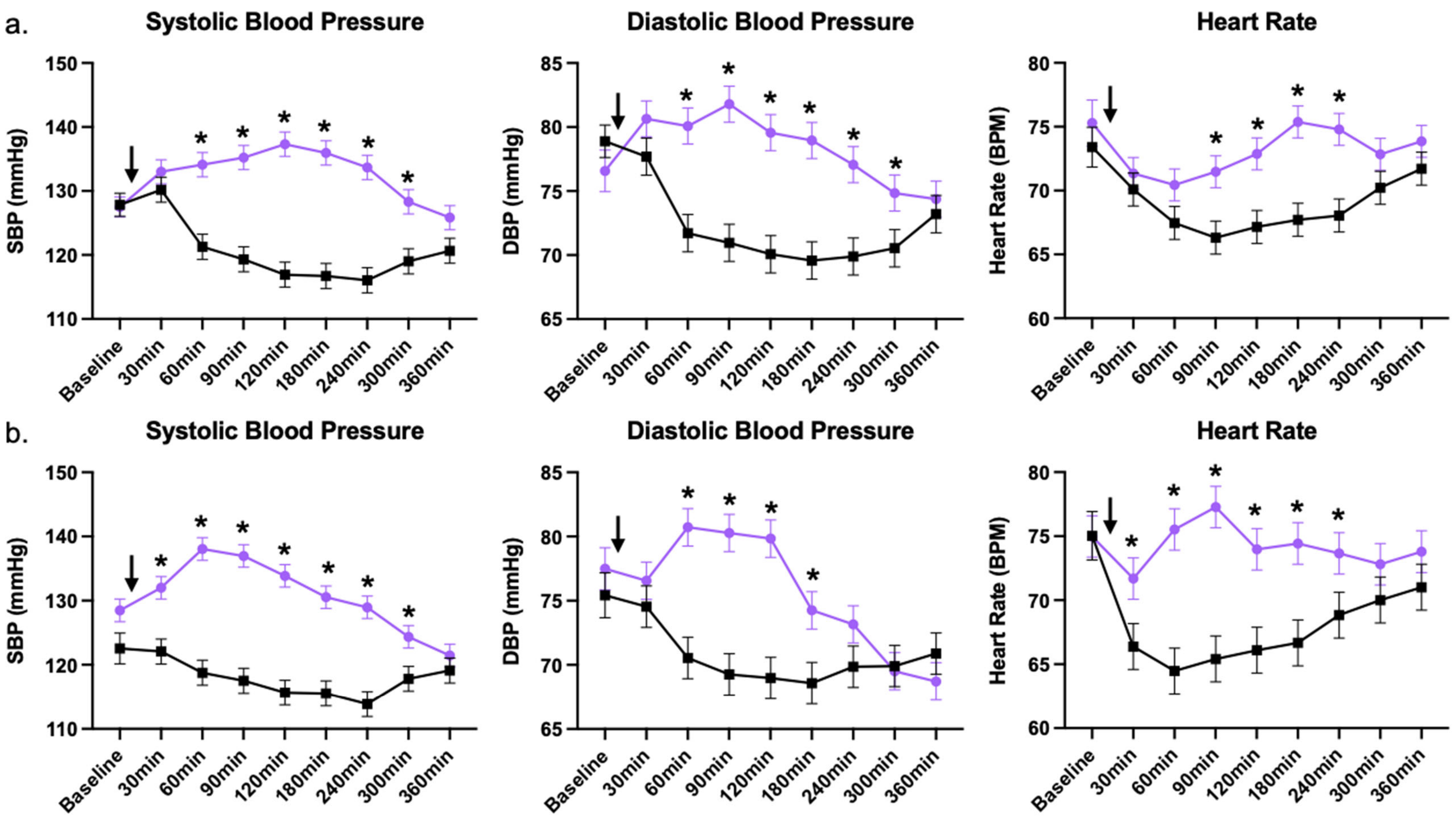

Caption: Mean and SE estimates for participants who received psilocybin (purple) and diphenhydramine (black) are illustrated for baseline (just prior to medication administration; covariate in each model), and eight timepoints following medication administration *(30, 60, 90, 120, 180, 240, 300, 360min after treatment administration)*. Participants who receive psilocybin are shown Arrows represent double-blind medication administration sessions.

© 2022 Bogenschutz MP et al. *JAMA Psychiatry.*

*p<0.05 for Treatment x Time interactions in first medication session (systolic blood pressure: $F_{(7, 651)}$= 10.02, p<0.001; diastolic blood pressure: $F_{(7, 651)}$= 5.48, p<0.001; and heart rate: $F_{(1, 85)}$= 2.89, p=0.01) and second medication session (systolic blood pressure: $F_{(7, 544)}$= 10.59, p<0.001; diastolic blood pressure: $F_{(7, 544)}$= 11.16, p<0.001; and heart rate: $F_{(7, 545)}$= 4.74, p<0.001].

© 2022 Bogenschutz MP et al. *JAMA Psychiatry.*
