# Supplementary material for: Percentage of Heavy Drinking Days Following Psilocybin-Assisted Psychotherapy vs Placebo in the Treatment of Adult Patients With Alcohol Use Disorder: A Randomized Clinical Trial
Source: JAMA Psychiatry. 2022 Aug 24;79(10):953–62. doi: 10.1001/jamapsychiatry.2022.2096 (PMC9403854; doi:10.1001/jamapsychiatry.2022.2096)
Supplement: Supplement 3. — Data sharing statement [file jamapsychiatry-e222096-s003.pdf]

Data Sharing Statement

Bogenschutz. Psilocybin-Assisted Treatment of Alcohol Use Disorder. *JAMA Psychiatry*. Published August 24, 2022. doi:10.1001/jamapsychiatry.2022.2096

Data
Data available: Yes
Data types: Deidentified participant data, Data dictionary
How to access data: Submit requests to the corresponding author for review and approval.
When available: With publication

Supporting Documents
Document types: None

Additional Information
Who can access the data: researchers whose proposed use of the data has been approved
Types of analyses: Specified approved purposes
Mechanisms of data availability: with a signed data access agreement
